# Triapine Analogues and Their Copper(II) Complexes: Synthesis, Characterization, Solution Speciation, Redox Activity, Cytotoxicity, and mR2 RNR Inhibition

**DOI:** 10.1021/acs.inorgchem.1c01275

**Published:** 2021-07-19

**Authors:** Iuliana Besleaga, Iryna Stepanenko, Tatsiana V. Petrasheuskaya, Denisa Darvasiova, Martin Breza, Marta Hammerstad, Małgorzata A. Marć, Alexander Prado-Roller, Gabriella Spengler, Ana Popović-Bijelić, Eva A. Enyedy, Peter Rapta, Anatoly D. Shutalev, Vladimir B. Arion

**Affiliations:** † Institute of Inorganic Chemistry, University of Vienna, Währinger Strasse 42, A-1090 Vienna, Austria; ‡ Department of Inorganic and Analytical Chemistry, Interdisciplinary Excellence Centre, University of Szeged, Dóm tér 7, H-6720 Szeged, Hungary; § MTA-SZTE Lendület Functional Metal Complexes Research Group, University of Szeged, Dóm tér 7, H-6720 Szeged, Hungary; ∥ Institute of Physical Chemistry and Chemical Physics, Faculty of Chemical and Food Technology, Slovak University of Technology in Bratislava, Radlinského 9, SK-81237 Bratislava, Slovak Republic; ⊥ Section for Biochemistry and Molecular Biology, Department of Biosciences, University of Oslo, P.O. Box 1066, Blindern, NO-0316 Oslo, Norway; # Department of Medical Microbiology, Albert Szent-Györgyi Health Center and Faculty of Medicine, University of Szeged, Dóm tér 10, 6725 Szeged, Hungary; ∇ Faculty of Physical Chemistry, University of Belgrade, Studentski trg 12-16, 11158 Belgrade, Serbia; ○ N. D. Zelinsky Institute of Organic Chemistry, Russian Academy of Sciences, 47 Leninsky Avenue, 119991 Moscow, Russian Federation

## Abstract

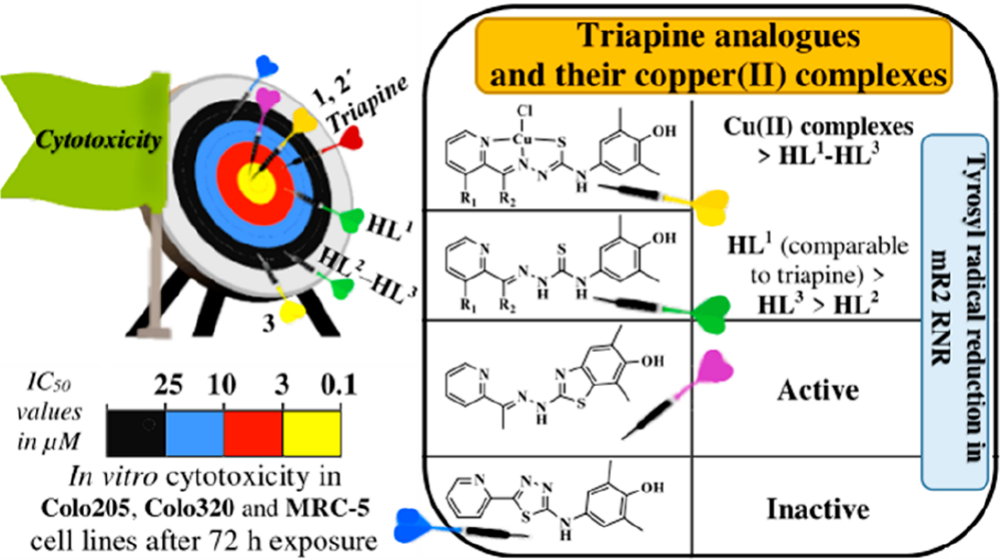

Three new thiosemicarbazones (TSCs) **HL**^**1**^–**HL**^**3**^ as triapine analogues bearing a redox-active phenolic moiety at the terminal nitrogen atom were prepared. Reactions of **HL**^**1**^–**HL**^**3**^ with CuCl_2_·2H_2_O in anoxic methanol afforded three copper(II) complexes, namely, **Cu(HL**^**1**^**)Cl**_**2**_ (**1**), [**Cu(L**^**2**^**)Cl]** (**2′**), and **Cu(HL**^**3**^**)Cl**_**2**_ (**3**), in good yields. Solution speciation studies revealed that the metal-free ligands are stable as **HL**^**1**^–**HL**^**3**^ at pH 7.4, while being air-sensitive in the basic pH range. In dimethyl sulfoxide they exist as a mixture of *E* and *Z* isomers. A mechanism of the *E/Z* isomerization with an inversion at the nitrogen atom of the Schiff base imine bond is proposed. The monocationic complexes **[Cu(L**^**1**–**3**^**)]**^**+**^ are the most abundant species in aqueous solutions at pH 7.4. Electrochemical and spectroelectrochemical studies of **1**, **2′**, and **3** confirmed their redox activity in both the cathodic and the anodic region of potentials. The one-electron reduction was identified as metal-centered by electron paramagnetic resonance spectroelectrochemistry. An electrochemical oxidation pointed out the ligand-centered oxidation, while chemical oxidations of **HL**^**1**^ and **HL**^**2**^ as well as **1** and **2′** afforded several two-electron and four-electron oxidation products, which were isolated and comprehensively characterized. Complexes **1** and **2′** showed an antiproliferative activity in Colo205 and Colo320 cancer cell lines with half-maximal inhibitory concentration values in the low micromolar concentration range, while **3** with the most closely related ligand to triapine displayed the best selectivity for cancer cells versus normal fibroblast cells (MRC-5). **HL**^**1**^ and **1** in the presence of 1,4-dithiothreitol are as potent inhibitors of mR2 ribonucleotide reductase as triapine.

## Abstract 2

Three triapine analogues **HL**^**1**^−**HL**^**3**^ bearing a phenolic redox-active moiety showed moderate antiproliferative activity, while one of the oxidation products **HL**^**2c′**^**·CH3COOH** revealed high cytotoxicity in Colo205 and Colo320 cancer cell lines. Coordination of **HL**^**1**^−**HL**^**3**^ to copper(II) increased strongly the cytotoxicity, with complex **2′** showing IC_50_ values of 0.181 and 0.159, respectively. The highest cytotoxicity of **2′** is likely due to the highest thermodynamic stability, more negative reduction potential, and the lowest rate of reduction by GSH.

## Introduction

Thiosemicarbazones (TSCs) are known as biologically active compounds with a broad spectrum of pharmacological properties, including anticancer activity.^1−4^ These properties can be modulated by coordination to physiologically relevant metal ions.^5,6^ In addition, as versatile ligands, TSCs have tunable electronic and steric properties, which may have a favorable effect on their pharmacological profile.^7−10^ α-N-Heterocyclic TSCs such as 2-formylpyridine TSC (FTSC) and 5-hydroxy-2-formylpyridine TSC were reported to possess anticancer activity several decades ago,^11,12^ and further optimization resulted in the most well-known TSC, 3-aminopyridine-2-carboxaldehyde TSC (triapine). Triapine was tested in more than 30 clinical phase I and II trials and currently is involved in a triapine-cisplatin-radiation combination therapy in phase III trial.^13^ Because of the documented side effects (e.g., methemoglobinemia) of triapine and its unfavorable pharmacokinetic profile (e.g., short plasma half-life),^14^ the development of novel TSCs with improved pharmaceutical properties and an established mechanism of action is of high research interest. Notably, two other TSCs, namely, di-2-pyridylketone 4-cyclohexyl-4-methyl-3-thiosemicarbazone (DpC) and 4-(2-pyridinyl)-2-(6,7-dihydro-8(5*H*)-quinolinylidene)-hydrazide (COTI-2), are currently undergoing a phase I evaluation as chemotherapeutic agents.^8,15^

The iron-containing ribonucleotide reductase (RNR) is considered as one of the main targets for triapine and related α-*N*-pyridinecarboxaldehyde TSCs.^16−19^ This enzyme catalyzes the reduction of ribonucleotides to deoxyribonucleotides, and it is particularly important in rapidly dividing cells, such as tumor cells, virally infected cells, and invading bacteria. All these cells share similar properties, such as high proliferation rates, quickly spreading within the host, and aggressive disease progression.^20^ A sustained proliferation requires an increased de novo nucleotide synthesis for DNA replication, making RNR targeting a relevant strategy in the treatment of cancer.^21,22^ RNRs are free radical-containing proteins. One way to control and modulate their reactivity is via quenching the catalytically essential tyrosyl radical Y· located in the small RNR subunit (R2 or NrdB).^23,24^ The radical scavengers and iron-chelating ligands, which are able to destroy the diferric-tyrosyl radical cofactor, with the aim to inhibit R2 RNR, are widely investigated in anticancer research.^25^ In the case of triapine, it has been suggested that the intracellularly formed, highly potent, redox-active iron complex either leads to reactive oxygen species (ROS) formation, which are then responsible for tyrosyl radical quenching, or that the iron(II) complex itself is able to directly reduce the tyrosyl radical.^16^ Besides triapine, several other R2 RNR inhibitors such as hydroxyurea, 3,4-dihydroxybenzohydroxamic acid (Didox), and 3,4,5-trihydroxybenzamidoxime (Trimidox) have entered clinical trials.^26^ Among other potential tyrosyl radical quenchers, *p*-alkoxyphenols (i.e., *p*-methoxyphenol, *p*-ethoxyphenol, *p*-propoxyphenol, and *p*-allyloxyphenol) and pyrogallol as well as 4-mercaptophenol were identified.^27−29^ The mechanism of RNR inhibition by the *p*-alkoxyphenols and pyrogallol was investigated by both experimental techniques (electron paramagnetic resonance (EPR) and UV–visible (UV–vis) spectroscopy) and theoretical tools (molecular docking and molecular dynamics simulations). Among the aminophenols several compounds were tested as anticancer agents, for example, the nonsteroidal anti-inflammatory drug *N*-acetyl-*p*-aminophenol (acetaminophen), which showed antimelanoma activity to prooxidant glutathione (GSH) depletion by the 3-hydroxy-1,4-quinone-imine-metabolite.^29,30^ Fenretinide (a synthetic retinoid derivative) was introduced in clinical trials for the treatment of breast, bladder, renal, and neuroblastoma malignancies due to its antioxidant activities via scavenging radicals.^31^

It is also worth noting that a coordination to copper(II) may significantly augment the cytotoxic activity of TSCs.^6,10^ Copper(II) as an essential trace element is redox-active, biocompatible, and less toxic than nonendogenous heavy metals. The redox metabolism of cancer cells is different from that of healthy cells and is characterized by increased copper levels in an intracellular environment.^32,33^ Moreover, it was recently suggested that the copper(II) TSC complexes, rather than any metal-free TSCs or their cellular metabolites, are responsible for the biological effects in vitro and in vivo.^6^ One of the reasons for the increased antiproliferative activity of copper(II) complexes of TSCs and the selectivity for cancer cells is considered to be the redox cycling between two oxidation states (Cu^2+^ ↔ Cu^+^) in a biologically accessible window of potentials (from −0.4 to +0.8 V vs normal hydrogen electrode (NHE)) and ROS generation.^6,34^ In this context it is also remarkable that a copper-redox cycle mechanism was found to be responsible for the oxidation of phenolic compounds leading ultimately to reactive oxygen-dependent DNA damage.^35^ The same authors suggested that singlet oxygen or a singlet oxygen-like entity (e.g., a copper-peroxide complex) rather than the free hydroxyl radical plays a role in DNA damage.^35^ At the same time it is worth noting that the idea that an efficient redox cycling of copper(II,I) complexes with thiosemicarbazones can be involved in the anticancer mechanism has been recently challenged^36^ by showing that the most resistant to reduction copper(II) thiosemicarbazonates were the most cytotoxic. In addition, the complexes can also dissociate fast, if the thiosemicarbazone has different affinities to copper(II) and copper(I) and can lose the competition for copper(I) to metallothioneins (MT) and glutathione (GSH).^37^

With this background in mind we aimed at (i) attachment of a phenolic moiety at atom N4 of thiosemicarbazide, (ii) investigation of solution speciation, complex formation reactions of new TSCs with copper(II) in solution, and synthesis of copper(II) complexes, (iii) investigation of the reduction/oxidation of TSCs containing this potentially redox active group, namely, the 4-aminophenolic unit, and copper(II) complexes thereof by electrochemical and spectroelectrochemical techniques and by using chemical oxidants, for example, O_2_, *p*-benzoquinone (PBQ), 2,3-dichloro-5,6-dicyano-1,4-benzoquinone (DDQ), and phenyliodine(III) diacetate (PIDA), as two-electron/two proton acceptors and Ag_2_O, along with an analysis of the reversibility of the oxidation process and the number of participating electrons, (iv) identification of the effects of phenolic unit and coordination to copper(II) on the redox activity and cytotoxicity in vitro as well as on the mR2 RNR inhibition and estimation of their potency to act as reductants for a tyrosyl radical with an apparent redox potential of +1000 ± 100 mV versus NHE.^38^

**Chart 1:**
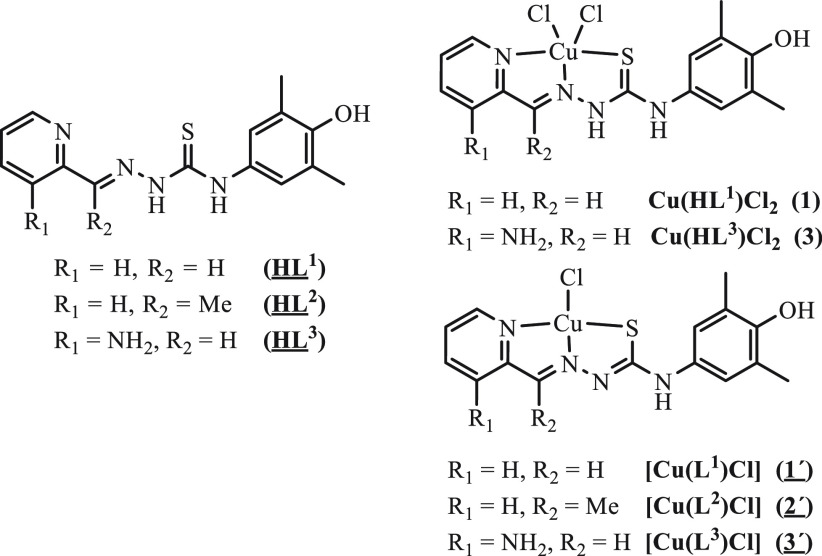
TSCs and Their Copper(II) Complexes Studied in This Worka

**Chart 2:**
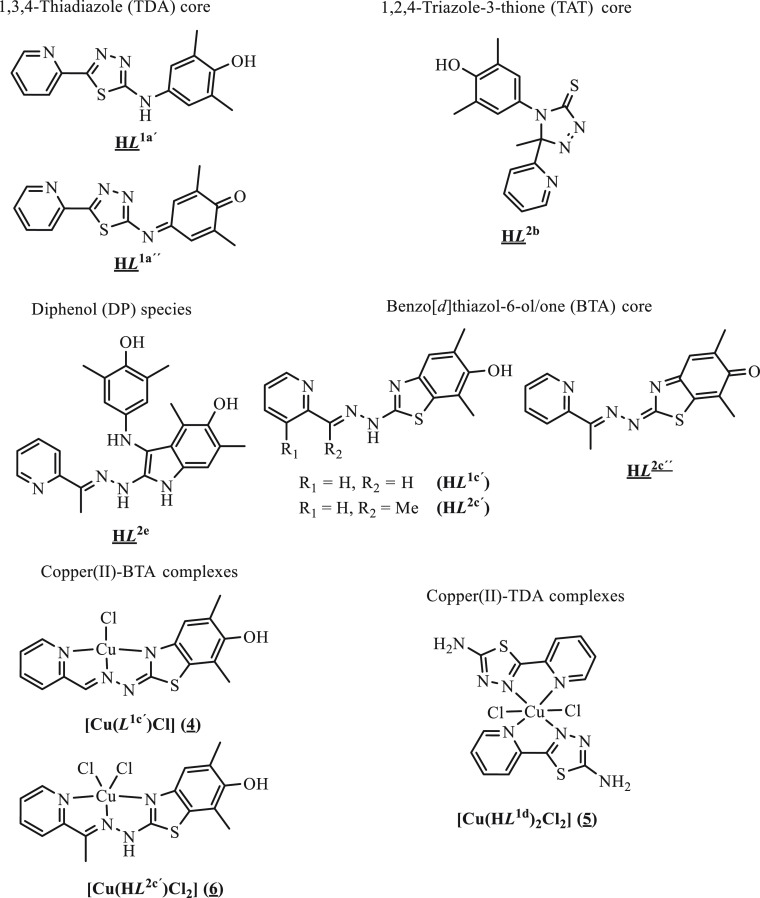
Oxidation Products of **HL**^**1**^ and **HL**^**2**^ and Copper(II) Complexes with Oxidized Ligandsa

**Scheme 1:**
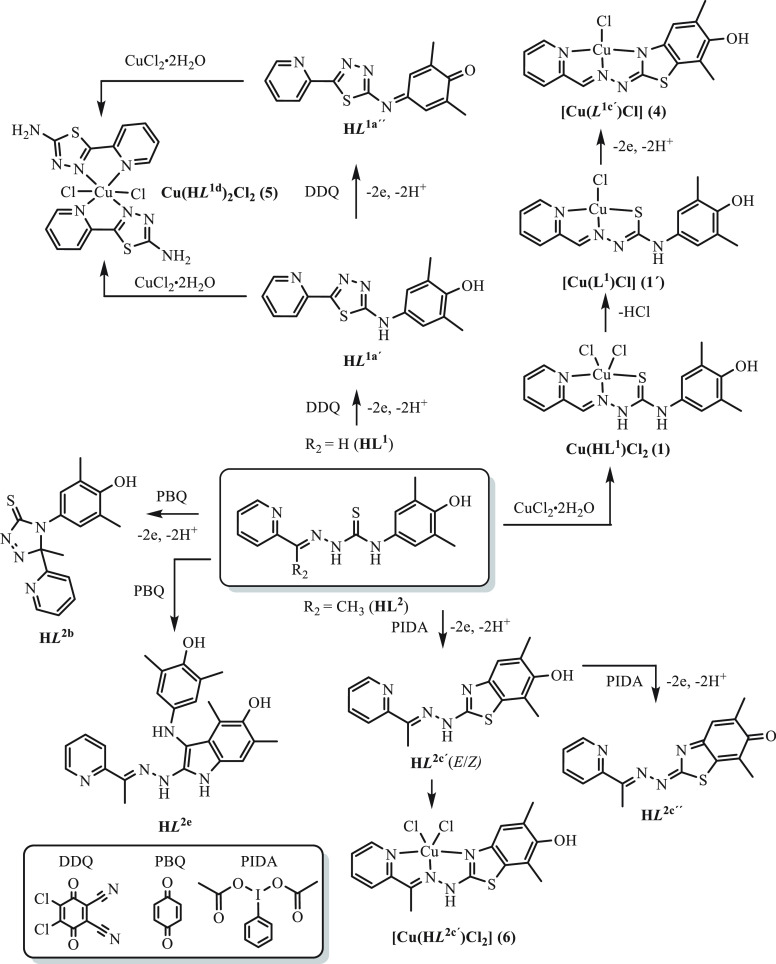
Oxidation Products of **HL**^**1**^ and **HL**^**2**^ along with Those of Copper(II) Complexes The bottom left panel shows the oxidants used.

In this work we report on the synthesis of new triapine derivatives **HL**^**1**^–**HL**^**3**^, which contain a potentially redox-active 4-aminophenolic unit, and of copper(II) complexes **Cu(HL**^**1**^**)Cl**_**2**_ (**1**), **[Cu(L**^**2**^**)Cl]** (**2′**), and **Cu(HL**^**3**^**)Cl**_**2**_ (**3**) (Chart 1). The solution behavior of the new TSCs (**HL**^**1**^–**HL**^**3**^), the mechanism typical for TSC *E*/*Z* isomerization, and the stability and redox properties of both the metal-free ligands and copper(II) complexes (**1**, **2′**, **3**) were also investigated by UV–vis spectrophotometry and UV–vis/EPR spectroelectrochemistry and density functional theory (DFT) calculations. In addition, the two- and four-electron oxidation products **H*L***^**1a′**^ and **H*L***^**1a″**^, respectively, were prepared both electrochemically and by chemical oxidation and used in a complex formation with copper(II). Several oxidation products of **HL**^**2**^ (**H*L***^**2b**^, **H*L***^**2e**^, **H*L***^**2c′**^, and **H*L***^**2c″**^) were prepared by using different oxidation agents. Likewise, copper(II) complexes with oxidized ligands **4**–**6** were obtained (see Chart 2 and Scheme 1). The isolated compounds were characterized by analytical and spectroscopic methods (one-dimensional (1D) and two-dimensional (2D) NMR, UV–vis, IR), electrospray ionization (ESI) mass spectrometry (MS), cyclic voltammetry (CV), and single-crystal X-ray diffraction (SC-XRD). The anticancer activity of the TSCs (**HL**^**1**^–**HL**^**3**^), their oxidized products (**H*L***^**1a′**^, **H*L***^**1a″**^, and **H*L***^**2c′**^**·CH**_**3**_**COOH**), and the copper(II) complexes (**1**, **2′**, and **3**) was tested against two human cancer cell lines (doxorubicin-sensitive Colo205 and the multidrug-resistant Colo320 human colonic adenocarcinoma) and normal human embryonal lung fibroblast cells (MRC-5) along with their mR2 RNR inhibiting ability, and the results are discussed.

a Underlined labels/numbers indicate compounds studied by SC-XRD. The five-coordination of copper(II) in **1** and **3** has not been confirmed by X-ray crystallography.

a Underlined labels/numbers indicate compounds studied by SC-XRD, while the italic *L* denotes an oxidized ligand.

## Experimental section

### Chemicals

2-Formylpyridine, 2-acetylpyridine, and CuCl_2_·2H_2_O were purchased from commercial suppliers and used without further purification. 3-(*tert*-Butoxycarbonyl)amino-2-formylpyridine and 4-(4-hydroxy-3,5-dimethylphenyl)thiosemicarbazide were synthesized as reported previously.^39,40^ KCl, KOH, HCl, and dimethyl sulfoxide (DMSO) were obtained from Reanal. GSH, 2-morpholinoethanesulfonic acid (MES), and 2-[4-(2-hydroxyethyl)piperazin-1-yl]ethanesulfonic acid (HEPES) were purchased from Sigma-Aldrich and used without further purification. Copper(II) stock solution was prepared by the dissolution of CuCl_2_ in water, and its concentration was determined by complexometry with ethylenediaminetetraacetic acid (EDTA). The stock solutions of **HL**^**1**^–**HL**^**3**^ in DMSO were prepared on a weight-in-volume basis.

### 2-Formylpyridine 4-(4-hydroxy-3,5-dimethylphenyl)thiosemicarbazone (**HL**^**1**^**·0.5H**_**2**_**O**)

2-Formylpyridine (0.09 mL, 0.95 mmol) was added to 4-(4-hydroxy-3,5-dimethylphenyl)thiosemicarbazide (200 mg, 0.95 mmol) in ethanol (12 mL), heated at 85 °C for 2 h, concentrated, and left for crystallization at 4 °C. The yellow solid was filtered off, washed with cold ethanol, and dried in vacuo. Yield: 253 mg, 86.1%. Anal. Calcd for C_15_H_16_N_4_OS·0.5H_2_O (*M*_r_ = 309.39): C, 58.23; H, 5.54.; N, 18.11; S, 10.36; Found: C, 57.91; H, 5.45; N, 17.92; S, 10.43%. Positive ion ESI-MS for C_15_H_16_N_4_OS (MeCN/MeOH+1% H_2_O): *m*/*z* 301.11 [HL^1^+H]^+^, 323.09 [HL^1^+Na]^+^, 339.07 [HL^1^+K]^+^, negative ion ESI-MS: *m*/*z* 299.10 [HL^1^–H]^−^. ^1^H NMR (600 MHz, DMSO-*d*_6_, *E* isomer) δ, ppm: 11.86 (s, 1H, H_9_), 10.00 (s, 1H, H_11_), 8.57 (d, *J* = 4.4 Hz, 1H, H_6_), 8.43 (d, *J* = 8.0 Hz, 1H, H_3_), 8.22 (s, 1H, H_18_), 8.16 (s, 1H, H_7_), 7.82 (td, *J* = 7.8, 1.2 Hz, 1H, H_4_), 7.37 (m, 1H, H_5_), 7.02 (s, 2H, H_13_+H_17_), 2.17 (s, 6H, H_19_+H_20_). ^13^C NMR (151 MHz, DMSO-*d*_6_, *E* isomer) δ, ppm: 176.55 (C_10_), 153.31 (C_2_), 151.10 (C_15_), 149.27 (C_6_), 142.51 (C_7_), 136.43 (C_4_), 130.18 (C_12_), 126.26 (C_13_+C_17_), 124.10 (C_5_), 123.84 (C_14_+C_16_), 120.54 (C_3_), 16.62 (C_19_+C_20_). ^15^N NMR (61 MHz, DMSO-*d*_6_, *E* isomer) δ, ppm: 325.04 (N_8_), 315.07 (N_1_), 174.22 (N_9_), 128.93 (N_11_). IR (attenuated total reflectance (ATR), selected bands, *ṽ*_max_): 3107.39, 2950.74, 1531.05, 1477.88, 1428.74, 1201.82, 1105.17, 926.54, 862.73, 761.37, 682.25 cm^–1^. UV–vis (MeOH), λ_max_, nm (ε, M^–1^ cm^–1^): 243 sh, 328 (3516). Single crystals of **HL**^**1**^**·C**_**2**_**H**_**5**_**OH** suitable for X-ray data collection were obtained from the mother liquor.

### 2-Acetylpyridine 4-(4-hydroxy-3,5-dimethylphenyl)thiosemicarbazone (HL^2^·0.2H_2_O)

2-Acetylpyridine (0.21 mL, 1.91 mmol) was added to 4-(4-hydroxy-3,5-dimethylphenyl)thiosemicarbazide (269 mg; 1.27 mmol) in ethanol (8 mL), heated at 85 °C overnight, concentrated, and left for crystallization at 4 °C. The obtained light yellow precipitate was filtered off, washed with cold ethanol, and dried in vacuo. Yield: 271 mg, 67.0%. Anal. Calcd for C_16_H_18_N_4_OS·0.2H_2_O (*M*_r_ = 318.01): C, 60.43; H, 5.83; N, 17.62; S, 10.08. Found: C, 60.47; H, 5.8; N, 17.55; S, 10.13%. Positive ion ESI-MS for C_16_H_18_N_4_OS (*M*_r_ = 314.41) (MeCN/MeOH+1% H_2_O): *m*/*z* 315.13 [HL^2^+H]^+^, 337.11 [HL^2^+Na]^+^, negative ion ESI-MS: *m*/*z* 313.11 [HL^2^–H]^−^. ^1^H NMR (600 MHz, DMSO-*d*_6_, *E* isomer) δ, ppm: 10.46 (s, 1H, H_9_), 9.94 (s, 1H, H_11_), 8.59 (d, *J* = 4.7 Hz, 1H, H_6_), 8.54 (d, *J* = 8.1 Hz, 1H, H_3_), 8.22 (s, 1H, H_18_), 7.79 (td, *J* = 7.8, 1.7 Hz, 1H, H_4_), 7.39 (dd, *J* = 7.2, 4.9 Hz, 1H, H_5_), 7.02 (s, 2H, H_13_+H_17_), 2.44 (s, 3H, H_7′_), 2.17 (s, 6H, H_19_+H_20_). ^13^C NMR (151 MHz, DMSO-*d*_6_, *E* isomer) δ, ppm: 177.36 (C_10_), 154.59 (C_2_), 151.12 (C_15_), 148.54 (C_7_), 148.43 (C_6_), 136.34 (C_4_), 130.36 (C_12_), 126.33 (C_13_+C_17_), 124.00 (C_5_), 123.83 (C_14_+C_16_), 121.18 (C_3_), 16.63 (C_19_+C_20_), 12.31 (C_7′_). ^15^N NMR (61 MHz, DMSO-*d*_6_, *E* isomer) δ, ppm: 312.94 (N_8_), 310.61 (N_1_), 168.53 (N_9_), 129.34 (N_11_). IR (ATR, selected bands, *ṽ*_max_): 3386.87, 3187.76, 1531.57, 1478.45, 1309.19, 1182.40, 1032.57, 942.48, 778.97, 652.93 cm^−1^. UV–vis (MeOH), λ_max_, nm (ε, M^–1^ cm^–1^): 316 (2842), 407 sh. Single crystals of **HL**^**2**^ suitable for X-ray data collection were obtained from the mother liquor.

### 3-Amino-2-formylpyridine 4-(4-hydroxy-3,5-dimethylphenyl)thiosemicarbazone (HL^3^·0.25H_2_O)

To a solution of 3-(*tert*-butoxycarbonyl)amino-2-formylpyridine (210 mg, 0.95 mmol) and 4-(4-hydroxy-3,5-dimethylphenyl)thiosemicarbazide (200 mg, 0.95 mmol) in a mixture of ethanol/water 3:1 (8 mL) was added dropwise 12 M HCl (0.19 mL, 2.28 mmol). This solution was stirred at room temperature for 1 h to give **Boc-HL**^**3**^**·HCl** (C_20_H_25_N_5_O_3_S·HCl, positive ion ESI-MS for C_20_H_25_N_5_O_3_S (*M*_r_ = 415.51) (MeCN/MeOH+1% H_2_O): *m*/*z* 416.18 [Boc-HL^3^+H]^+^, negative ion ESI-MS: *m*/*z* 414.02 [Boc-HL^3^–H]^−^). The Boc-deprotection of **HL**^**3**^ was completed at 85 °C for 7 h with monitoring by ESI-MS (positive ion ESI-MS for C_15_H_17_N_5_OS (*M*_r_ = 315.39) (MeCN/MeOH + 1% H_2_O): *m*/*z* 316.12 [HL^3^+H]^+^, 338.11 [HL^3^+Na]^+^, negative ion ESI-MS: *m*/*z* 314.11 [HL^3^–H]^−^). After ethanol evaporation, the solution was neutralized with a saturated solution of NaHCO_3_ (pH = 8). The precipitate was collected and dried in vacuo. Yield: 267 mg, 87.9%. Anal. Calcd for C_15_H_17_N_5_OS·0.25H_2_O (*M*_r_ = 319.90): C, 56.31; H, 5.51; N, 21.89; S, 10.02. Found: C, 56.33; H, 5.34; N, 21.68; S, 10.29%. ^1^H NMR (600 MHz, DMSO-*d*_6_, *E* isomer) δ, ppm: 11.47 (s, 1H, H_9_), 9.70 (s, 1H, H_11_), 8.39 (s, 1H, H_7_), 8.21 (s, 1H, H_18_), 7.85 (dd, *J* = 4.3, 1.4 Hz, 1H, H_6_), 7.15 (dd, *J* = 8.3, 1.2 Hz, 1H, H_4_), 7.08 (dd, *J* = 8.3, 4.3 Hz, 1H, H_5_), 6.92 (s, 2H, H_13_+H_17_), 6.49 (s, 2H, H_3′_), 2.16 (s, 6H, H_19_+H_20_). ^13^C NMR (151 MHz, DMSO-*d*_6_, *E* isomer) δ, ppm: 176.13 (C_10_), 151.17 (C_15_), 149.23 (C_7_), 143.99 (C_3_), 137.25 (C_6_), 132.97 (C_2_), 130.59 (C_12_), 126.88 (C_13_+C_17_), 124.52 (C_5_), 123.83 (C_14_+C_16_), 122.34 (C_4_), 16.63 (C_19_+C_20_). ^15^N NMR (61 MHz, DMSO-*d*_6_, *E* isomer) δ, ppm: 321.53 (N_1_), 312.8 (N_8_), 174.57 (N_9_), 126.69 (N_11_), 71.10 (N_3′_). IR (ATR, selected bands, *ṽ*_max_): 3456.59, 3347.73, 3142.99, 3002.80, 1615.50, 1547.68, 1512.07, 1299.63, 1248.47, 1189.77, 1143.84, 861.56, 796.22, 685.36 cm^−1^. UV–vis (MeOH), λ_max_, nm (ε, M^–1^ cm^–1^): 299 (1374), 375 (2220), 448 sh. Single crystals of **HL**^**3**^ suitable for X-ray data collection were obtained from the mother liquor.

## Synthesis of the Copper(II) Complexes

### Cu(HL^1^)Cl_2_·0.5H_2_O (1·0.5H_2_O)

CuCl_2_·2H_2_O (128 mg, 0.75 mmol) was added to **HL**^**1**^ (225 mg, 0.75 mmol) in anoxic methanol (10 mL) in a Schlenk tube and stirred at room temperature under argon for 10 min. The reaction mixture was allowed to stand at 4 °C overnight. The dark green precipitate was filtered off under argon, washed with anoxic methanol, and dried in vacuo. Yield: 294 mg, 88.4%. Anal. Calcd for C_15_H_16_N_4_OSCuCl_2_·0.5H_2_O (*M*_r_ = 443.84): C, 40.59; H, 3.86; N, 12.62; S, 7.22. Found: C, 40.73; H, 3.59; N, 12.63; S, 7.19%. Positive ion ESI-MS for C_15_H_16_N_4_OSCuCl_2_ (MeCN/MeOH+1% H_2_O): *m*/*z* 362.03 [Cu(HL^1^)^2+^–H]^+^, negative ion ESI-MS: *m*/*z* 395.99 [Cu(HL^1^)Cl^+^–2H]^−^. IR (ATR, selected bands, *ṽ*_max_): 3480.77, 2989.07, 1610.63, 1479.59, 1269.25, 1229.98, 1189.75, 1025.69, 774.69, 665.85 cm^–1^. UV–vis (MeOH), λ_max_, nm (ε, M^–1^ cm^–1^): 280 (16 800), 376 sh, 422 (18 160). Crystals of **[Cu(L**^**1**^**)Cl]·CH**_**3**_**OH** (**1′·CH**_**3**_**OH**) (*M*_r_ = 398.37) suitable for X-ray diffraction study were grown from an ∼20-fold-diluted reaction mixture in a Schlenk tube under argon upon standing at 4 °C. A recrystallization of **[Cu(HL**^**1**^**)Cl**_**2**_**]** (**1**) in methanol in air afforded a minor amount of X-ray diffraction-quality crystals of **[Cu(*L***^**1c′**^**)Cl]** (**4**).

### [Cu(L^2^)Cl]·0.5H_2_O (2**′**·0.5H_2_O)

CuCl_2_·2H_2_O (129 mg, 0.76 mmol) was added to a solution of **HL**^**2**^ (238 mg, 0.76 mmol) in anoxic methanol (10 mL) in a Schlenk tube. The reaction mixture was stirred at room temperature under argon for 10 min and then allowed to stand at 4 °C overnight. The greenish-brown precipitate was filtered off under argon, washed with anoxic methanol, and dried in vacuo. Yield: 316 mg, 98.8%. Anal. Calcd for C_16_H_17_N_4_OSCuCl·0.5H_2_O (*M*_r_ = 421.40): C, 45.60; H, 4.31; N, 13.30; S, 7.61. Found: C, 45.74; H, 4.03; N, 13.42; S, 7.56%. Positive ion ESI-MS for C_16_H_17_N_4_OSCuCl (MeCN/MeOH+1% H_2_O): *m*/*z* 376.04 [Cu(L^2^)]^+^, negative ion ESI-MS: *m*/*z* 410.00 [Cu(L^2^)Cl–H]^−^. IR (ATR, selected bands, *ṽ*_max_): 3341.84, 3223.12, 1609.18, 1547.35, 1483.22, 1452.56, 1303.41, 1202.82, 1019.61, 846.14, 701.29 cm^–1^. UV–vis (MeOH), λ_max_, nm (ε, M^–1^ cm^–1^): 277 (11 835), 316 sh, 421 (12 953). Crystals of **[Cu(L**^**2**^**)Cl]** (**2′**) suitable for X-ray diffraction study were obtained from an ∼20-fold-diluted reaction mixture under argon in a Schlenk tube at 4 °C.

### Cu(HL^3^)Cl_2_·0.25H_2_O (3·0.25H_2_O)

CuCl_2_·2H_2_O (114 mg, 0.67 mmol) was added to **HL**^**3**^ (210 mg, 0.67 mmol) in anoxic methanol (10 mL) in a Schlenk tube and stirred at room temperature under argon for 10 min. The reaction mixture was allowed to stand at 4 °C overnight. The green precipitate was filtered off under argon, washed with anoxic methanol, and dried in vacuo. Yield: 285 mg, 93.6%. Anal. Calcd for C_15_H_17_N_5_OSCuCl_2_·0.25H_2_O (*M*_r_ = 454.35): C, 39.65; H, 3.88; N, 15.41; S, 7.06. Found: C, 39.58; H, 3.79; N, 15.21; S, 6.98%. Positive ion ESI-MS for C_15_H_17_N_5_OSCuCl_2_ (MeCN/MeOH+1% H_2_O): *m*/*z* 377.04 [Cu(HL^3^)^2+^–H]^+^, negative ion ESI-MS: *m*/*z* 411.00 [Cu(HL^3^)Cl^+^–2H]^−^. IR (ATR, selected bands, *ṽ*_max_): 3422.07, 3340.63, 1647.85, 1569.29, 1480.67, 1223.63, 1185.74, 1023.07, 718.76, 660.61 cm^–1^. UV–vis (MeOH), λ_max_, nm (ε, M^–1^ cm^–1^): 262 (19 564), 288 (17 425), 462 (23 514). Crystals of **[Cu(L**^**3**^**)Cl]·CH**_**3**_**OH**, (**3′·CH**_**3**_**OH**) (*M*_r_ = 413.38) suitable for X-ray diffraction study were grown from an ∼20-fold-diluted reaction mixture in a Schlenk tube under argon at 4 °C.

Details about the synthesis and characterization of oxidized thiosemicarbazones and their copper(II) complexes, X-ray data collection and refinement (Tables S1–S3), elemental analysis, UV–vis titrations, kinetic measurements, lipophilicity determination, spectroelectrochemical studies, in vitro cell studies, 3-(4,5-dimethylthiazol-2-yl)-2,5-diphenyl-tetrazolium bromide (MTT) assays, and tyrosyl radical reduction in mouse R2 RNR protein as well as computational details are given in the Supporting Information (Sections 1 and 2).

## Results and Discussion

### Synthesis and Characterization of **HL**^**1**^**–HL**^**3**^

**Figure 1:**
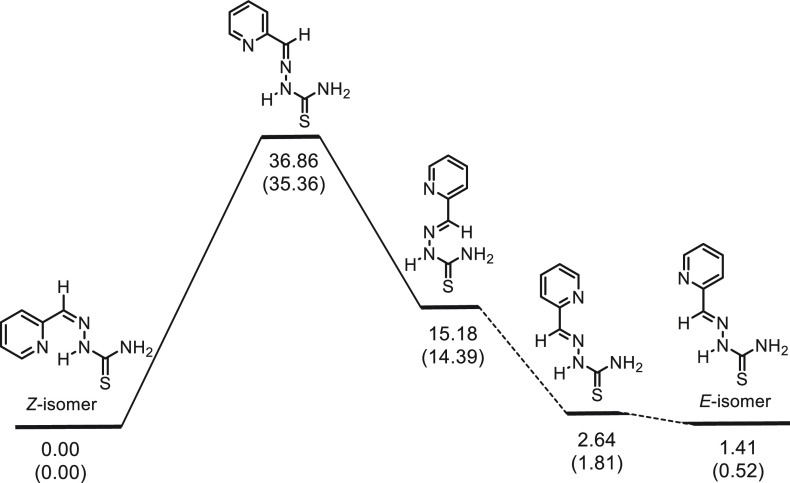
Electronic energy and Gibbs free energy profiles (in kcal/mol) for the transformation of the most stable conformer of (*Z*)-2-formylpyridine thiosemicarbazone into the most stable conformer of (*E*)-2-formylpyridine thiosemicarbazone in DMSO solution. Free energies (in parentheses) at 298 K and 1 atm.

The new TSCs **HL**^**1**^–**HL**^**3**^ were obtained by Schiff base condensation reactions of 4-(4-hydroxy-3,5-dimethylphenyl)thiosemicarbazide^40^ with the corresponding aldehyde (**HL**^**1**^, **HL**^**3**^) or ketone (**HL**^**2**^) in boiling ethanol (**HL**^**1**^, **HL**^**2**^) or ethanol/water (3:1, **HL**^**3**^) in the absence (**HL**^**1**^ and **HL**^**2**^) or in the presence of 12 M HCl (**HL**^**3**^). The hydrochloric acid in this latter case was used for Boc-deprotection of the intermediate **Boc**-**HL**^**3**^. This deprotection reaction was monitored by ESI-MS (disappearance of peaks attributed to [Boc-HL^3^+H]^+^ and [Boc-HL^3^–H]^−^ ions) and completed at 85 °C after 7 h, with yields ranging from 67 to 88%. The formation of **HL**^**1**^–**HL**^**3**^ was confirmed by ESI mass spectra, which showed peaks assigned to ions [HL^1–3^+H]^+^, [HL^1–3^+Na]^+^, and [HL^1–3^–H]^−^. One- and two-dimensional NMR spectra were in agreement with the expected structures for **HL**^**1**^–**HL**^**3**^ of *C*_1_ molecular symmetry. In addition, the spectra indicated the presence of *E* and *Z* isomers in DMSO-*d*_6_, which is typical for thiosemicarbazones,^41−43^ with a significant predominance of *E* isomers (*E*/*Z* = 23:1, 17:1, and 31:1 for **HL**^**1**^–**HL**^**3**^, respectively). The assignment of *E* and *Z* isomers was based on NMR spectra, including ^1^H, ^1^H nuclear Overhauser effect spectroscopy (NOESY), which are presented in more detail in the Supporting Information (see also Schemes S1 and S2 and Tables S4–S6). It is noteworthy that, in contrast to the *E* isomers of **HL**^**1**^–**HL**^**3**^, their *Z* isomers can form an intramolecular hydrogen bond between the pyridine nitrogen and the NH-N group hydrogen, resulting in an increase in the relative stability of these conformers. Indeed, the DFT B3LYP/6-311++G (d,p) calculations for *E*- and *Z*-**HL**^**1**^ in a DMSO solution (the polarizable continuum model (PCM) solvation model) showed that the most stable conformer of *Z*-**HL**^**1**^ lies lower in energy than the most stable conformer of *E*-**HL**^**1**^ (Δ*E* = 1.45 kcal/mol; Δ*G* = 0.76 kcal/mol at 298 K and 1 atm). The calculations also demonstrate that *E*- and *Z*-**HL**^**2**^ are very close in thermodynamic stability (Δ*E* = 0.90 kcal/mol in favor of *Z*-**HL**^**2**^, Δ*G* = 0.00 kcal/mol), and *E*-**HL**^**3**^ is slightly more stable than *Z*-**HL**^**3**^ (Δ*E* = 0.84 kcal/mol, Δ*G* = 0.86 kcal/mol), which can be explained by the presence of an intramolecular hydrogen bond between the 3-NH_2_ group and the aldimine nitrogen in *E*-**HL**^**3**^. Thus, the formation of **HL**^**1**^–**HL**^**3**^ with a large predominance of the *E* isomers indicates that the reactions proceed under a kinetic control. By using DFT B3LYP/6-311++G(d,p) calculations to understand the interconversion between *E* and *Z* isomers of 2-formylpyridine and thiosemicarbazones as model compounds we found out that an isomerization involving a tautomeric shift of the thioamide N2H proton to the pyridine nitrogen followed by a rotation around the formed C–N1 bond, as proposed previously,^44^ is not favored energetically (see the Supporting Information for details). We believe that the most plausible *Z*/*E* isomerization pathway in thiosemicarbazones and semicarbazones involves an inversion at the imine nitrogen.^45^ The intrinsic reaction coordinate (IRC) analysis for one of the aforementioned model compounds revealed that the found transition state connects the desired minima. However, the calculation data obtained show (for more details see the Supporting Information) that the Gibbs free energy barrier for the conversion of the most stable conformer of the *Z* isomer into the *E* isomer is relatively high (Δ*G* = 35.2 kcal/mol in the gas phase, 35.4 kcal/mol in DMSO solution) (Figure 1), which rejects the possibility of an interconversion between the isomers at room temperature.

The redox activity of **HL**^**1**^–**HL**^**3**^ in the anodic region was validated by cyclic voltammetry (vide infra). Their behavior as reductants is also relevant for quenching the tyrosyl radical in the mR2-protein. Therefore, attempts to perform an oxidation of **HL**^**1**^ and **HL**^**2**^ by electrolysis and by chemical oxidation were undertaken.

### Oxidation of TSCs

**Figure 2:**
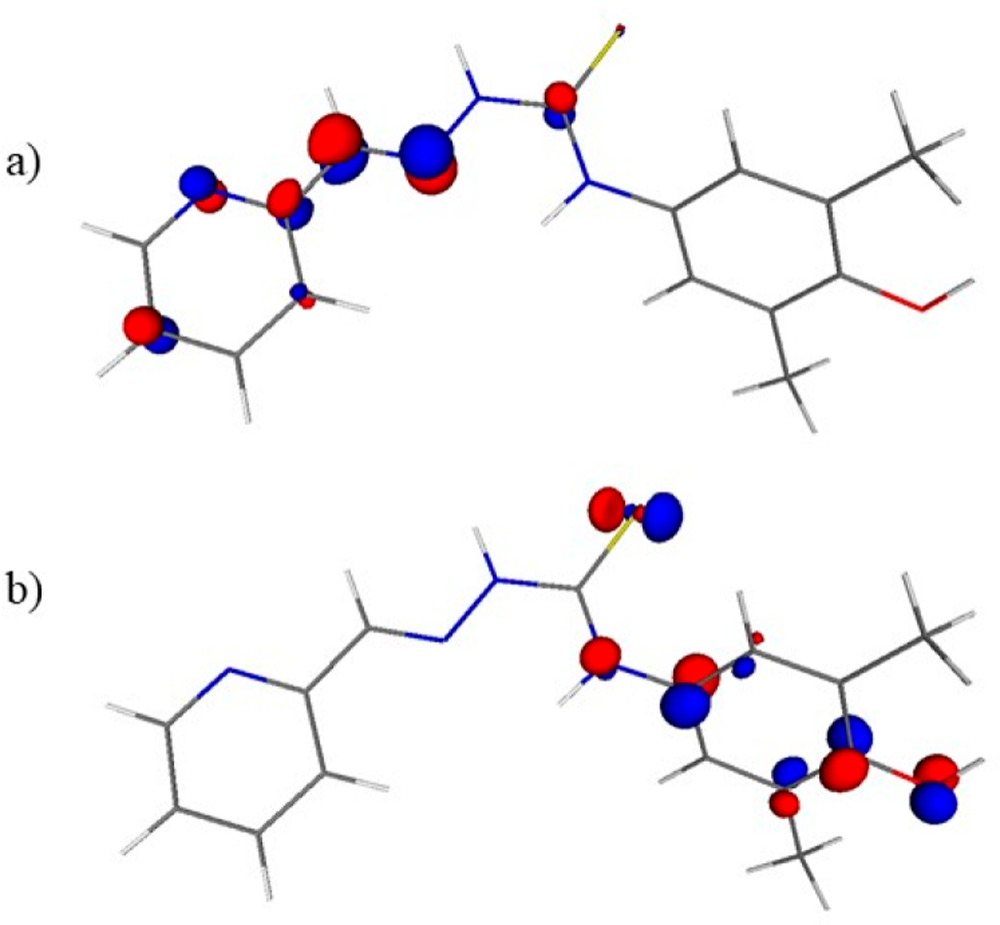
Frontier orbitals in **HL**^**1**^: (a) LUMO and (b) HOMO drawn at 0.1 au isosurface.

The oxidation of different organic molecules with *p*-benzoquinone derivatives is well-documented in the literature.^46^ The reaction of **HL**^**1**^ with DDQ (2e^–^/2H^+^*E*° = +0.887 V vs NHE in an acidic 0.1 M aqueous solution of *p*-TsOH)^47^ in a 1:1 molar ratio resulted in two-electron and four-electron oxidative cyclizations with the major formation of **H*L***^**1a′**^ (60.9%) accompanied by a minor generation of **H*L***^**1a″**^ (<5%), both containing a 1,3,4-thiadiazole ring (Chart 2, Scheme 1). The formation of the 1,3,4-thiadiazole ring occurs via a nucleophilic attack of the sulfur atom to the carbon atom of the aldimine bond of **HL**^**1**^ as evidenced by frontier molecular orbitals with the highest occupied molecular orbital (HOMO) and lowest unoccupied molecular orbital (LUMO) located at opposite sides of the molecule (Figure 2).

The use of a double amount of DDQ led to the formation of the four-electron oxidation product **HL**^**1a″**^ in 71.6% yield. The electrolysis of **HL**^**1**^ at 1000 mV in CH_3_CN versus Ag/AgCl resulted in the same oxidation products (vide infra). Both compounds were characterized by ESI mass spectra, which showed peaks at *m*/*z* 299.17 [H*L*^1**a′**^+H]^+^, 321.16 [H*L*^1a′^+Na]^+^, 297.18 [H*L*^1a**″**^+H]^+^, 319.20 [H*L*^1a**″**^+Na]^+^, and 296.94 [H*L*^1a′^–H]^−^. The more sterically hindered ketimine carbon atom in **HL**^**2**^ was expected to reduce the likelihood of the 1,3,4-thiadiazole ring formation. The reaction of **HL**^**2**^ with DDQ in a 1:1 molar ratio in methanol led to decomposition of the TSC with formation of an unidentified species. When PBQ, a weaker oxidant (2e^–^/2H^+^*E*° = 0.643 V vs NHE in an acidic 0.1 M aqueous solution of *p*-TsOH) than DDQ, was used,^47^ a two-electron oxidative cyclization with the formation of a 1,2,4-triazole-3-thione ring (TAT group, **H*L***^**2b**^) occurred, accompanied by desulfurization of **HL**^**2**^ and conversion into diphenolic species **H*L***^**2e**^ (DP group).^48^ The formation of **H*L***^**2b**^ was confirmed by ESI mass spectra, where peaks corresponding to [H*L*^2b^+H]^+^ (*m*/*z* 313.25), [H*L*^2b^+Na]^+^ (*m*/*z* 335.14), and [H*L*^2b^–H]^−^ (*m*/*z* 310.99) were present. We suppose that the initial step of the reaction of **HL**^**2**^ with PBQ involves a one-electron oxidation of **HL**^**2**^ favored by the character of the HOMO of **HL**^**2**^ (see Figure S1) along with a NH deprotonation to give a highly conjugated N/S-centered free radical (see Scheme S8 in Supporting Information). This radical intermediate transforms into triazole **H*L***^**2b**^ in two steps or undergoes a fragmentation affording 4-isothiocyanato-2,6-dimethylphenol. The phenol reacts with **HL**^**2**^ via an S_E_2 mechanism to form the corresponding thioamide followed by a radical-promoted intermolecular transformation into indole **H*L***^**2e**^ according to a Fukuyama-like indole synthesis^49^ (for a more detailed discussion of the oxidation of **HL**^**2**^ with PBQ see the Supporting Information).

Other oxidation agents (lead tetraacetate, phenyliodine(III) diacetate (PIDA) with *E°* = +1.70 V vs Fc/Fc^+^ in ACN,^50^ and silver(I) oxide) for *N*-alkyl(aryl)-aminocarbonyl-4-aminophenols,^51^ were also used in an attempt to obtain the desired oxidation products with a 1,4-benzoquinone imine moiety (see also Scheme S3, its accompanying explanation, and Figure S2 in the Supporting Information). The exposure of **HL**^**2**^ to 1 equiv of PIDA furnished the two-electron oxidized product **H*L***^**2c′**^ and traces of the four-electron oxidized species **H*L***^**2c″**^. As for **HL**^**1a′**^ and **HL**^**1a″**^, the use of a double amount of oxidant resulted in **H*L***^**2c″**^ as the main oxidation product. ESI mass spectra showed peaks at *m*/*z* 313.21, 310.98 attributed to [H*L*^2c′^+H]^+^, [H*L*^2c′^–H]^−^ as well as 311.12, 309.01 assigned to [H*L*^2c″^+H]^+^, H*L*^2c″^–H]^−^ in line with the loss of two (**H*L***^**2c′**^) or four (**H*L***^**2c″**^) protons when compared to original TSC **HL**^**2**^ (315.13 [HL^2^+H]^+^, 313.11 [HL^2^–H]^−^).

### Characterization of Oxidized Organic Compounds by NMR Spectroscopy

The formation of a 1,3,4-thiadiazole-ring in **H*L***^**1a′**^ and **H*L***^**1a″**^ by an oxidation of **HL**^**1**^ resulted in the disappearance of peaks of the aldimine C*H* proton (H_7_) and N*H* (H_9_) in **H*L***^**1a′**^ and **H*L***^**1a″**^ as well as of the signal of N*H* (H_11_) in **H*L***^**1a″**^. The formation of a 1,4-benzoquinone imine moiety in **H*L***^**1a″**^ was confirmed also by the absence of the O*H* signal, which resonates at 8.08–8.22 ppm in **HL**^**1**^–**HL**^**3**^, **H*L***^**1a′**^ (see Scheme S4 and Tables S4–S6 in the Supporting Information). The ring-closure reaction resulted in a downfield shift of the resonance signal of carbon C_7_, which was directly involved in the 1,3,4-thiadiazole ring formation. The quaternary carbon C_7_ in **H*L***^**1a′**^ and **H*L***^**1a″**^ resonates at 158.40 and 169.98 ppm, respectively, whereas the aldimine *C*H carbon atom C_7_ in **HL**^**1**^ resonates at 142.51 ppm. Analogously, the involvement of the sulfur atom in the 1,3,4-thiadiazole ring led to a downfield shift of the signal of the carbon atom C_10_ (*C*=S) to 166.77 ppm in **H*L***^**1a′**^ and to 171.58 ppm in **H*L***^**1a″**^ when compared to 176.55 ppm in **HL**^**1**^.

The four-electron oxidation of **HL**^**1**^ to **H*L***^**1a″**^ with the formation of the imine N(11)=C(12) bond resulted in strong downfield shift of the resonance signal of carbon C_12_ of 1,4-benzoquinone moiety of **HL**^**1a″**^ (162.21 ppm) when compared to that of carbon C_12_ of phenolic moiety in **HL**^**1**^–**HL**^**3**^, **H*L***^**1a′**^ (130.18–132.53 ppm). In addition, the formation of the carbonyl C(15)=O(18) bond in **H*L***^**1a″**^ has a strong effect on the resonance of carbon atom C_15_, which is strongly downfield-shifted to 187.14 ppm when compared to that in **HL**^**1**^–**HL**^**3**^ and **H*L***^**1a′**^ at 148.97–151.17 ppm. Remarkable shifts of resonance signals for other atoms of the 1,4-benzoquinone moiety in **H*L***^**1a″**^ in comparison to the phenolic moiety in **HL**^**1**^–**HL**^**3**^ and **H*L***^**1a′**^ were also noticed (see the Supporting Information and Scheme S5 therein).

The formation of the benzothiazole ring in **H*L***^**2c′**^ is evidenced by the presence in the ^1^H NMR spectrum of one singlet of the C*H* group and two singlets of methyl groups of an unsymmetrical phenolic moiety with the intensity ratio of 1:3:3 as well as by one N*H* signal at 11.76 ppm in comparison with a number of signals in the spectrum of **HL**^**2**^ (1(N*H*)/1(N*H*)/2(C*H*)/6(C*H*_3_)). Of the two proposed tautomers for **HL**^**2c′**^ (**A** (N(11)*H*) and **B** (N(9)*H*); see Scheme S6 in the Supporting Information) the formation of the *E* isomer of form **B** in DMSO-*d*_6_ was evidenced by the cross-peak between protons of methyl (H_7′_) and N*H* (H_9_) groups in the ^1^H, ^1^H NOESY spectrum. The DFT B3LYP/6-311++G(d,p) calculations showed that the *E* isomer of tautomer **A** is less stable than the *E* isomer of tautomer **B** in a DMSO solution (Δ*E* = 1.58 kcal/mol; Δ*G* = 1.01 kcal/mol at 298 K and 1 atm). We found that, in contrast to **HL**^**1**^–**HL**^**3**^, the *E/Z* isomerization was observed for **H*L***^**2c′**^. As expected in case of **H*L***^**2c′**^**·CH**_**3**_**COOH**, where nitrogen atom N_1_ of the pyridine ring is protonated and prevents the hydrogen-bond formation between H_9_ and N_1_, which is present in the *Z* isomer of **H*L***^**2c′**^, only one set of signals attributed to the *E* isomer was found. The neutral species **H*L***^**2c′**^ in DMSO-*d*_6_ and MeOH-*d*_4_ is present as the *E* isomer, which converts slowly into the *Z* isomer. The process is solvent-dependent. The *E*/*Z* equilibrium was reached in 6 d with a molar ratio of *E*/*Z* isomers of 7.2:1 (DMSO-*d*_6_) and 3:1 (MeOH-*d*_4_) (see Figure S3 in the Supporting Information). The *Z* isomer of **H*L***^**2c′**^ in DMSO-*d*_6_ is characterized by the downfield-shifted proton N*H*(9) due to the hydrogen bond to the pyridine nitrogen atom and resonates at 15.00 ppm (the same proton of the *E* isomer of **H*L***^**2c′**^ is seen at 11.58 ppm). The *Z/E* isomerization of **H*L***^**2c′**^ was also studied in MeOH-*d*_4_ and methanol by ^1^H NMR and UV–vis spectroscopy reaching 1:3.6 molar ratio in 14 d according to NMR spectra (for optical spectra difference see Figure S4). The carbon atom of the methyl group (C_7′_) in the *E* isomers of **H*L***^**2c′**^**·CH**_**3**_**COOH** and **H*L***^**2c′**^ resonates at 12.55 and 12.56 ppm, respectively, whereas in the *Z* isomer of **H*L***^**2c′**^ it resonates at 21.72 ppm. Note that these chemical shifts are consistent with those calculated for *E-* and *Z*-**H*L***^**2c′**^ (8.29 and 23.26 ppm, respectively) by the gauge-independent atomic orbital (GIAO) method at the WC04/6-311+G(2d,p) level of theory using the DFT B3LYP/6-311++G(d,p) optimized geometries (DMSO solution, the PCM solvation model). A similar difference in chemical shifts of the CH_3_ group was also observed for the *E* (12.31 ppm) and *Z* isomers (21.73 ppm) of **HL**^**2**^. The DFT calculation also demonstrated that *E* and *Z* isomers of **H*L***^**2c′**^ have a quite similar stability in a DMSO solution (Δ*G* = 0.11 kcal/mol in favor of the *E* isomer; 298 K, 1 atm). As expected, the pyridine ring carbon atom C_3_ is also sensitive to the hydrogen-bond formation between H_9_ and N_1_ in the *Z* isomer of **H*L***^**2c′**^. The C_3_ signal in the latter is markedly shifted (124.08 ppm) in comparison to C_3_ in the *E* isomer (119.65 ppm). A full assignment of resonances was possible only for **H*L***^**2c′**^**·CH**_**3**_**COOH** (the three quaternary carbons C_12_, C_7_, and C_17_ were identified according to ^1^H, ^13^C HMBC; see Figure S5 in the Supporting Information).

The two-electron oxidation of **H*L***^**2c′**^ to **H*L***^**2c″**^with the formation of the quinone moiety is accompanied by the downfield shift of the resonance signal of carbon C_15_ at 184.43 ppm in comparison to that of C_15_ in **H*L***^**2c′**^**·CH**_**3**_**COOH** at 148.14 ppm, in *E*-**H*L***^**2c′**^ at 148.15 ppm, and in *Z*-**H*L***^**2c′**^ at 148.39 ppm. The lack of the NH signal confirms the formation of the imine N(9)=C(10) bond (see Scheme S7 and Tables S4 and S5 in the Supporting Information).

### Synthesis and Characterization of Copper(II) Complexes

The reaction of **HL**^**1**^–**HL**^**3**^ with CuCl_2_·2H_2_O in anoxic methanol under an argon atmosphere to preclude an eventual oxidation of the ligands by air oxygen in a 1:1 molar ratio at room temperature afforded green-brown solids of the formulas **Cu(HL**^**1**^**)Cl**_**2**_ (**1**), **[Cu(L**^**2**^**)Cl]** (**2′**), and **Cu(HL**^**3**^**)Cl**_**2**_ (**3**) in almost quantitative yields. The formation of these copper(II) complexes was confirmed by elemental analyses and ESI mass spectra. The latter showed peaks attributed to [Cu(L^1,3^)–H]^+^, [Cu(L^1,3^)Cl–H]^−^, or [Cu(L^2^)]^+^ and [Cu(L^2^)Cl–H]^−^. XRD-quality single crystals of **[Cu(L**^**1**–**3**^**)Cl]** (**1′–3′**) were grown from diluted by a factor of ca. 20 reaction mixtures under argon upon standing at 4 °C. Under these conditions the deprotonation of ligands **HL**^**1**^ and **HL**^**3**^ occurred. Attempts to crystallize **1**, **2′**, and **3** in air failed, most likely because of an occurring oxidation of complexes by O_2_.

### Synthesis of the Copper(II) Complexes with Oxidized Ligands

Upon a prolonged standing of a methanolic solution of **Cu(HL**^**1**^**)Cl**_**2**_ (**1**) in air, a minor amount of crystals of **[Cu(*L***^**1c′**^**)Cl]** (**4**) formed, in which the ligand underwent an oxidative dehydrogenation along with the intramolecular cyclization via a C–S coupling reaction between phenolic carbon and thione group into a five-membered thiazole ring, as confirmed by SC-XRD (vide infra). Some rare examples of thiosemicarbazone cyclization with the benzothiazole ring formation due to a coordination to copper(II) were recently reported.^52,53^ A direct complex formation reaction between the prepared benzo[*d*]thiazol-6-ol **H*L***^**2c′**^ and copper(II) chloride produced [**Cu(H*L***^**2c′**^**)Cl**_**2**_**]** (**6**) under an inert atmosphere. The same reaction in air was accompanied by a further oxidation of **H*L***^**2c′**^ with the formation of benzo[*d*]thiazol-6-one (**H*L***^**2c″**^) bound to copper(II). Complex **6** was characterized by the positive ion ESI mass spectrum with a peak at *m*/*z* 374.08 attributed to [Cu(*L*^2c′^)]^+^, whereas the product obtained by an oxidation in air revealed a peak at *m*/*z* 373.06 assigned to [Cu^I^(H*L*^2c″^)]^+^. The peak at *m*/*z* 373.06 was also seen when the reaction mixture of **H*L***^**2c″**^ with CuCl_2_·2H_2_O was subjected to an ESI MS measurement.

The reactions of copper(II) with the oxidized TSCs, namely, 1,3,4-thiadiazole-containing species **H*L***^**1a′**^ and **H*L***^**1a″**^, were monitored by ESI-MS experiments. When CuCl_2_·2H_2_O was allowed to react with **H*L***^**1a′**^ and **H*L***^**1a″**^ in a 1:1 molar ratio, ESI mass spectra of the reaction mixtures indicated the formation of complexes with metal-to-ligand stoichiometry of 1:2, namely, [Cu(H*L*^1a′^)_2_]^+^ and [Cu(H*L*^1a**″**^)_2_]^+^, respectively. Interestingly, under varied reaction conditions (different solvents, air atmosphere, and varied temperature and reaction time, see details in Table S7) the synthesis of copper(II) complex of **H*L***^**1a′**^ resulted in a sequential oxidation of the two ligands, and several oxidized products could be identified based on ESI-MS peaks as [Cu(H*L*^1a′^)_2_]^+^ (*m*/*z* 659.16), [Cu(H*L*^1a′^)(H*L*^1a**″**^)]^+^ (*m*/*z* 657.13), [Cu(H*L*^1a**″**^)_2_]^+^ (*m*/*z* 655.18), [Cu(H*L*^1a′^)(CH_3_CN)]^+^ (*m*/*z* 402.10), [Cu(H*L*^1a**″**^)(CH_3_CN)]^+^ (*m*/*z* 400.10). Moreover, attempts of the chromatographic separation of the obtained compounds (on SiO_2_ with MeOH as eluent) led to a new species [Cu(H*L*^1a′^)(H*L*^1d^)]^+^ (*m*/*z* 537.15), in which one already oxidized ligand **H*L***^**1a′**^ in [Cu(H*L*^1a**″**^)_2_]^+^ lost the phenolic moiety. The complex formation of **H*L***^**1a″**^ in MeOH under heating at 50 °C resulted in two species [Cu(H*L*^1a**″**^)(H*L*^1d^)]^+^ (*m*/*z* 537.15) and [Cu(H*L*^1d^)_2_]^+^ (*m*/*z* 419.08), whereas under prolonged heating (36 h) only [Cu(H*L*^1d^)_2_]^+^ was detected, and the formation of complex [**Cu(H*L***^**1d**^**)**_**2**_**Cl**_**2**_**]** (**5**) was confirmed by SC-XRD.

The potentially redox-active TSC ligands (**HL**^**1**^, (**L**^**2**^)^−^, and **HL**^**3**^) in **1**, **2′**, and **3** proved to react slowly with oxygen in air. Indeed, ESI mass spectra of methanolic solutions of **1**, **2′**, or **3** after a prolonged standing in air showed peaks with *m*/*z* shifted by 2 amu to lower masses in agreement with an oxidative dehydrogenation required for the formation of two-electron oxidation products.

To finally determine the redox status of the 4-aminophenolic moiety, the configurations adopted by the metal-free ligands in the solid state and their protonation level in copper(II) complexes SC-XRD studies were performed.

### X-ray Crystallography of the Metal-Free Ligands **HL**^**1**^**–HL**^**3**^ and Copper(II) Complexes 1′–3′

**Figure 3:**
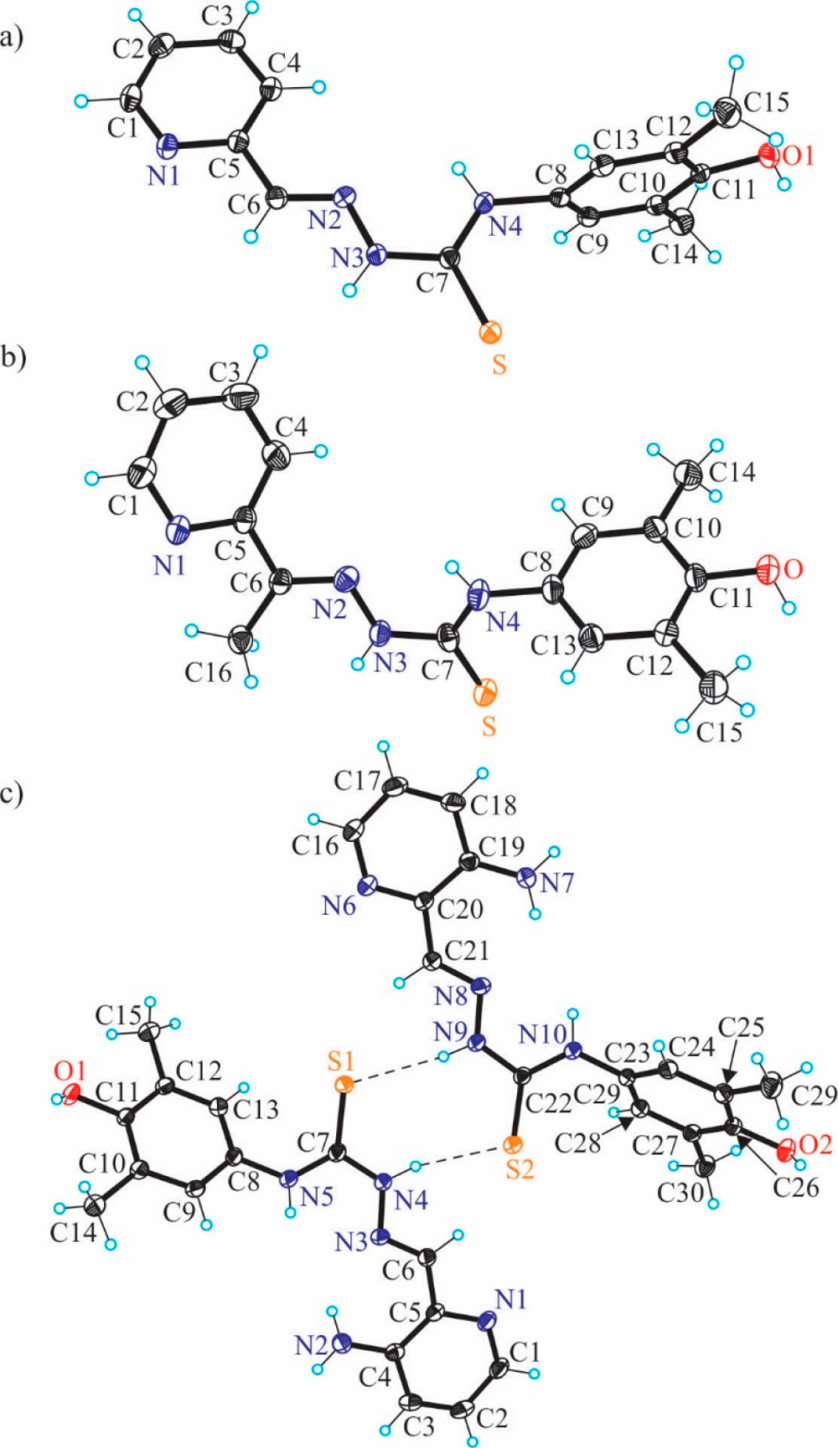
ORTEP views of **HL**^**1**^–**HL**^**3**^ with thermal ellipsoids at the 50% probability level. Selected bond distances (Å) and torsion angles (deg): (a) **HL**^**1**^: C6–N2 1.280(2), N2–N3 1.3701(18), N3–C7 1.357(2), C7–S 1.6839(15), C7–N4 1.331(2), N4–C8 1.442(2), C11–O1 1.3780(19); Θ_C7–N4–C8–C13_ – 88.7(2); (b) **HL**^**2**^: C6–N2 1.287(4), N2–N3 1.374(4), N3–C7 1.363(4), C7–S 1.683(4), C7–N4 1.326(4), N4–C8 1.446(4), C11–O 1.370(4); Θ_C7–N4–C8–C13_ – 78.4(4); (c) **HL**^**3**^: C4–N2 1.361(3), C6–N3 1.288(2), N3–N4 1.385(2), N4–C7 1.343(2), C7–S1 1.695(2), C7–N5 1.342(3), N5–C8 1.430(2), C11–O1 1.380(2); Θ_C7–N5–C8–C13_ 52.5(3).

**Figure 4:**
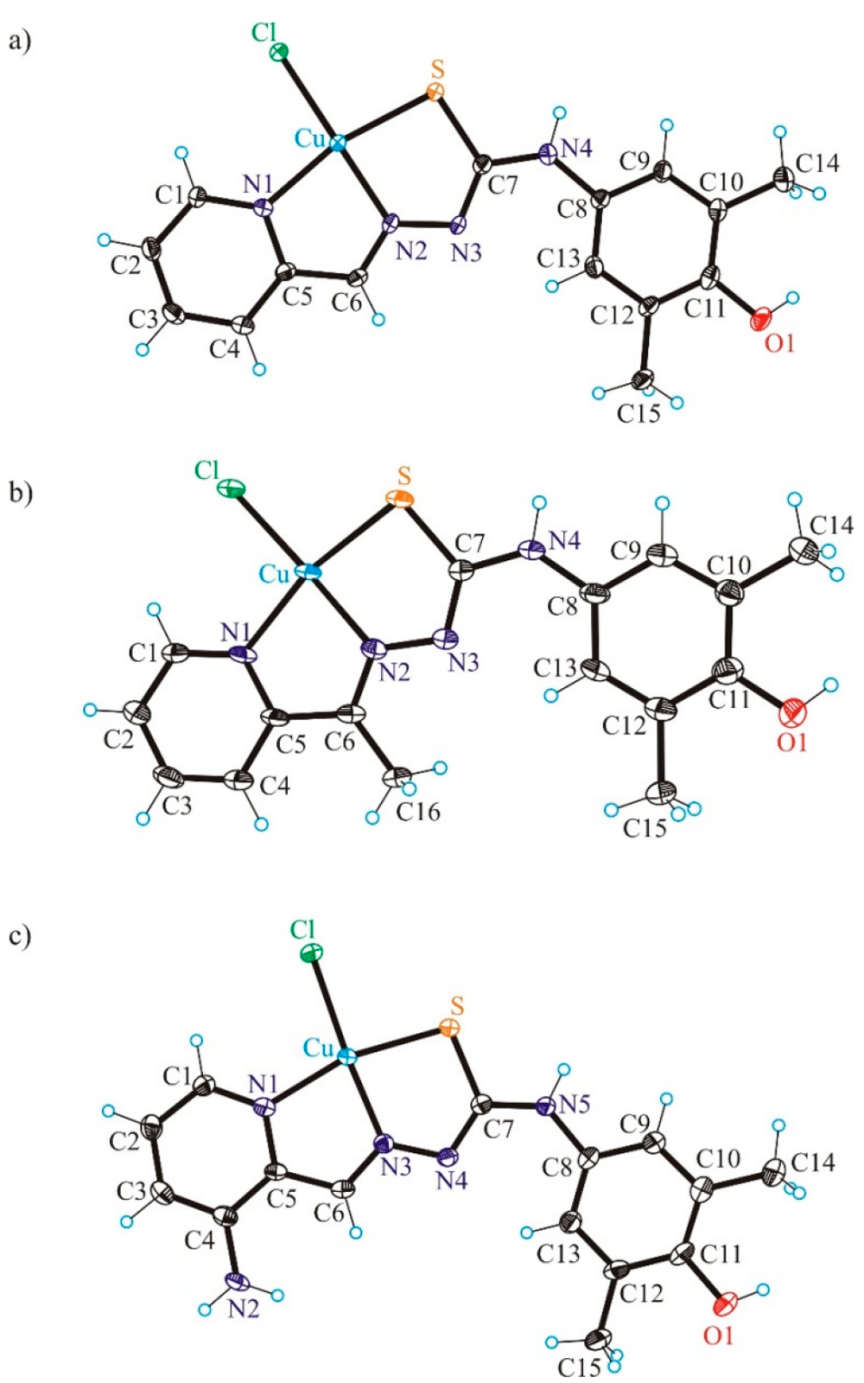
ORTEP views of **1′**–**3′** with thermal ellipsoids at the 50% probability level. Selected bond distances (Å), bond angles (deg) and torsion angles (deg) in **1′**: Cu–N1 2.005(2), Cu–N2 1.962(2), Cu–S 2.2325(7), Cu–Cl 2.2507(7), C11–O1 1.370(4); N1–Cu–N2 81.77(9), N2–Cu–S 84.07(7), Θ_C7–N4–C8–C13_ −0.8(5); in **2′**: Cu–N1 2.022(4), Cu–N2 1.952(4), Cu–S 2.2636(16), Cu–Cl 2.2215(15), C11–O1 1.370(6); N1–Cu–N2 80.76(17), N2–Cu–S 84.46(12), Θ_C7–N4–C8–C13_ −2.1(8); in **3′**: Cu–N1 2.025(2), Cu–N3 1.961(2), Cu–S 2.2432(8), Cu–Cl 2.2636(8), C11–O1 1.374(4); N1–Cu–N3 81.58(10), N3–Cu–S 83.40(7), Θ_C7–N5–C8–C13_ 9.4(5).

The results of X-ray diffraction studies of TSCs **HL**^**1**^**·C**_**2**_**H**_**5**_**OH**, **HL**^**2**^ and **HL**^**3**^ are presented in Figure 3, while those of **[Cu(L**^**1**^**)Cl]·CH**_**3**_**OH** (**1′·CH**_**3**_**OH**), **[Cu(L**^**2**^**)Cl]** (**2′**), and **[Cu(L**^**3**^**)Cl]·CH**_**3**_**OH** (**3′·CH**_**3**_**OH**) are in Figure 4. The **HL**^**1**^**·C**_**2**_**H**_**5**_**OH** crystallized in the triclinic centrosymmetric space group *P*1̅, while **HL**^**2**^ and **HL**^**3**^ crystallized in the monoclinic space groups *P*2_1_/*c* and *P*2_1_/*n*, respectively. All three metal-free ligands adopt an *E* configuration in terms of the nomenclature used for the α-*N*-heterocyclic thiosemicarbazones^41^ with the imine nitrogen in the *s-trans* position to the sulfur atom and the pyridine N1 atom. All TSCs crystallized in the thione form with the C7–S bond length of 1.6839(15), 1.683(4) and 1.695(2) Å, respectively. The distribution of electron density in the dimethylphenolic moiety is typical for aromatic systems. The C11–O bond length of 1.3780(19), 1.370(4), and 1.380(2) Å, respectively, is also characteristic for phenols. The molecules of the three proligands are not planar. The strong deviation of the phenolic unit from the mean plane of the thiosemicarbazone fragment can be estimated by a comparison of the torsion angle Θ_C7–N4–C8–C13_ of 88.7(2) and 78.4(4)° in the first two structures (Figure 3a,b) and Θ_C7–N5–C8–C13_ and Θ_C22–N10–C23–C28_ of 52.5(3) and 54.2(3)° in two crystallographically independent molecules of **HL**^**3**^ (Figure 3c).

In contrast to the structures of **HL**^**1**^ and **HL**^**2**^, the asymmetric unit of **HL**^**3**^ consists of two molecules associated in a centrosymmetric dimer via hydrogen-bonding interactions, namely, N4–H···S2 [N4–H4 = 0.88 Å, H4···S2 = 2.48 Å, N4···S2 = 3.3243(17) Å] and N9–H···S1 [N9–H9 = 0.88°, H9···S1 = 2.47 Å, N9···S1 = 3.3341(17) Å]. A similar centrosymmetric association was recently reported for acetylpyrazine 4-*N*-phenyl thiosemicarbazone.^54^

The copper(II) complexes **1′·CH**_**3**_**OH** and **3′·CH**_**3**_**OH** crystallized in the monoclinic centrosymmetric space group *P*2_1_/*c*, while **2′** crystallized in the triclinic centrosymmetric space group *P*1̅ without any cocrystallized solvent. The copper(II) adopts a square-planar coordination geometry in all three structures (Figure 4). The thiosemicarbazones act as tridentate monoanionic ligands binding to copper(II) via a pyridine nitrogen atom, an azomethine nitrogen atom, and a thiolate sulfur atom. The fourth coordination site in all complexes is occupied by the chlorido coligand. Pertinent bond distances and bond angles are quoted in the legend to Figure 4. The same coordination geometry of a copper(II) bound by a monoanionic thiosemicarbazone and a monodentate coligand was reported for [CuCl(mPip-FTSC–H)]·0.15CH_3_OH,^55^ [Cu(L_1_)(μ-Cl)]Cl, and [Cu(L_2_)(μ-Cl)]Cl·H_2_O, where ligands L_1_ and L_2_ represent 3-methyl-5-oxo-1-phenyl-3-pyrazolin-4-carboxaldehyde and 5-oxo-3-phenyl-3-pyrazolin-4-carboxaldehyde TSC, respectively.^56^

A comparison of the Cu(II) to TSC ligand bond lengths in **1′** with those in the copper(II) complex with pyridine-2-carboxaldehyde thiosemicarbazone^57^ (Cu–N1 = 2.034(4), Cu–N2 = 1.975(3), Cu–S = 2.278(1) Å) shows that these are statistically significantly shorter in **1′**. This difference is probably due to the formation of centrosymmetric associates via intermolecular interactions with the shortest contact Cu···S^i^ = 2.760(2) Å and not due to the presence of a phenolic moiety at N4. The interatomic repulsions in the copper(II) complex with a 4 + 1 coordination geometry are expected to be stronger than those in **1′**, in which the Cu(II) is four-coordinate. In another complex [CuLCl]_2_[Cu(pic)_2_] (with HL = pyridine-2-carboxaldehyde thiosemicarbazone and pic^–^ = pyridine-2-carboxylate), in which the intermolecular contacts are over 3 Å, the Cu(II) to TSC bond distances are shorter and very similar to those in **1′** (Cu–N1 = 2.005(8), Cu–N2 = 1.942(9), Cu–S = 2.264(3) Å).^48^ The metric parameters in the copper(II)-ligand chromophore of [Cu(triapine-H)Cl](H_3_O)Cl (Cu–N_py_ = 2.031(8), Cu–N_hydrazine_ = 1.937(9), Cu–S = 2.281(3) and Cu–Cl = 2.2493(5) Å) are statistically the same as those in **3′**, except Cu–S, which is by ca. 0.04 Å (>12σ) shorter in **3′** than in the copper(II) complex with triapine. This is likely due to different protonation states of the ligands in the two complexes, even though the authors described the triapine ligand in its copper(II) complex as a monoanion with an extra proton at a cocrystallized water molecule.^58^

Note that the organic ligands in all three complexes are almost planar in contrast to the situation described previously for the metal-free ligands. The value of the torsion angle Θ_C7–N4–C8–C13_ for **1′·CH**_**3**_**OH** and **2′** (Figure 4a,b) increased from −88.7(2) and −78.4(4)° in **HL**^**1**^ and **HL**^**2**^ to −0.8(5) and −2.1(8)°, respectively. Analogously, the torsion angle Θ_C7–N5–C8–C13_ of 52.5(3) in **HL**^**3**^ becomes of 9.4(5)° in **3′·CH**_**3**_**OH** upon coordination to copper(II).

As for the metal-free TSCs, the phenolic moiety remained intact in all three complexes, namely, in its original oxidation state. The distribution of electron density over the aromatic phenolic ring is well-comparable to that in the TSCs.

### X-ray Crystallography of Oxidized Products

**Figure 5:**
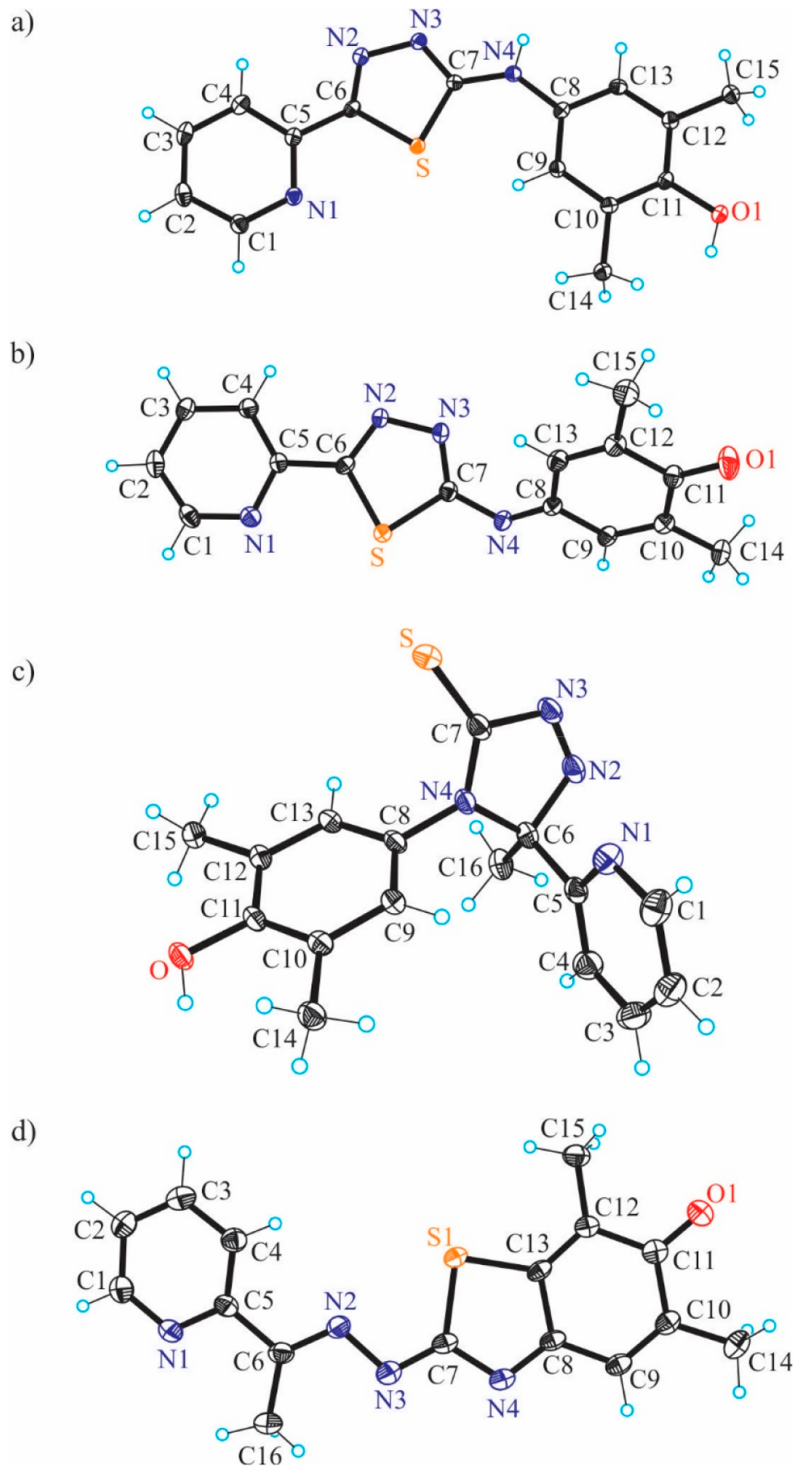
ORTEP views of two-electron and four-electron oxidized species of (a) **H*L***^**1a′**^ and (b) **H*L***^**1a″**^, as well as of products that resulted from an oxidation of **HL**^**2**^, namely, of (c) **H*L***^**2b**^ and (d) **H*L***^**2c″**^**·0**.**5CHCl**_**3**_. Selected bond distances (Å) and torsion angles (deg) in (a) **H*L***^**1a′**^: C6–N2 1.3029(17), N2–N3 1.3739(15), C6–S 1.7405(14), C7–S 1.7382(13), C8–C9 1.3878(19), C9–C10 1.4003(18), C10–C11 1.3927(19), C11–C12 1.4006(19), C12–C13 1.3922(19), C11–O1 1.3820(16); Θ_C7–N4–C8–C9_ 1.0(2); in (b) **H*L***^**1a″**^: C6–N2 1.305(3), N2–N3 1.382(2), C6–S 1.727(2), C7–S 1.734(2), C8–C9 1.458(3), C9–C10 1.341(3), C10–C11 1.480(3), C11–C12 1.491(3), C12–C13 1.342(3), C11–O1 1.226(3); Θ_C7–N4–C8–C13_ 5.8(3); in (c) **H*L***^**2b**^: C6–N2 1.485(2), N2–N3 1.247(2), N3–C7 1.472(2), C7–S 1.6465(18), C7–N4 1.325(2), N4–C6 1.479(2), C11–O 1.3728(18), N4–C8 1.4409(19); in (d) **H*L***^**2c″**^**·0**.**5CHCl**_**3**_: C6–N2 1.306(4), N2–N3 1.394(4), N3–C7 1.296(4), C7–N4 1.388(4), N4–C8 1.311(4), C8–C9 1.444(4), C9–C10 1.340(4), C10–C11 1.488(5), C11–O1 1.234(4), C11–C12 1.496(4), C12–C13 1.349(4), C13–C8 1.461(4), C13–S1 1.745(3).

**Figure 6:**
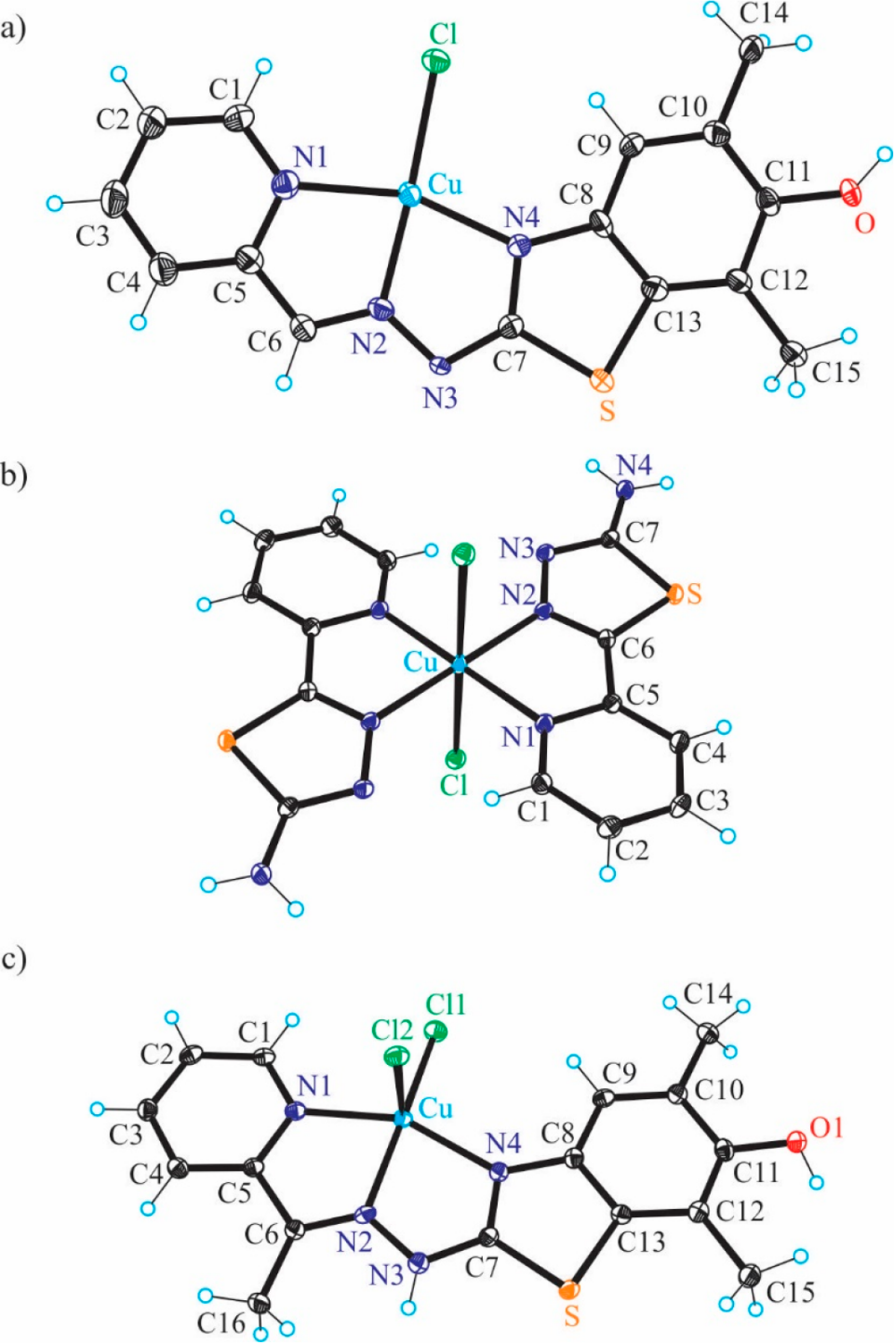
ORTEP views of **[Cu(*L***^**1c′**^**)Cl]** (**4**), **[Cu(H*L***^**1d**^**)**_**2**_**Cl**_**2**_**]** (**5**), and **[Cu(H*L***^**2c′**^**)Cl**_**2**_**]** (**6**) with thermal ellipsoids at the 50% probability level. Selected bond distances (Å) and bond angles (deg) in (a) **4**: Cu–N1 2.054(4), Cu–N2 1.956(4), Cu–N4 2.001(3), Cu–Cl 2.2575(12), C11–O 1.370(5); N2–Cu–N1 80.02(15), N2–Cu–N4 78.59(14); in (b) **5**: Cu–N1 2.0384(11), Cu–N2 2.0089(11), Cu–Cl 2.8116(3), N2–Cu–N1 99.32(4); in (c) **6**: Cu–N1 2.0329(16), Cu–N2 1.9854(16), Cu–N4 2.0358(16), Cu–Cl1 2.2100(5), C11–O 1.374(2); N2–Cu–N1 78.00(16), N2–Cu–N4 79.31(6).

The results of X-ray diffraction studies of oxidized organic species **H*L***^**1a′**^, **H*L***^**1a″**^, **H*L***^**2b**^, **H*L***^**2e**^, and **H*L***^**2c″**^**·0**.**5CHCl**_**3**_ are displayed in Figure 5 and Figure S6, while those of copper(II) complexes with oxidized ligands **4**–**6** are shown in Figure 6 and Figure S7. The oxidized species **H*L***^**1a′**^ and **H*L***^**1a″**^ crystallize in the monoclinic space groups *P*2_1_/*n* and *Cc*, respectively. The molecule **H*L***^**1a′**^ is almost planar, while in **H*L***^**1a″**^ the moiety at N4 slightly deviates from planarity. The dihedral angle Θ_C7–N4–C8–C13_ is of 5.8(3)°. Both contain a thiadiazole five-membered ring. The distribution of electron density in them is very similar. In contrast, the bond length distribution in the aryloxide moiety is quite different. In the two-electron oxidized product **H*L***^**1a′**^ the distribution of electron density is in agreement with that of the 3,5-dimethyl-1,4-aminophenolic moiety, while in the four-electron oxidized species **H*L***^**1a″**^ the electron density agrees with that of the 3,5-dimethyl-1,4-benzoquinone imine unit (see legend to Figure 5a,b). In particular, the C11–O1 bond length in these two compounds is quite different at 1.3820(16) and 1.226(3) Å, respectively. The X-ray diffraction structure of **H*L***^**2b**^ confirmed the two-electron oxidation of the original ligand **HL**^**2**^ and the formation of the TAT ring, while that of **H*L***^**2c″**^ confirmed the further two-electron oxidation of **H*L***^**2c′**^. The bond-length distribution in the molecule of **H*L***^**2c″**^ indicates the presence of the benzo[d]thiazol-6-one moiety. The double-bond character of N3–C7 1.296(4) indicates the formation of this four-electron oxidation product from the two-electron oxidation product **H*L***^**2c′**^ by the loss of two electrons and two protons.

The X-ray diffraction study of **4** (Figure 6a) revealed that the ligand underwent an oxidative dehydrogenation accompanied by the intramolecular cyclization via a C–S coupling reaction between a phenolic carbon and a thione group into a five-membered thiazole ring instead of the expected oxidative dehydrogenation (two-electron oxidation accompanied by the loss of two protons) of the 3,5-dimethyl-1,4-aminophenol unit with formation of a 3,5-dimethyl-1,4-benzoquinone imine moiety (see Chart 2, Scheme 1). This intramolecular sulfur arylation resulted in the change of coordination mode, so that the thioether sulfur atom with diminished electron-donor properties is not involved in the coordination to copper(II). This is in agreement with the coordination chemistry of isothiosemicarbazones,^59^ which as a rule do not use a sulfur atom for coordination to first-row transition metals. In this context, it is worth mentioning that the binding of isothiosemicarbazones to zinc(II) and copper(II) via a thioether sulfur atom has been documented quite recently,^60^ when bulkier than chlorido coligands, for example, iodido and bromido, were involved in coordination to the metal. Complex **4** might be one of the products of the oxidation of copper(II) complexes over time in methanol by air oxygen. Some rare examples of a thiosemicarbazone cyclization with the thiazole ring formation due to the coordination to copper(II) were recently reported (iminodiacetate–thiosemicarbazones and *N*-phenylthiosemicarbazones).^52,53,61^ The new ligand obtained by the intramolecular cyclization in **Cu(HL**^**1**^**)Cl**_**2**_ belongs to the class of biologically active substituted 2-hydrazinylbenzothiazoles, which showed anticancer activity themselves as well as upon coordination to different metals.^62−65^ Two molecules of complex **4** are associated into a centrosymmetric dimer via two intermolecular μ-chlorido bridges as shown in Figure S7.

The molecular structure of **5** shown in Figure 6b indicates a strongly tetragonally distorted six-coordinate geometry of copper(II), in which two pyridine-thiadiazole ligands act as bidentate and occupy the equatorial sites in a *trans* mutual arrangement and two quite weakly bound chlorido coligands in axial positions. Taking into account the interatomic Cu–Cl separation (2.8116(3) Å) the complex can also be described as square-planar.

As in **4**, the coordinated ligand in **6** acts as tridentate and binds to copper(II) via atoms N1, N2, and N4. However, while **4** is square-planar, **6** is very close to square-pyramidal (τ_5_ = 0.16).^66^ The organic ligand is monoanionic in **4**, while neutral in **6**. An additional coordination of chlorido coligands counterbalances the 2+ charge of the central atom.

To understand the difference in protonation states and reactivity of the originally prepared complexes and those isolated upon crystallization from diluted methanolic solutions equilibrium studies were performed on the ligands and their copper(II) complexes.

## Solution Equilibrium Studies

### Proton Dissociation Processes and Lipophilicity of the Ligands

**Figure 7:**
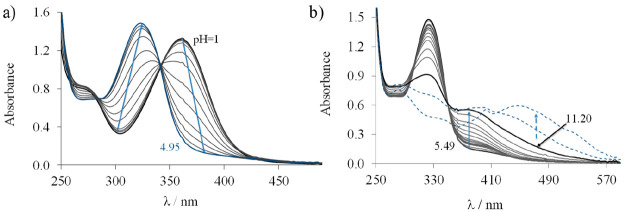
UV–vis absorption spectra recorded for proligand **HL**^**1**^ in the pH ranges of (a) 1.00–4.95 and (b) 5.49–11.82. *c*_HL_ = 50 μM; 30% (v/v) DMSO/H_2_O; *I* = 0.1 M (KCl); *T* = 25 °C.

Proton dissociation constants (p*K*_a_) of drug molecules indicate the actual protonation state and the charge at a given pH, and therefore p*K*_a_ are important parameters that affect the pharmacokinetic properties as well. The N-terminally monosubstituted TSCs **HL**^**1**^–**HL**^**3**^ belong to the family of α-*N*-pyridyl TSCs; thus, they possess the pyridinium (PyH)^+^ and the hydrazinic-NNH as proton dissociable groups besides the phenolic moiety. Since these TSCs and their copper(II) complexes have a limited water solubility, the equilibrium studies were performed by UV–vis spectrophotometry in a 30% (v/v) DMSO/H_2_O solvent mixture using relatively low concentrations (50 μM). Representative UV–vis spectra recorded for **HL**^**1**^ at various pH values are shown in Figure 7a.

On the basis of the spectral changes two well-separated deprotonation processes were observed between pH 2 and 11. The first proton dissociation step taking place at pH < 5 is accompanied by a blue shift, and the λ_max_ is shifted from 362 to 322 nm. This deprotonation step is attributed to the proton on the pyridinium nitrogen (PyH^+^). Upon an increase of the pH a new process occurred as evidenced by a new band in the range of 350–450 nm (Figure 7b) and an isosbestic point at 350 nm, namely, the deprotonation of the hydrazinic nitrogen. In the strongly basic pH range (pH > 11.2) new broad bands appear at 400–600 nm (Figure 7b) with irreversible spectral changes most likely due to an oxidation of the TSC by the air oxygen.

Therefore, only two p*K*_a_ values could be determined (Table 1) based on the deconvolution of the UV–vis spectra recorded at pH < 11.2 for **HL**^**1**^ (molar absorbance spectra are seen in Figure S8a) as the oxidation hindered the accurate determination of the p*K*_a_ for the aromatic OH group. Two p*K*_a_ values were computed for **HL**^**2**^ from the UV–vis titration data (Figure S9) as well; however, only one p*K*_a_ was obtained in the case of **HL**^**3**^ (Table 1), namely, that for the deprotonation of the PyH^+^, since the proton dissociation of the hydrazinic nitrogen and the oxidation of the TSC were partly overlapped. On the basis of the determined p*K*_a_ values, it can be concluded that the presence of the electron-donating methyl group in **HL**^**2**^ results in a significant increase of both p*K*_a_ values when compared to that of **HL**^**1**^. A similar behavior was reported for the analogous 2-formylpyridine and 2-acetylpyridine TSC in our previous work.^67^ The p*K*_a_ of the PyH^+^ group was also increased significantly by the addition of the electron-donating amine group at the pyridine ring, in agreement with data reported previously for the FTSC and triapine.^68^ All proligands are air-sensitive in the strongly basic pH range (pH > 11). Concentration distribution curves were computed for them at pH < 11 (see Figure S8b for **HL**^**1**^) revealing that their neutral forms predominate at a physiological pH.

**Table 1:** p*K*_a_ Values Determined by UV–vis Titrations in 30% (v/v) DMSO/H_2_O and log *D*_7.4_ (*n-*Octanol/Water) Values of the TSCs **HL**^**1**^–**HL**^**3**^ and Their Complexes^a^

	method	HL^1^	HL^2^	HL^3^
p*K*_a_ (PyH^+^)	UV–vis	3.01 ± 0.01	3.59 ± 0.02	3.95 ± 0.04
p*K*_a_ (NNH)	UV–vis	10.55 ± 0.01	11.08 ± 0.02	nd
log *D*_7.4_ (proligand)	partitioning	+1.30 ± 0.03	+2.1 ± 0.1	+1.67 ± 0.01
log *K*′_5.9_ (complex)	EDTA displacement	9.67 ± 0.01	nd^b^	9.78 ± 0.01
log *D*_7.4_ (complex)	partitioning	–0.40 ± 0.06	nd^b^	–0.42 ± 0.03
*k*_obs_ (min^–1^) (complex) in 30% DMSO	UV–vis (with GSH)	0.033 ± 0.004	nd^b^	0.035 ± 0.004
*k*_obs_ (min^–1^) (complex) in 60% DMSO	UV–vis (with GSH)	0.021 ± 0.001	too slow^c^	0.024 ± 0.004

a Conditional stability constants (log *K*′_5.9_) of the complexes determined by UV–vis EDTA displacement studies in 30% (v/v) DMSO/H_2_O and rate constants (*k*_obs_) obtained for the redox reaction of the complexes with GSH (pH = 7.4 (50 mM HEPES); *c*_complex_ = 25 μM; *c*_GSH_ = 1.25 mM in 30% (v/v) DMSO/H_2_O); *c*_complex_ = 12.5 μM; *c*_GSH_ = 600 μM in 60% (v/v) DMSO/H_2_O) {*T* = 25 °C; *I* = 0.1 M (KCl)}.

b Not determined (nd) due to the bad solubility of the complex under the conditions.

c Rate constant could not be determined due to the very slow redox reaction.

The solution stability of the proligands was monitored at pH 7.4 by spectrophotometry. The UV–vis spectra recorded over 4 h revealed no measurable spectral changes, suggesting that the oxidation of these proligands does not take place (or just very slowly) in an aqueous solution at a physiological pH. However, **HL**^**2**^ showed a certain level of slow decomposition at pH 1.5, namely, a 6% absorbance decrease at 354 nm in ∼3 h (Figure S10), which is most likely the consequence of the less extended conjugation in the molecule due to the cleavage of the C=N Schiff base bond, as it was also reported for 2-acetylpyridine TSC.^67^ Thus, the rate of this acid-catalyzed reaction is increased with the increasing number of methyl groups present in the α-*N*-pyridyl TSC.

Besides p*K*_a_ values, lipophilicity is also an important pharmacological property of a drug, as it strongly influences the ability of the compound to pass through biological membranes. Therefore, distribution coefficients (log *D*_7.4_) were determined using the shake-flask method in an *n*-octanol-buffered aqueous solution at pH 7.4 (Table 1). The log *D*_7.4_ values indicate the moderate lipophilic character of the proligands. The substitution at the end nitrogen atom of the thosemicarbazide moiety and the presence of a methyl group at the Schiff base bond induce a somewhat higher lipophilicity. The presence of the phenolic moiety undoubtedly increases the log *D*_7.4_ values compared to those of FTSC (+0.73),^67^ AcTSC (+1.02)^67^ and triapine (+0.85).^69^

In summary, these TSCs are stable in their neutral form in a quite broad pH range (including pH 7.4).

### Solution Stability and Redox Properties of the Copper(II) Complexes

**Figure 8:**
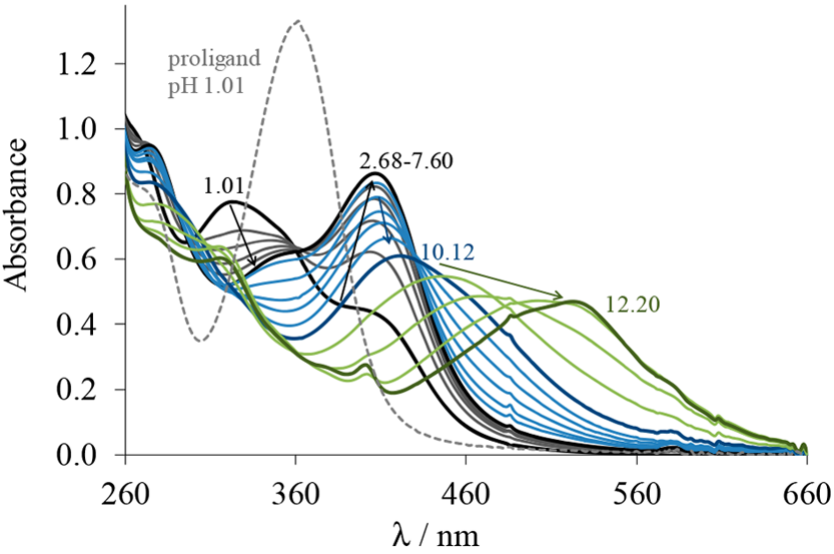
UV–vis absorption spectra recorded for complex **Cu(HL**^**1**^**)Cl**_**2**_ in the pH range of 1.01–12.20 (solid lines) and for **HL**^**1**^ at pH 1.01 (dashed gray line). *c*_complex/HL_ = 50 μM; 30% (v/v) DMSO/H_2_O; *I* = 0.1 M (KCl); *T* = 25 °C.

The metal complexes often undergo transformation processes upon dissolution, such as protonation, deprotonation, or dissociation to a metal-free ligand and metal ion depending on the pH, their concentration, and the solution speciation. The knowledge of the actual chemical form of the biologically active metal complexes in solution close to physiologically relevant conditions is quite important to elucidate the mechanism of action. Therefore, the solution stability of the copper(II) complexes (**Cu(HL**^**1**^**)Cl**_**2**_, **[Cu(L**^**2**^**)Cl]**, and **Cu(HL**^**3**^**)Cl**_**2**_) was studied by UV–vis spectrophotometry. The simple α-*N*-pyridyl TSCs (e.g., triapine, FTSC) generally form very stable monoligand copper(II) complexes, and the species in which the monoanionic ligand is coordinated via the (N_pyridine_,N,S^–^) mode predominates in a wide pH range at a 1:1 metal-to-ligand ratio.^68^ At lower pH this type of complex is protonated, and thus the neutral ligand is bound via (N_pyridine_,N,S) donor atoms, while a mixed hydroxido complex with the (N_pyridine_,N,S^–^)(OH) coordination pattern is formed in the basic pH range. On the basis of the close structural similarities between **HL**^**1**^–**HL**^**3**^ and the listed TSCs with a simpler scaffold, the formation of the same type of complexes is feasible. UV–vis titrations were performed with the complexes in a 30% (v/v) DMSO/H_2_O solvent mixture, and representative spectra are shown for **Cu(HL**^**1**^**)Cl**_**2**_ in Figure 8. The spectra remain intact in a broad pH range (2.7–7.6), and an absorption band is observed with λ_max_ at 406 nm being typical for a S → Cu charge transfer. This finding indicates the dominant presence of only one kind of complex, which is most probably the species with the (N_pyridine_,N,S^–^) tridentate coordination mode. By decreasing the pH the λ_max_ is hypsochromically shifted to 322 nm. The presence of the isosbestic point at 362 nm implies that only two species are involved in this equilibrium. As the spectrum recorded at pH 1.01 significantly differs from that of the TSC, this equilibrium corresponds to the protonation of the complex at the noncoordinating hydrazinic nitrogen (Chart S1) rather than to its dissociation to the free metal ion and ligand. This process is not completed when the pH decreases to 1, and a p*K*_a_ value less than 1.5 could be estimated. When the pH is increased, two overlapping processes are suggested to take place at pH > 8 via the continuous bathochromic shift of the absorption maximum, and p*K*_a_ values of 9.80 ± 0.01 and 11.02 ± 0.01 were computed. In this pH range most probably the coordinated water molecule deprotonates, and a mixed hydroxido complex is formed along with the deprotonation of the phenolic group of the bound ligand. Similar spectral changes were monitored for **Cu(HL**^**3**^**)Cl**_**2**_, and p*K*_a_ < 1.5 was estimated for the process in the acidic pH range as well.

However, the formation of precipitate (significant baseline elevation and absorbance decrease in the whole wavelength range) at pH > 8 hindered the calculation of the proton dissociation constants of the complexes from spectra collected in this pH range. Unfortunately, during the titration of **[Cu(L**^**2**^**)Cl]** the formation of a precipitate was observed already at the acidic pH; thus, the deprotonation processes could not be evaluated.

**Figure 9:**
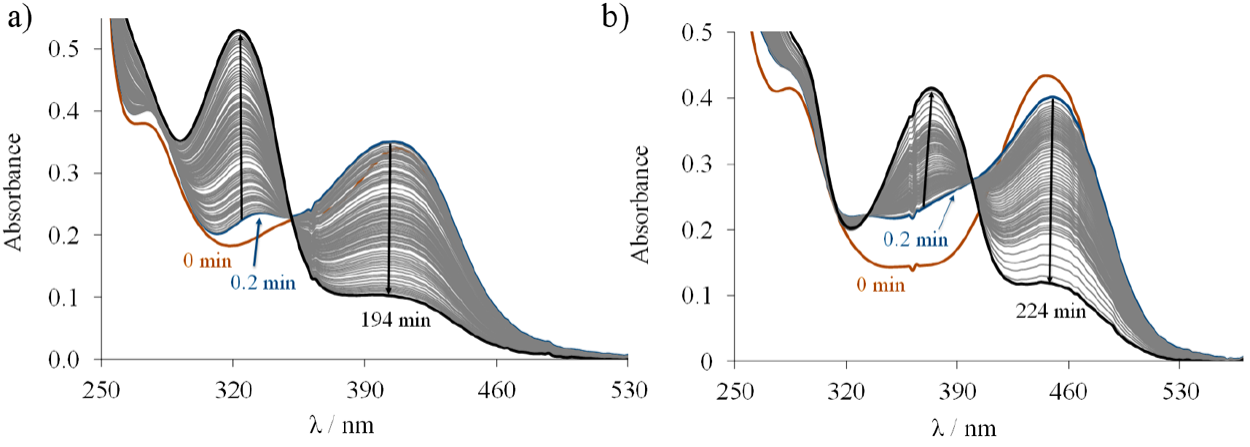
Time-dependent changes of the UV–vis spectra of (a) **Cu(HL**^**1**^**)Cl**_**2**_ and (b) **Cu(HL**^**3**^**)Cl**_**2**_ in the presence of 50 equiv of GSH at pH 7.4 under anaerobic conditions. *c*_complex_ = 25 μM; *c*_GSH_ = 1.25 mM; pH = 7.40; 30% (v/v) DMSO/H_2_O; *I* = 0.1 M (KCl); *T* = 25 °C.

The copper(II)–TSC complexes are often redox-active under physiological conditions, which has an impact on their cytotoxicity. To investigate whether complexes **[Cu(L**^**1**^**)]**^**+**^, **[Cu(L**^**2**^**)]**^**+**^, and **[Cu(L**^**3**^**)]**^**+**^ can be reduced by the most abundant low molecular mass cellular reductant, GSH, spectrophotometric measurements were performed on their direct reaction under strictly anaerobic conditions at pH 7.4. First, the assay was performed in the presence of 30% DMSO using a 25 μM complex concentration. However, the limited solubility of **[Cu(L**^**2**^**)]**^**+**^ did not allow the measurement. Therefore, the assay was also performed in the presence of 60% DMSO at a lower (12.5 μM) concentration for all the three complexes. The spectral changes are shown in Figure 9 for **[Cu(L**^**1**^**)]**^**+**^ and **[Cu(L**^**3**^**)]**^**+**^ complexes in the presence of a large excess of GSH in 30% (v/v) DMSO/H_2_O. After the complexes were mixed with GSH, a well-detectable change is observed due to the formation of ternary complexes via the coordination of GSH as it was reported for several TSC complexes.^70,71^ Then the spectral changes show the absorbance decrease at the λ_max_ of the S → Cu charge transfer band of the complexes. The final spectra show a strong similarity to those of **HL**^**1**^ and **HL**^**3**^ at λ > 310 nm suggesting the release of the TSCs. However, in this case the reduction is responsible for the liberation of the TSCs and copper(I), which forms complexes with GSH (that is in high excess in the sample). Copper(I) favors a tetrahedral coordination environment, while **HL**^**1**^ and **HL**^**3**^ as planar tridentate ligands cannot satisfy these requirements and accommodate the cation. This contradiction is a driving force for a complex destabilization, especially in the presence of GSH, which can efficiently bind copper(I).^64^ In addition, a one-electron reduction increases the basicity of the coordinated TSCs facilitating their protonation and dissociation from the copper(I).^72^ Note, however, that the process was reversible, as bubbling oxygen into the samples regenerated the starting spectra. Complex **[Cu(L**^**2**^**)]**^**+**^ behaved differently, as only minor spectral changes were seen upon treatment with GSH in 60% (v/v) DMSO/H_2_O (Figure S11b). From the measured absorbance–time curves rate constants (*k*_obs_) were calculated (Table 1). Similar reduction rates for **[Cu(L**^**1**^**)]**^**+**^ and **[Cu(L**^**3**^**)]**^**+**^ complexes were obtained, and somewhat lower *k*_obs_ values were found in the presence of the higher fraction of DMSO. Notably, ascorbate, which is a weaker reducing agent compared to GSH and is found in higher concentration in the extracellular fluids, was not able to reduce these complexes under the same conditions. On the contrary, the more powerful reducing agent DTT could reduce **[Cu(L**^**1**^**)]**^**+**^, **[Cu(L**^**2**^**)]**^**+**^, and **[Cu(L**^**3**^**)]**^**+**^ in a very fast reaction. The reduction was complete within several seconds (at 12.5 μM complex and 600 μM DTT concentrations in the presence of 60% DMSO, Figure S11c,d). In this case, the reaction was reversible upon exposure to O_2_ only for **[Cu(L**^**2**^**)]**^**+**^.

Overall, the solution equilibrium data provide further evidence that the complex **[Cu(L)]**^**+**^ with the coordinated monoanionic ligand predominates in a wide pH range. In order to obtain a deeper insight into the observed behavior of both metal-free ligands and their copper(II) complexes in the presence of oxidants (atmospheric oxygen) and reductants (GSH and ascorbate) spectroelectrochemical investigations were also performed.

### Electrochemistry and Spectroelectrochemistry

**Figure 10:**
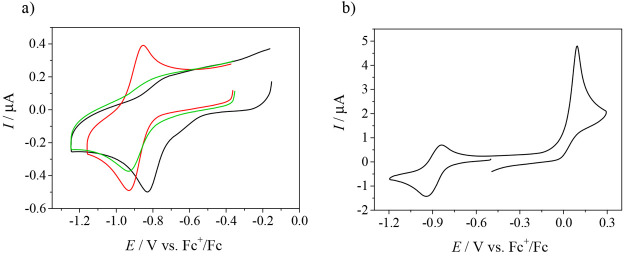
(a) Cyclic voltammograms of 0.5 mM **1** (black trace), **2′** (red trace), and **3** (green trace) in DMSO/*n-*Bu_4_NPF_6_ at a GC working electrode at the scan rate of 100 mV s^–1^; (b) comparison of the reduction and the oxidation peak of **2′** (scan rate of 100 mV s^–1^).

Cyclic voltammograms of **1**, **2′**, and **3** in DMSO/*n-*Bu_4_NPF_6_ recorded with a glassy carbon (GC) working electrode at a scan rate of 100 mV s^–1^ showed a redox activity in both cathodic and anodic regions. Copper(II) undergoes an electrochemically irreversible or quasi-reversible reduction to copper(I) at *E*_pc_ = −0.83 V for **1** and −0.93 V versus Fc^+^/Fc for both **2′** and **3** (Figure 10a). Notably, the corresponding ligands are not redox-active in the cathodic region (data not shown). An irreversible oxidation was observed for these complexes, which was identified as a two-electron oxidation of the TSCs with a release of two protons. A two-electron oxidation was confirmed by a comparison of the reduction peak (one-electron Cu(II) → Cu(I) redox process) and the oxidation peak of **2′** taken in equivalent amounts as shown in Figure 10b. In addition, an electrolysis of **HL**^**1**^ at 1000 mV versus Ag/AgCl in CH_3_CN in the presence of 0.2 M *n*-Bu_4_NPF_6_ generated a mixture of several products from which **H*L***^**1a′**^ and **H*L***^**1a″**^ were separated on silica. ESI-MS and ^1^H NMR spectra were identical with those of the products obtained by an oxidation of **HL**^**1**^ with DDQ as mentioned previously.

**Figure 11:**
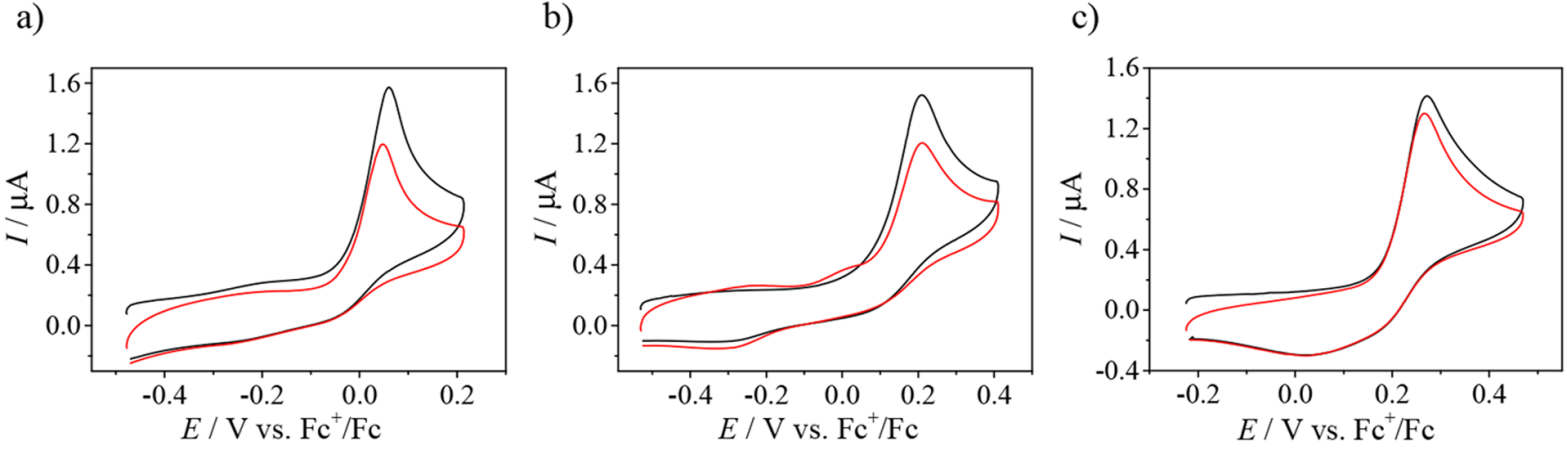
Cyclic voltammograms of 0.5 mM of (a) **1** and (b) the corresponding ligand in DMSO/*n-*Bu_4_NPF_6_ and of (c) **1** in MeOH/LiClO_4_ at the GC working electrode, at scan rate of 100 mV s^–1^.

The oxidation peak of the TSC ligand was observed at *E*_pa_ = +0.06 V for **1** and **2′** and at +0.04 V for **3**, and it is negatively shifted in comparison to the corresponding metal-free ligands (*E*_pa_ = +0.21 V for **HL**^**1**^, +0.24 V for **HL**^**2**^, and +0.18 V for **HL**^**3**^ (all vs Fc^+^/Fc at a scan rate of 100 mV s^–1^)), as shown for **1** and its corresponding metal-free ligand **HL**^**1**^ in Figure 11a,b, respectively. There are also significant changes in the shape and intensity of cyclic voltammograms upon the second oxidation scan (see red traces in Figure 11a,b), which indicate a further oxidation of the products obtained after the first oxidation in DMSO, in line with the chemical oxidation of the compounds. Note that, in a proton-donating solvent, the potentials of both reduction and oxidation processes were shifted to the more positive values versus the internal potential standard Fc^+^/Fc, and additionally, a broad reduction peak appeared during the reverse scan in the cyclic voltammogram at a strongly negatively shifted potential (Figure 11c). A distinct oxidation pattern of the corresponding voltammograms in protic media is caused by the involvement of protons in the process in accordance with chemical oxidations discussed previously and the well-known reaction mechanism proposed for the quinone-like systems.^70,71^

Similar redox behavior was observed for the anodic oxidation of **H*L***^**1a′**^ in DMSO with several new redox-active species, which appeared upon the first and the second voltametric scans (Figure S12a). However, the oxidized 1,4-benzoquinone imine species **H*L***^**1a″**^can be reversibly reduced in the cathodic part (Figure S12b) with a voltammetric pattern characteristic for the electrochemistry of quinones in aprotic media.^72^ Moreover, EPR spectroelectrochemistry confirmed the formation of an anion radical at the first reduction peak (see inset in Figure S12b). A rich hyperfine splitting and a *g*-value of 2.0046 points to the spin delocalization and contribution of heteroatom (presumably nitrogen) to the *g*-value.

**Figure 12:**
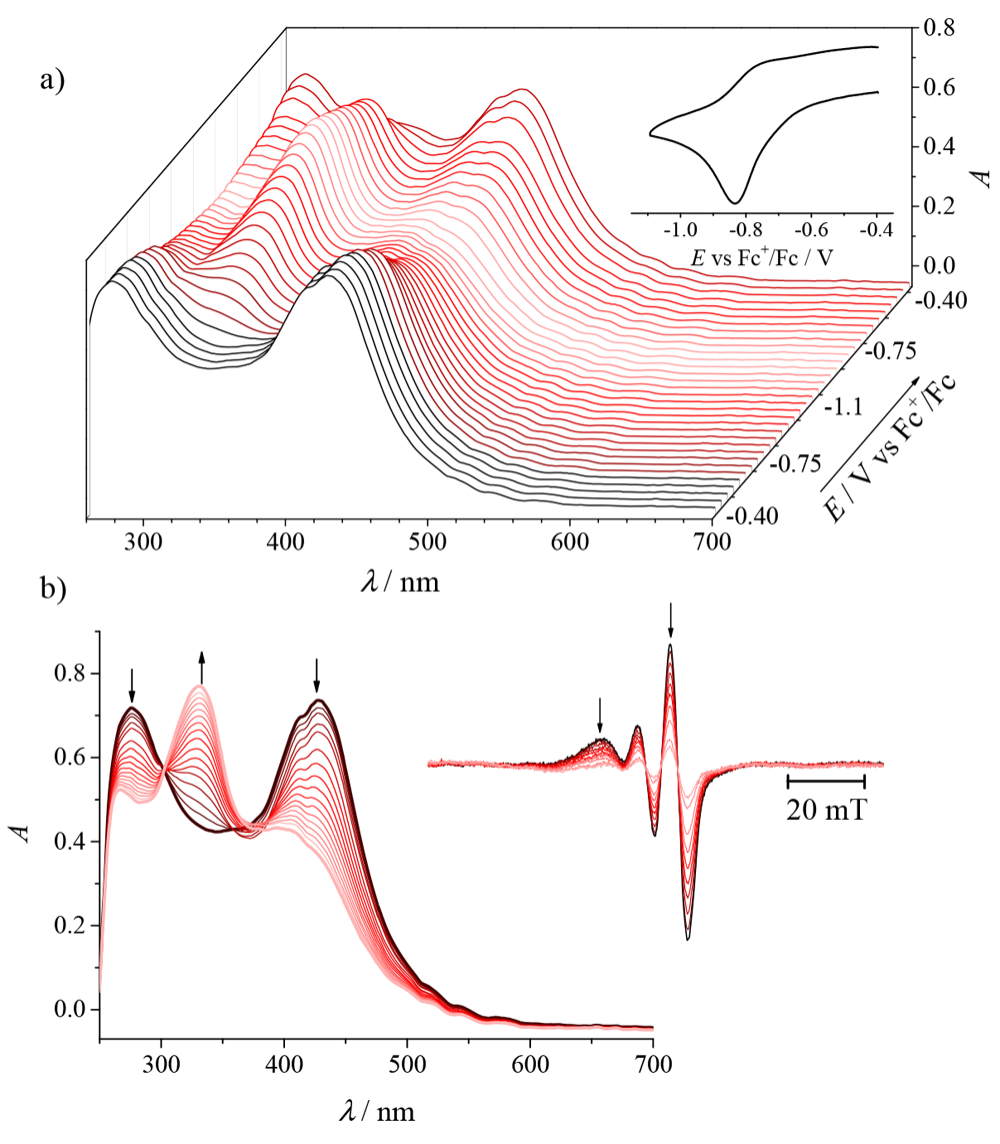
Spectroelectrochemistry of **1** in *n-*Bu_4_NPF_6_/DMSO in the region of the first cathodic peak: (a) potential dependence of UV–vis spectra with the corresponding in situ cyclic voltammogram (Pt-microstructured honeycomb working electrode, scan rate of 5 mV s^–1^); (b) evolution of UV–vis spectra in 2D projection upon forward scan. (inset) Evolution of EPR spectra measured at the first reduction peak using a Pt mesh working electrode.

**Figure 13:**
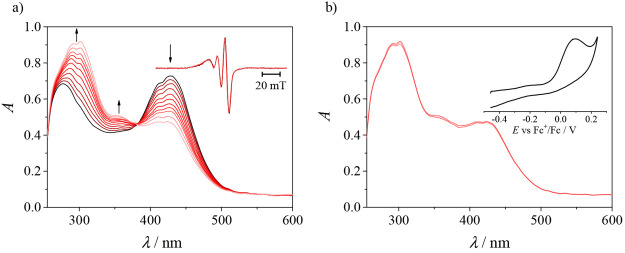
UV–vis spectra measured simultaneously (a) upon anodic oxidation of **1** in the region of the first anodic peak (inset: time evolution of EPR spectra acquired at the first anodic peak) and (b) upon the back scan (inset: the corresponding in situ cyclic voltammogram).

To support the assignment of the redox processes described previously, EPR/UV–vis spectroelectrochemical measurements were performed, and the results are shown for **1** in Figures 12 and 13. The UV–vis spectrum of **1** exhibits two absorption bands at 276 and 428 nm, where the first one is due to the absorption of the TSC ligand, while the second one can be attributed to the ligand-to-metal (S → Cu) charge transfer (LMCT).^73,74^ Upon the cathodic reduction of **1** in the region of the first reduction peak a new broad absorption band at 331 nm appears with a simultaneous decrease of the initial optical bands at 276 and 428 nm via an isosbestic point at 302 nm (Figure 12). An analogous spectroelectrochemical response was observed for **2′** as shown in Figure S13. This observation is different from that encountered by the reduction of the copper(II)–TSC complexes by GSH (vide supra), which led to the liberation of the ligand and formation of the copper(I) complex with GSH. In the spectroelectrochemical experiment in the absence of strong Cu(I) complexing agents, such as GSH, the TSC ligand may coordinate to Cu(I) and form a linear or tetrahedral complex. Upon the voltammetric reverse scan, a nearly full recovery of the initial optical bands was observed, which confirms the relatively good stability of cathodically generated Cu(I) complex with **HL**^**2**^ and, thus, the chemical reversibility of this redox process. Rare examples of four- and three-coordinate copper(I) complexes with potentially tridentate and bidentate thiosemicarbazones were reported previously.^75,76^ The room-temperature X-band EPR spectrum of **1** showed a typical signal for d^9^ Cu(II) species, which decreased stepwise upon a cathodic reduction at the first cathodic peak. This is in line with the metal-centered reduction and formation of EPR-silent d^10^ Cu(I) species^10^ (see inset in Figure 12b). EPR spectra of **1**, **2′**, and **3** measured in frozen *n-*Bu_4_NPF_6_/DMSO at 77 K show a characteristic axial symmetry (*g*_∥_ > *g*_⊥_ > *g*_e_) implying a square-planar coordination and the presence of one dominating species in DMSO (Figure S14).

The in situ cyclic voltammogram and simultaneously recorded evolution of UV–vis spectra upon an anodic oxidation of **1** in DMSO provide further evidence for the ligand-based irreversible oxidation. Spectral changes accompanying the oxidation of **1** are shown in Figure 13. These changes are characteristic for the other two complexes **2′** and **3** as well. Note that, in the region of the first oxidation peak, new optical bands at 295 and 356 nm appear with a simultaneous decrease of the initial absorption with a maximum at 428 nm (Figure 13a). However, the product formed upon oxidation is not reduced back during the reverse voltammetric scan (Figure 13b), indicating the chemical irreversibility of the redox process. In the EPR spectroelectrochemistry of **1** in DMSO/*n-*Bu_4_NPF_6_, no changes of the EPR signal were detected upon the oxidation at the first anodic peak, providing evidence of the two-electron oxidation process taking place on the TSC ligand.

The remarkable stability of copper(II) complexes **1**, **2′**, and **3** at a physiological pH, their moderate lipophilic character (log *D*_7.4_ = −0.4 to −0.42) and copper(II)/copper(I) redox activity (*E*_red_ = −0.83 to −0.93 V vs Fc^+^/Fc) in a biologically relevant window of redox potentials (−0.4 to +0.8 V vs NHE or −1.04 to 0.16 V vs Fc/Fc^+^) prompted the investigation of their antiproliferative activity in cancer cell lines.

## Inhibition of Cell Viability and Apoptosis Assay

### Cytotoxicity of the TSCs, Their Oxidized Products and Copper(II) Complexes

The in vitro cytotoxicity of the TSCs **HL**^**1**^**–HL**^**3**^, copper(II) complexes **Cu(HL**^**1**^**)Cl**_**2**_, **[Cu(L**^**2**^**)Cl]**, and **Cu(HL**^**3**^**)Cl**_**2**_, and oxidized TSCs **H*L***^**1a′**^, **H*L***^**1a″**^, and **H*L***^**2c′**^**·CH**_**3**_**COOH** was tested in the doxorubicin-sensitive Colo205 and the multidrug-resistant Colo320 human colonic adenocarcinoma cell lines as well as in normal human embryonal lung fibroblast cells (MRC-5) by the colorimetric MTT assay. The data that resulted (expressed as the half-maximal inhibitory concentration (IC_50_)) are collected in Table 2 and compared with those for triapine, doxorubicin, and CuCl_2_.

**Table 2:** In Vitro Cytotoxicity (IC_50_ Values in μM) of Metal-Free Ligands **HL**^**1**^**–HL**^**3**^, Copper(II) Complexes **Cu(HL**^**1**^**)Cl**_**2**_, **[Cu(L**^**2**^**)Cl]**, and **Cu(HL**^**3**^**)Cl**_**2**_, and, of the Oxidized Species **H*L***^**1a′**^, **H*L***^**1a″**^, and **H*L***^**2c′**^**·CH**_**3**_**COOH** in Colo205, Colo320, and MRC-5 Cell Lines after 72 h of Exposure

IC_50_ (μM)	Colo205	Colo320	MRC-5
**HL**^**1**^	>100	6.32 ± 0.49	>100
**HL**^**2**^	>100	>100	>100
**HL**^**3**^	48.2 ± 6.8	>100	>100
**Cu(HL**^**1**^**)Cl**_**2**_	2.08 ± 0.12	2.21 ± 0.18	3.13 ± 0.17
**[Cu(L**^**2**^**)Cl]**	0.181 ± 0.039	0.159 ± 0.009	0.276 ± 0.049
**Cu(HL**^**3**^**)Cl**_**2**_	26.6 ± 1.6	27.6 ± 1.6	>100
**H*L***^**1a′**^	>25	>25	>25
**H*L***^**1a″**^	>25	>25	>25
**H*L***^**2c′**^**·CH**_**3**_**COOH**	2.733 ± 0.059	0.188 ± 0.041	2.15 ± 0.10
CuCl_2_	19.7^a^	20.0^a^	24.5^a^
triapine	3.34 ± 0.12	4.21 ± 0.46	10.2 ± 1.3
doxorubicin	3.28^a^	3.12^a^	5.19^a^

a Data are taken from ref (77).

**Figure 14:**
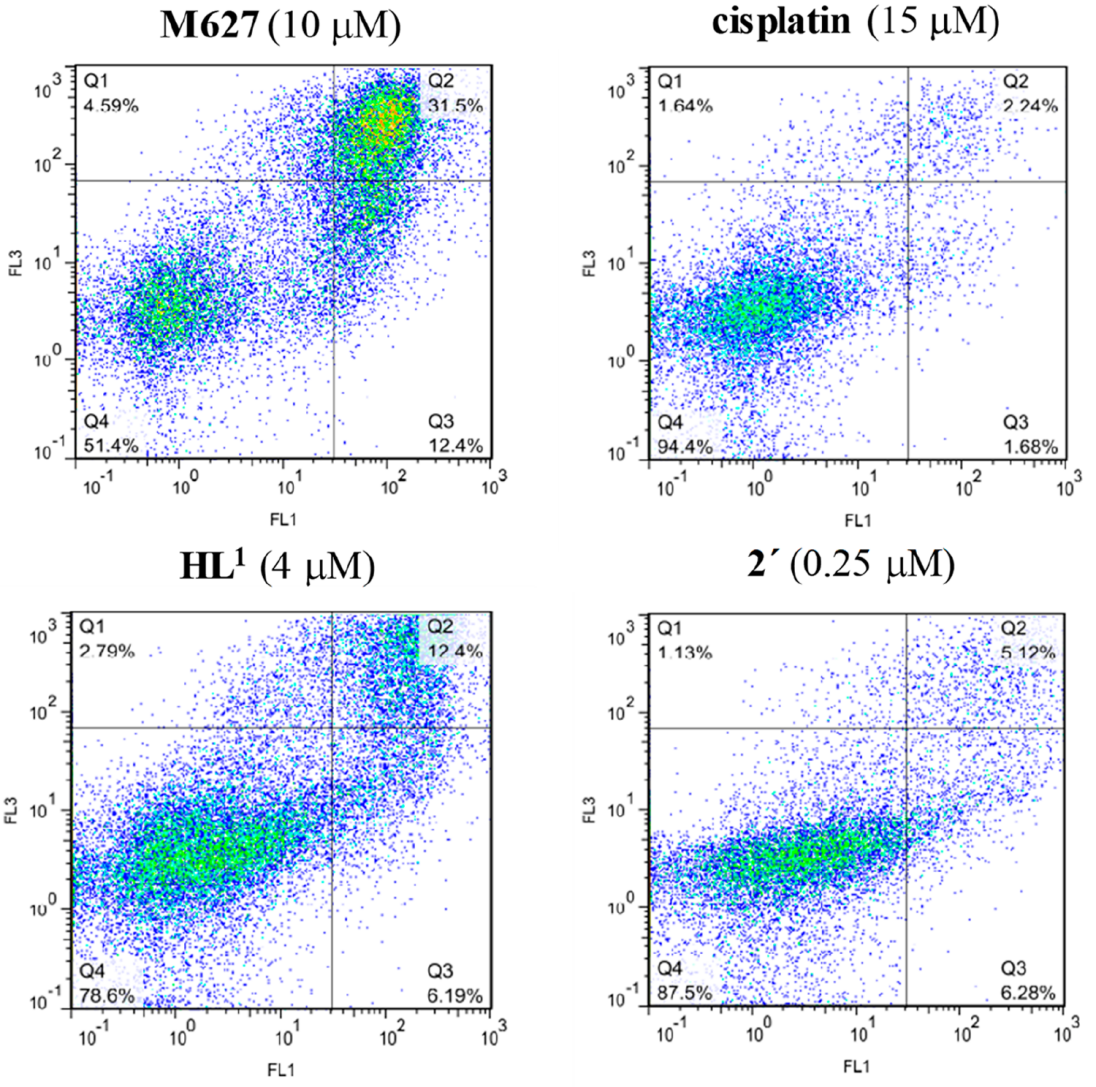
Quantification of apoptosis in Colo320 cells treated with **HL**^**1**^ and **2′** and M627 and cisplatin (as positive controls) using the Annexin-V/PI double staining assay. Colo320 cells were treated at the indicated concentration of the drugs. The dual parametric dot plots that combine the Annexin-V (FL1) and PI (FL3) fluorescence show the viable cell population in the lower-left quadrant Annexin-V–/PI– (**Q4**), the early apoptotic cells in the lower-right quadrant Annexin-V+/PI– (**Q3**), and the late apoptotic and necrotic cells in the upper-right quadrant Annexin-V+/PI+ (**Q2**). (Number of cells counted: 23 193 (M627), 20 262 (cisplatin), 33 193 (**HL**^**1**^), and 19 312 (**2′**)).

The metal-free ligands were either devoid of cytotoxicity or showed a weak response; only **HL**^**1**^ and **HL**^**3**^ revealed a somewhat higher activity against Colo320 and Colo205 cells, respectively, even though it was inferior to that of triapine. Notably, the copper(II) complexes are quite cytotoxic. So the effect of the copper(II) coordination is obvious in all cases. Low IC_50_ values (0.16–2.2 μM) were obtained for **Cu(HL**^**1**^**)Cl**_**2**_ and **[Cu(L**^**2**^**)Cl]** in both cancer cell lines (Colo205 and Colo320). To gain further insights into the cytotoxic behavior of the compounds, apoptosis induction by lead compounds **HL**^**1**^ and **[Cu(L**^**2**^**)Cl]** was investigated by a flow cytometry analysis of multidrug-resistant Colo320 cells stained with Annexin-V-FITC and propidium iodide (PI). The two compounds that displayed the highest cytotoxicity against this cell line were tested at two concentrations in the range of their IC_50_ values. 12*H*-Benzophenothiazine (M627) and cisplatin were used as positive controls. The fluorescence of PI (FL3) was plotted versus Annexin-V fluorescence (FL1) as shown in Figure 14 for the positive controls and for the tested compounds at a chosen concentration. The percentage of the gated events regarding the early apoptosis, the late apoptosis and necrosis, and cell death is quoted in Table S8. According to these data, both compounds studied, **HL**^**1**^ and **[Cu(L**^**2**^**)Cl]**, can be considered as efficient apoptosis inducers.

The antiproliferative activity of **1** and **2′** in the normal cells (MRC-5) was only slightly lower than in Colo205 and Colo320 cells, indicating a quite moderate selectivity for cancer cells. Complex **Cu(HL**^**3**^**)Cl**_**2**_ was found to be less cytotoxic compared to the other two complexes tested, and the IC_50_ values are similar to those of the copper(II) chloride, while the selectivity for cancer cells is obvious in this case (SI > 3). It is worth mentioning that the analogous α-*N*-pyridyl thiosemicarbazones, that is, FTSC, AcTSC, and triapine, were reported to be cytotoxic in the low micromolar concentration range against several human cancer cells, the latter being the most potent among them (IC_50_ values reported for triapine: 0.4–2.6 μM (in good agreements with the data quoted in Table 5), for FTSC: 1.9–10.6 μM, for AcFTSC: 2.5–3.6 μM in SW480,^36^ MES-SA,^36^ MES-SA/Dx5,^36^ HL60,^58^ 41M,^80^ SK-BR-3^80^).

Their Cu(II) complexes were reported to possess a similar or even weaker cytotoxicity compared to the metal-free ligands, in contrast to complexes studied in the present work, which might indicate a distinct mode of action. It is also of note that the two-electron oxidized product **H*L***^**2c′**^ revealed a superior antitumor activity in the two cancer cell lines over that of **H*L***^**1a′**^ and **H*L***^**1a″**^. In agreement with this, closely related 2-formyl- and 2-acetylpyridine 2-benzothiazolyl hydrazones were shown to be potent cytotoxic drugs against a series of 17 murine (e.g., L1210 lymphoid leukemia, P388 lymphocytic leukemia) and human cancer cells (e.g., HeLa cervix carcinoma, bone SOS, lung MB9812, lung A549). In addition, these compounds showed selectivity for the multidrug-resistant doxorubicin-selected uterine sarcoma cell line MES-SA/Dx5 over parental or sensitive MES-SA cells.^78,79^

### Tyrosyl Radical Reduction in mR2 RNR

**Figure 15:**
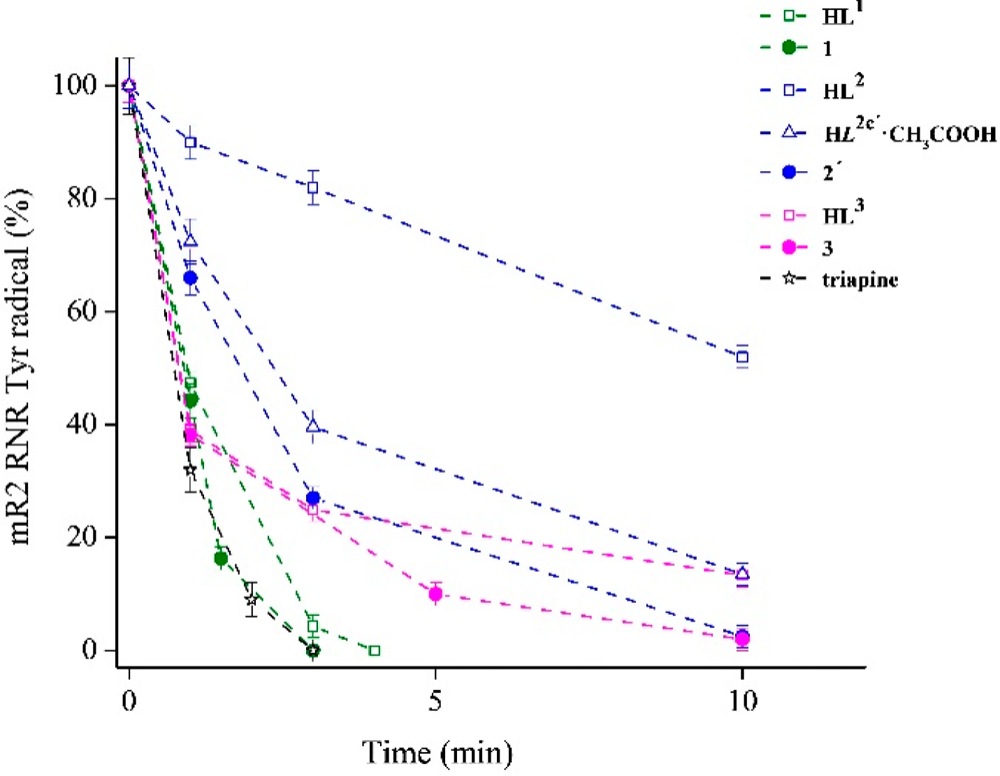
Tyrosyl radical reduction kinetics in mouse R2 RNR protein by TSCs **HL**^**1**^, **HL**^**2**^, **HL**^**3**^, and their corresponding copper complexes **1**, **2′**, and **3** as well as by the two-electron oxidized product of **HL**^**2**^ (**H*L***^**2c′**^**·CH**_**3**_**COOH**), in the presence of an external reductant, measured at 30 K by EPR spectroscopy and compared to triapine. The samples contained 20 μM mR2 in 50 mM HEPES buffer, pH 7.60/100 mM KCl, 20 μM compound in 1% (v/v) DMSO/H_2_O, and 2 mM DTT.

The TSCs **HL**^**1**^–**HL**^**3**^ and their copper(II) complexes **1**, **2′**, and **3** were found to effectively quench the tyrosyl radical in mR2 RNR in the presence of an external reductant (DTT). The time-dependent tyrosyl radical reduction in mR2 RNR by equimolar concentrations of TSCs and their respective copper(II) complexes, under reducing conditions, is shown in Figure 15. The mR2 inhibition potency follows the order **HL**^**1**^ ≈ triapine > **HL**^**3**^ > **HL**^**2**^. The coordination to copper(II) was found to increase the tyrosyl radical quenching potential for all TSCs, which is in agreement with the observed lowering of IC_50_ values in all cancer cell lines (Table 2). Complex **1** was shown to be as efficient as triapine,^17^ reducing 100% of the tyrosyl radical in 3 min. Complexes **2′** and **3** exhibited comparable reduction kinetics despite the fact that, among the investigated TSCs, **HL**^**2**^ was found to be most inefficient. The favorable impact of the copper(II) coordination on the **HL**^**2**^ inhibitory activity is quite obvious, when the ability to quench the tyrosyl radical by **HL**^**2**^ is compared to that of **2′**. Interestingly, the two-electron oxidized product of **HL**^**2**^, namely, **H*L***^**2c′**^**·CH**_**3**_**COOH**, is as potent as **HL**^**3**^ in tyrosyl radical reduction.

The ability of **HL**^**1**^–**HL**^**3**^ and **1**, **2′**, and **3** to quench the tyrosyl radical correlates well with their first anodic redox potentials (0.82–0.88 V vs NHE) and (0.68–0.70 V vs NHE), respectively, which are well-compared with redox potential of hydroxyurea (+0.724 V),^83^ which reduced the tyrosyl radical in the R2 protein with an estimated redox potential of 1.0 ± 0.1 V vs NHE.^36^ Note, however, that hydroxyurea, a well-known inhibitor of RNR and an anticancer drug,^84^ is a small molecule able to enter the hydrophobic R2 protein pocket, where the tyrosyl radical is buried. Finally, the two- and four-electron oxidized products of **HL**^**1**^, namely, **H*L***^**1a′**^ and **H*L***^**1a″**^, do not have an effect on the tyrosyl radical in the absence of DTT and, interestingly, cause an increase in the radical content in the presence of DTT (Figure S15).

Previously it has been shown that the radical content in mR2 may be slightly increased in the presence of DTT, as the result of the so-called radical reconstitution reaction,^17^^,85^ in which the DTT–reduced diiron center in the reaction with molecular oxygen is spontaneously oxidized through a series of intermediate states, generating the active Fe(III)-O^2–^-Fe(III)/Tyr· cofactor. However, the radical increase caused by **HL**^**1a′**^ and **HL**^**1a″**^ (in reducing conditions) is much greater than that observed for DTT, providing evidence that the formation of the active iron/radical site in mR2 is more efficient when the DTT–reduced form of mR2 is oxidized by **H*L***^**1a′**^ or **H*L***^**1a″**^, than by molecular oxygen only.

Consistent with enzyme inhibition studies, which revealed a potent inhibition of mR2 RNR, compounds **HL**^**1**^, **1**, and **2′** were found to increase the population of the S-phase in SW480 cells.

### Cell Cycle Arrest

**Figure 16:**
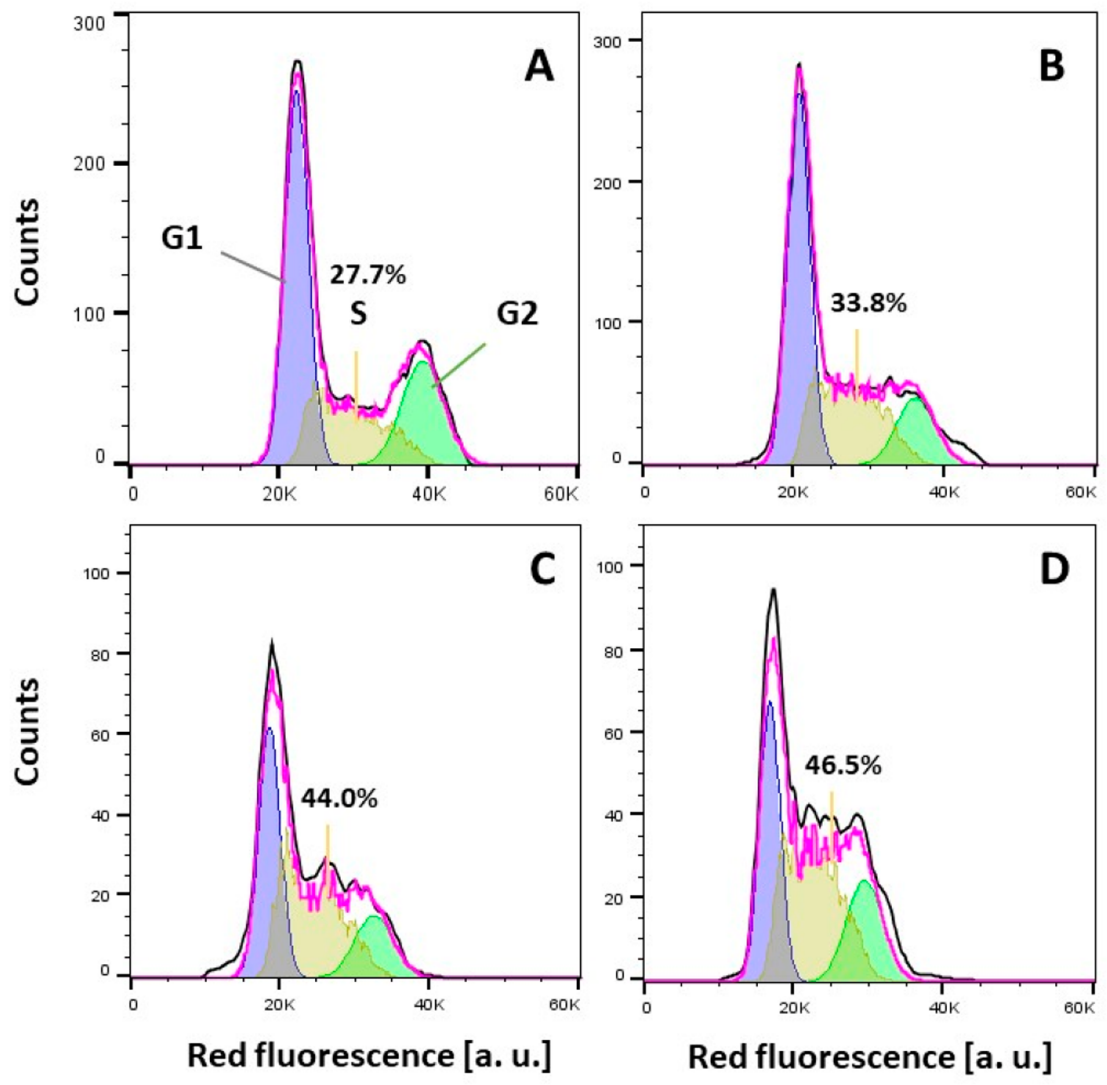
Flow cytometry analysis for a cell cycle distribution of SW480 cells induced by TSC **HL**^**1**^ (B) and complexes **1** (C) and **2′** (D) at the concentration of 10 μM for 24 h compared to the negative control (DMSO) (A).

The perturbation effects of 10 μM **HL**^**1**^, **1**, and **2′** on the cell cycle progression of SW480 cells when compared to negative control are shown in Figure 16 and Table S9, while the effects of 0, 1.0, and 10 μM are presented in Figure S16. It can be noted that the population of S-phase cells increased after an incubation with **HL**^**1**^ (37.1%), complex **1** (44.0), and **2′** (46.5) compared with the negative control (29.8%). Gemcitabine (GC), a positive control, showed a canonical G1/S-phase arrest at the concentration of 0.01 μM with 26.8% of cells in the G1 phase and 62.3% of cells in the S phase compared to the negative control with 49.1% of cells in the G1 phase and 29.8% of cells in the S phase (Figure S17). An increase in the population of the S-phase cells by ca. 20% has been reported for a series of triapine analogues at concentrations from 0.25 to 5.0 μM.^80^ The S-phase arrest is characteristic for cells treated with triapine.^81^

These data indicate that there is a correlation between the ability of the compounds tested to inhibit R2 RNR and their ability to induce an S-phase arrest. Nevertheless, the inhibition of RNR does not appear to be the main mechanism underlying the antiproliferative activity of both TSCs studied herein and their copper(II) complexes.

### ROS Generation

**Figure 17:**
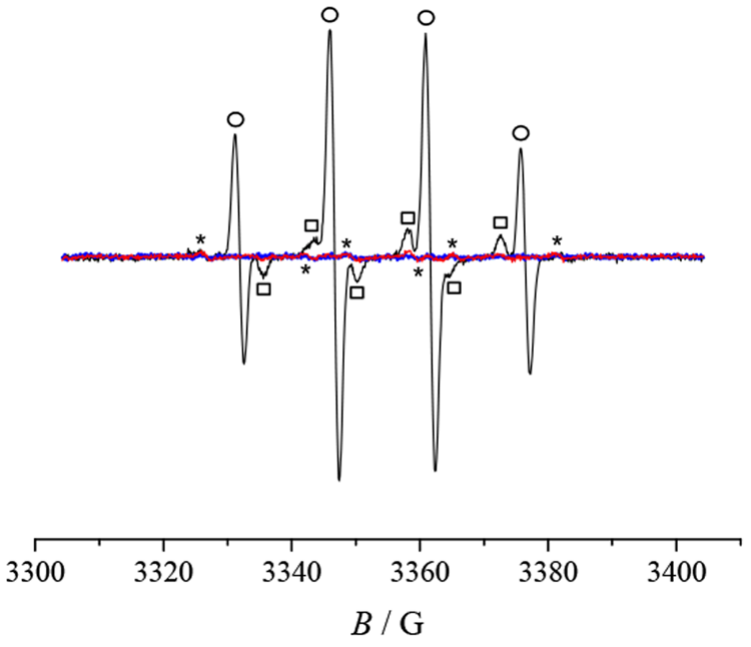
Experimental EPR spectra of Fe(II)/**HL**^**1**^/DMPO/H_2_O_2_ in 5% (v/v) H_2_O–DMSO (black line), in the system of Fe(II)/**HL**^**1**^/DMPO in 5% (v/v) H_2_O–DMSO (blue line), and in 30% (v/v) H_2_O–DMSO (red line). Initial concentrations: *c*(HL^1^) = 0.2 mM, *c*(FeSO_4_·7H_2_O) = 0.1 mM, *c*(DMPO) = 20 mM, *c*(H_2_O_2_) = 10 mM.

Since metal-free TSCs that enter the cells or are released from copper(I) complexes generated by a reduction of their copper(II) counterparts can react in the cells with iron(II), the redox activity of the **[Fe**^**II**^**(L**^**1**^**)**_**2**_**]** complex, prepared by the reaction of an anoxic aqueous solution of FeSO_4_·7H_2_O with a DMSO solution of **HL**^**1**^ at a 1:2 molar ratio, was investigated by EPR spin-trapping experiments. To investigate whether this ferrous complex is able to generate ROS in the aqueous environment by a Fenton reaction, which is supposed to quench the tyrosyl radical of the mR2 enzyme, hydrogen peroxide was added into the system in the presence of 5,5-dimethyl-1-pyrroline *N*-oxide (DMPO) as the spin-trapping agent. A four-line EPR signal characteristic for the ·OH-DMPO spin adduct was observed (Figure 17, black trace, EPR signal marked with circles).

Additionally a ·DMPO-OCH_3_ spin adduct can be seen in the corresponding EPR spectrum as a consequence of the reaction of hydroxyl radicals with the DMSO solvent forming methyl radicals, which react with molecular oxygen resulting in the generation of peroxomethyl radicals serving as a source of ^•^DMPO–OCH_3_ spin adducts (Figure 17, black trace, EPR signal marked with squares).^82^ Only a trace amount of carbon-centered radicals was detected for Fe(II)/**HL**^**1**^/DMPO in H_2_O–DMSO in the absence of H_2_O_2_ (Figure 17, blue and red traces, EPR signal marked with stars). In this case DMSO acts as a HO· scavenger, generating reactive carbon-centered radicals, which are trapped by DMPO. It is important to mention that no radicals were formed in the system of **HL**^**1**^/H_2_O_2_/DMPO/H_2_O–DMSO (not shown), which indicates the crucial role of the Fe(II) complex for ROS generation. Consequently complex **[Fe**^**II**^**(L**^**1**^**)**_**2**_**]** is redox-active in the Fenton reaction indicating the important role of the **HL**^**1**^ ligand for the observed antiproliferative activity against cancer cell lines and its ability to quench the tyrosyl radical in the mR2 protein. A direct reduction of the tyrosyl radical by iron(II) complexes with reported TSCs can also not be excluded.^16^

## Conclusions

New triapine analogues bearing a redox-active *p*-aminophenolic moiety and their copper(II) complexes have been synthesized and characterized by spectroelectrochemical and analytical techniques, which confirmed the noninnocent identity of the latter. The crystal structures of TSCs **HL**^**1**^–**HL**^**3**^ and complexes **[Cu(L**^**1**–**3**^**)Cl]** were studied by SC-XRD revealing the tridentate (N,N,S) coordination mode of the ligands. The presence of *E* and *Z* isomers of **HL**^**1**^–**HL**^**3**^ with a predominance of the first one in DMSO has been disclosed by 1D and 2D NMR spectroscopy. These data along with DFT calculations on the model compound 2-formylpyridine TSC indicate that the *Z*/*E* isomerization involves an inversion at the aldimine nitrogen atom, rather than a tautomeric shift of the thioamide N2H proton to the pyridine nitrogen, followed by a rotation around the C–N1 bond as suggested previously.^44^ The relatively high Gibbs free energy barrier (∼35.3 kcal/mol) for the *Z*/*E* conversion rules out the possibility of an isomerization at room temperature, in agreement with time-dependent NMR data.

A two-electron oxidative dehydrogenation of **HL**^**1**^ by a reaction with 1 equiv of DDQ afforded the new species **H*L***^**1a′**^ containing a thiadiazole five-membered ring formed via a nucleophilic attack of a thione sulfur atom on an aldimine carbon atom. This is supported by frontier molecular orbitals (MOs) with the HOMO and LUMO located at opposite parts of the molecule of **HL**^**1**^. When 2 equiv of DDQ were used, a further two-electron oxidation coupled with a two-proton loss occurred at the 3,5-dimethyl-4-aminophenolic moiety to give the 3,5-dimethyl-1,4-benzoquinone imine unit in **H*L***^**1a″**^. Also note that the coordinated ligand **HL**^**1**^ is able to form a thiazole five-membered ring in **4** via a sulfur attack on the carbon atom in position 2 or 6 of the 3,5-dimethyl-4-aminophenolic moiety. The arylated sulfur atom has lost the competition in binding to copper(II) for an end nitrogen atom due to the reduction of the electron-donating ability of the sulfur atom. The oxidation of **HL**^**2**^ with PBQ in a 1:1 molar ratio furnished the two-electron oxidative cyclization product **H*L***^**2b**^ and the diphenolic species **H*L***^**2e**^. A tentative mechanism of their formation is proposed. The pathway to **H*L***^**2e**^ implies the formation of the 4-isothiocyanato-2,6-dimethylphenol intermediate. Treatments of **HL**^**2**^ with 1 and 2 equiv of PIDA afforded the two-electron oxidation product **H*L***^**2c′**^ and the four-electron oxidation product **H*L***^**2c″**^, respectively. In contrast to **HL**^**1**^–**HL**^**3**^, the *Z*/*E* isomerization was observed at room temperature for **H*L***^**2c′**^. The isolation and investigation of oxidation products of new TSCs was of interest also from the point of view of collecting spectroscopic data that might be useful for an eventual analysis of metabolites, which can be generated in vivo from the corresponding TSCs and their copper(II) complexes.

Solution equilibrium studies performed by UV–vis spectrophotometry revealed the acidic p*K*_a_ values (3.01–3.95) of the pyridinium nitrogen and p*K*_a_ values greater than or equal to 10.55 for the hydrazinic-NNH and phenolic (PhOH) moiety of the metal-free ligands. The latter are neutral and stable at a physiological pH. However, they become air-sensitive upon deprotonation of the OH group in the basic pH range. The formation of high-stability monoligand copper(II) complexes was found in different protonation states in solution; namely, coordination via (N_pyridine_,N,S)(H_2_O), (N_pyridine_,N,S^–^)(H_2_O), and (N_pyridine_,N,S^–^)(OH^–^) donor sets are probable. The complexes with a (N_pyridine_,N,S^–^)(H_2_O) coordination predominate in a wide pH range including pH 7.4. Conditional stability constants determined for the **[Cu(L**^**1**^**)]**^**+**^ and **[Cu(L**^**3**^**)]**^**+**^ complexes by an EDTA UV–vis spectrophotometric competition experiment show the somewhat higher stability of the **[Cu(L**^**3**^**)]**^**+**^ complex. The attachment of a phenolic moiety undoubtedly increases the lipophilicity of new Schiff bases and copper(II) complexes when compared to triapine and its copper(II) complex. The new complexes can be reduced by glutathione, the most abundant low molecular mass reducing agent in a cell, in a reversible redox reaction. According to the electrochemical studies complexes **1**, **2′**, and **3** can undergo a redox process in a biologically accessible window (−0.4 to +0.8 V vs Fc^+^/Fc). These findings suggest a possible role of the redox properties of the copper(II) complexes in their biological activity.

The metal-free ligands and several oxidized products showed no or only a moderate cytotoxicity against doxorubicin-sensitive Colo205 and the multidrug-resistant Colo320 human colonic adenocarcinoma cell lines. Their copper(II) complexes revealed a high cytotoxic potency when compared to that of the corresponding metal-free ligands. **[Cu(L**^**2**^**)Cl]** showed the highest cytotoxic activity with IC_50_ values in the low micromolar concentration range and induced apoptosis, while **Cu(HL**^**3**^**)Cl**_**2**_ has the highest selectivity for cancer cells over the normal fibroblast MRC-5 cells. The highest antiproliferative activity of **[Cu(L**^**2**^**)Cl]** is likely due to the more negative reduction potential when compared to those of **1** and **3** and low reduction rate in reaction with GSH.^36^ In addition, **HL**^**1**^–**HL**^**3**^ and their copper(II) complexes were found to efficiently quench the tyrosyl radical in mR2 RNR in the presence of DTT as an external reductant and increase the population of S-phase cells. The capacity of **HL**^**1**^ to destroy the tyrosyl radical is almost identical with that of triapine, which is by the factor of 1000 a more potent R2 RNR inhibitor than hydroxyurea, a known clinical drug.^17^ Thus, the copper(II) complexes reported herein deserve further investigation as potential anticancer drugs.

## Supporting Information Available

Synthesis of oxidized thiosemicarbazones and their copper(II) complexes, methods used for characterization of the compounds, atom labeling schemes used in the NMR resonances assignment, NMR and UV–vis spectra showing *E*/*Z* isomerization of TSCs in solution, 2D NMR spectra, UV–vis spectra of TSCs at different pH values and measured over time, tyrosyl radical kinetic behavior in absence and presence of DTT, crystal data and details of data collection, collected multinuclear NMR data, summarized ESI mass spectra, computational details (PDF)

## Accession Codes

CCDC 2074017–2074030 and 2074341 contain the supplementary crystallographic data for this paper. These data can be obtained free of charge via www.ccdc.cam.ac.uk/data_request/cif, or by emailing data_request@ccdc.cam.ac.uk, or by contacting The Cambridge Crystallographic Data Centre, 12 Union Road, Cambridge CB2 1EZ, UK; fax: +44 1223 336033.
