# Supplementary material for: Triapine Analogues and Their Copper(II) Complexes: Synthesis, Characterization, Solution Speciation, Redox Activity, Cytotoxicity, and mR2 RNR Inhibition
Source: Inorg Chem. 2021 Jul 19;60(15):11297–319. doi: 10.1021/acs.inorgchem.1c01275 (PMC8335727; doi:10.1021/acs.inorgchem.1c01275)
Supplement: Supplementary file 1 — ic1c01275_si_001.pdf [file ic1c01275_si_001.pdf]

Supporting Information
for
Triapine analogues and their copper(II) complexes: synthesis, characterization, solution speciation, redox activity, cytotoxicity and mR2 RNR inhibition

Iuliana Besleaga,[∇] Iryna Stepanenko,[∇] Tatsiana V. Petrasheuskaya,[≠,‡] Denisa Darvasiova,[†] Martin Breza,[†] Marta Hammerstad,[◊] Małgorzata A. Marć,[≠,#] Alexander Prado-Roller, [∇] Gabriella Spengler,[#,‡] Ana Popović-Bijelić,[⊥] Eva A. Enyedy, [≠,‡,*] Peter Rapta,[†,*] Anatoly D. Shutalev,[Δ,*] Vladimir B. Arion[∇,*]

[∇]*University of Vienna, Institute of Inorganic Chemistry, Währinger Strasse 42, A-1090 Vienna, Austria*

[≠]*Department of Inorganic and Analytical Chemistry, Interdisciplinary Excellence Centre, University of Szeged, Dóm tér 7, H-6720 Szeged, Hungary*

[‡]*MTA-SZTE Lendület Functional Metal Complexes Research Group, University of Szeged, Dóm tér 7, H-6720 Szeged, Hungary*

[†]*Institute of Physical Chemistry and Chemical Physics, Faculty of Chemical and Food Technology, Slovak University of Technology in Bratislava, Radlinského 9, SK-81237 Bratislava, Slovak Republic*

[#]*Department of Medical Microbiology, Albert Szent-Györgyi Health Center and Faculty of Medicine, University of Szeged, Semmelweis utca 6, 6725-Szeged, Hungary*

[⊥]*Faculty of Physical Chemistry, University of Belgrade, Studentski trg 12-16, 11158 Belgrade, Serbia*

[Δ]*N. D. Zelinsky Institute of Organic Chemistry, Russian Academy of Sciences, 47 Leninsky Ave., 119991 Moscow, Russian Federation*

Table of Contents

1. Experimental details S3

1.1. Synthesis of oxidized thiosemicarbazones S3

1.2. Synthesis of copper(II) complexes with oxidized ligands S6

2. Characterization of the compounds S7

2.1. Physical measurements S7

2.2. Crystallographic Structure Determination S7

2.3. Spectroscopic studies: UV–vis titrations, kinetic measurements and lipophilicity determination S8

2.4. Spectroelectrochemical studies S9

2.5. *In vitro* cell studies S10

2.5.1. Cell lines and culture conditions S10

2.5.2. MTT assays S10

2.5.3. Mechanisms of cell death: assay for apoptosis induction S11

2.6. Tyrosyl radical reduction in mouse R2 RNR protein S11

2.7. Computational details S12

3. Schemes for assignment of resonances in HL^1 – HL^3 and their isomers (Scheme S1-S2) S12

4. Oxidative cyclization in the presence of Ag_2O (Scheme S3) S13

5. Schemes for assignment of resonances in $HL^{1a'}$, $HL^{1a''}$, $HL^{2c'}$, $HL^{2c''}$ and their isomers (Scheme S4-S7) S14

6. Oxidation of HL^2 with PBQ (Scheme S8) S16

7. Suggested structures for $[Cu(L^1)(H_2O)]^+$ and $[Cu(HL^1)(H_2O)]^{2+}$ (Chart S1) S17

8. Frontier orbitals in HL^2 (Figure S1) S18

9. ORTEP view of the ATSC (Figure S2) S18

10. *E*/*Z*-isomerization of $HL^{2c'}$ (Figure S3-S5) S19

11. ORTEP view of HL^{2e} and $[Cu(L^{1c'})Cl]$ (4) (Figure S6-S7) S20

12. Spectroscopic data (Figure S8-S11) S21

13. Spectroelectrochemical data (Figure S12-S13) S24

14. EPR measurement (Figure S14) S26

15. Tyrosyl radical reduction kinetics in mouse R2 RNR protein (Figure S15) S26

16. Cell cycle arrest (Figure S16-S17) S27

17. Crystal Data and Details of Data Collection (Table S1-S3) S29

18. ^1H and ^{13}C NMR Data (Table S4-S6) S32

19. Complexation of $HL^{1a'}$ and $HL^{1a''}$ (Table S7) S35

20. Cell apoptosis assay (Table S8-S9) S36

21. Computational data S37

References S73

1. Experimental details

1.1. Synthesis of oxidized thiosemicarbazones

N-(4-hydroxy-3,5-dimethylphenyl)-5-(pyridin-2-yl)-1,3,4-thiadiazol-2-amine ($\mathbf{H}\boldsymbol{L}^{1a'}$·$\mathbf{0.25H_2O}$). To a suspension of $\mathbf{HL^1}$·$\mathbf{0.5H_2O}$ (217 mg, 0.70 mmol) in methanol (10 mL) was added dropwise a solution of 2,3-dichloro-5,6-dicyano-1,4-benzoquinone (DDQ, 164 mg, 0.72 mmol) in methanol (10 mL). The reaction mixture was stirred at room temperature overnight. The yellow-greenish solid was filtered off, washed with methanol and dried in vacuo to give a first portion of material (108 mg). A second portion of the product (21 mg) was obtained from the filtrate by removal of the solvent and purification of the crude product by column chromatography on silica by using ethyl acetate as eluent. Second collected fraction with R_f = 0.8 is $\mathbf{H}\boldsymbol{L}^{1a'}$, while the first minor fraction with R_f = 0.93 is $\mathbf{H}\boldsymbol{L}^{1a''}$. Overall yield of $\mathbf{H}\boldsymbol{L}^{1a'}$·$\mathbf{0.25H_2O}$: 129 mg, 60.9%. Anal. Calcd for $C_{15}H_{14}N_4OS$·0.25H_2O (M_r = 302.87), %: C, 59.48; H, 4.83; N, 18.50; S, 10.5. Found, %: C, 59.41; H, 4.67; N, 18.35; S, 11.01. Positive ion ESI-MS for $C_{15}H_{14}N_4OS$ (MeCN/MeOH +1% H_2O): *m/z* 299.17 $[HL^{1a'}+H]^+$, 321.16 $[HL^{1a'}+Na]^+$, negative ion ESI-MS: *m/z* 296.94 $[HL^{1a'}-H]^-$. ^1H NMR (500 MHz, DMSO-d_6) δ 10.20 (s, 1H, H_{11}), 8.61 (d, *J* = 4.4 Hz, 1H, H_6), 8.11 (d, *J* = 7.9 Hz, 1H, H_3), 8.08 (s, 1H, H_{18}), 7.95 (td, *J* = 7.7, 1.7 Hz, 1H, H_4), 7.46 (ddd, *J* = 7.5, 4.9, 1.1 Hz, 1H, H_5), 7.16 (s, 2H, H_{13}+H_{17}), 2.17 (s, 6H, H_{19}+H_{20}). ^{13}C NMR (151 MHz, DMSO-d_6) δ 166.77 (C_{10}), 158.40 (C_7), 149.73 (C_6), 149.28 (C_2), 148.97 (C_{15}), 137.52 (C_4), 132.53 (C_{12}), 125.11 (C_{14}+C_{16}), 124.61 (C_5), 119.25 (C_3), 118.67 (C_{13}+C_{17}), 16.87 (C_{19}+C_{20}). IR (ATR, selected bands, $\tilde{\upsilon}_{max}$): 2904.09, 1586.36, 1493.98, 1427.72, 1217.70, 1114.60, 1029.35, 668.90 cm^{-1}. UV–vis (MeOH), λ_{max}, nm (ε, $M^{-1}cm^{-1}$): 261 (17281), 348 (20800). Crystals of $\mathbf{H}\boldsymbol{L}^{1a'}$·suitable for X-ray diffraction study were obtained upon re-crystallization from methanol.

N-(4-Oxocyclohexa-2,5-dien-1-ylidene)-5-(pyridin-2-yl)-1,3,4-thiadiazol-2-amine ($\mathbf{H}\boldsymbol{L}^{1a''}$·$\mathbf{0.5H_2O}$). To a suspension of $\mathbf{HL^1}$·$\mathbf{0.5H_2O}$ (50 mg, 0.16 mmol) in methanol (2.5 mL) was added dropwise a solution of 2,3-dichloro-5,6-dicyano-1,4-benzoquinone (DDQ, 75.6 mg, 0.33 mmol) in methanol (2.5 mL). The reaction mixture was stirred at room temperature for 1 h. Then the solvent was removed under reduced pressure and the bright orange product was purified by column chromatography on silica by using a mixture of ethyl acetate and chloroform (1:20) as eluent. The second fraction with R_f = 0.44 was collected. Yield: 71.63% (35 mg) as $\mathbf{H}\boldsymbol{L}^{1a''}$·$\mathbf{0.5H_2O}$. Anal. Calcd for $C_{15}H_{12}N_4OS$·0.5H_2O (M_r = 305.36): C, 59.00; H, 4.29; N, 18.35; S, 10.50 %. Found, %: C, 58.64; H, 3.90; N, 18.02; S, 10.40. Positive ion ESI-MS for $C_{15}H_{12}N_4OS$ (MeCN/MeOH +1% H_2O): *m/z* 297.18 $[HL^{1a''}+H]^+$, 319.20 $[HL^{1a''}+Na]^+$. ^1H NMR (500 MHz, DMSO-d_6) δ 8.71 (ddd, *J* = 4.8, 1.7, 1.0 Hz, 1H, H_6), 8.26 (dt, *J* = 7.9, 1.0 Hz, 1H, H_3), 8.06 (td, *J*

= 7.7, 1.7 Hz, 1H, H_4), 7.60 (ddd, J = 7.6, 4.8, 1.2 Hz, 1H, H_5), 7.46 (dq, J = 2.8, 1.3 Hz, 1H, H_{13}), 7.20 (dq, J = 2.7, 1.5 Hz, 1H, H_{17}), 2.05 (d, J = 1.5 Hz, 3H, H_{20}), 2.00 (d, J = 1.5 Hz, 3H, H_{19}). ^{13}C NMR (126 MHz, DMSO-d_6) δ 187.14 (C_{15}), 171.58 (C_{10}), 169.98 (C_7), 162.21 (C_{12}), 150.25 (C_6), 148.19 (C_2), 143.89 (C_{14}), 143.11 (C_{16}), 138.06 (C_4), 136.08 (C_{17}), 126.14 (C_5), 125.96 (C_{13}), 120.04 (C_3), 16.10 (C_{19}), 15.66 (C_{20}). IR (ATR, selected bands, $\tilde{\upsilon}_{max}$): 2961.43, 1641.11, 1541.66, 1323.64, 1211.98, 1008.08, 942.99, 899.83, 778.65 cm^{-1}. UV–vis (MeOH), λ_{max}, nm (ε, M^{-1}cm^{-1}): 302 (36457), 410 sh. Crystals of **H*L*$^{1a''}$** suitable for X-ray diffraction study were obtained from DMSO-d_6.

2-Acetylpyridine (6-hydroxy-5,7-dimethyl-1,3-benzothiazol-2-yl)hydrazone (H*L*$^{2c'}$). A solution of phenyliodine(III) diacetate (PIDA, 205.2 mg, 0.64 mmol) in glacial acetic acid (3 mL) was added dropwise to a cold suspension of **HL^2·0.2H$_2$O** (200 mg, 0.63 mmol) in glacial acetic acid (7 mL). The solution was stirred at room temperature for 1 h. Then the solvent was partially removed under reduced pressure and the concentrated solution was left for crystallization at 4 °C. The light rosa precipitate of **H*L*$^{2c'}$·CH$_3$COOH** was filtered off, washed with cold methanol and dried *in vacuo*. Yield: 142.5 mg, 60.73%. Anal. Calcd for $C_{16}H_{16}N_4OS\cdot CH_3COOH$ (M_r = 372.44): C, 58.05; H, 5.41; N, 15.04; S, 8.61%. Found, %: C, 58.51; H, 5.38; N, 15.41; S, 8.58. ^{1}H NMR (600 MHz, DMSO-d_6) δ 11.76 (s, 2H, H_9+N*H*), 8.58 (d, J = 5.5 Hz, 1H, H_6), 8.18 (s, 1H, H_{18}), 8.08 (d, J = 8.0 Hz, 1H, H_3), 7.85 (td, J = 7.7, 1.4 Hz, 1H, H_4), 7.36 (dd, J = 6.7, 5.4 Hz, 1H, H_5), 7.02 (s, 1H, H_{13}), 2.40 (s, 3H, $H_{7'}$), 2.28 (s, 3H, H_{20}), 2.22 (s, 3H, H_{19}), 1.91 (s, 3H, C*H*$_3$COOH). ^{13}C NMR (151 MHz, DMSO-d_6) δ 172.00 (*C*=O, CH$_3$*C*OOH), 166.47 (C_{10}), 155.22 (C_2), 153.88 (C_{12}), 149.29 (C_7), 148.57 (C_6), 148.14 (C_{15}), 136.43 (C_4), 126.88 (C_{17}), 124.07 (C_{14} or C_{16}), 123.35 (C_5), 119.65 (C_3), 117.44 (C_{14} or C_{16}), 115.78 (C_{13}), 21.04 (*C*H$_3$, *C*H$_3$COOH), 17.08 (C_{19}), 15.47 (C_{20}), 12.55 ($C_{7'}$). ^{15}N NMR (61 MHz, DMSO-d_6) δ 337.75 (N_8), 309.86 (N_1). IR of (ATR, selected bands, $\tilde{\upsilon}_{max}$): 2322.13, 1571.13, 1426.14, 1201.77, 1038.02, 1013.17, 781.99, 643.45 cm^{-1}. UV–vis (MeOH), λ_{max}, nm (ε, M^{-1}cm^{-1}): 255 sh, 345 (18303), 465 sh. Then **H*L*$^{2c'}$·CH$_3$COOH** (120 mg, 0.32 mmol) was dissolved in water (10 mL) and pH was adjusted to 7-8 with a saturated solution of NaHCO$_3$. The crude **H*L*$^{2c'}$** was extracted with ethyl acetate (4 × 30 mL). Evaporation of ethyl acetate to ¼ of original volume afforded the first portion of *E*-isomer, which was isolated by filtration and drying in air (53 mg of beige powder). The filtrate was concentrated and purified chromatographically on silica by using EtOAc as eluent. A second portion of the *E*-isomer of **H*L*$^{2c'}$** (10 mg) was obtained by collecting the third fraction (fr$_3$, Rf = 0.63). Overall yield of *E*- **H*L*$^{2c'}$**: (63 mg, 63%) as **H*L*$^{2c'}$**. Second fraction (fr$_2$, R_f = 0.77) was collected as an orange powder and identified as the *Z*-isomer of **H*L*$^{2c'}$**. Yield: 10 mg, 10.0%. The first red fraction (fr$_1$, R_f = 0.92) was

identified as **H*L*$^{2c''}$**. Anal. Calcd for $C_{16}H_{16}N_4OS$ (*E*-isomer, M_r = 312.39): C, 61.52; H, 5.16; N, 17.93; S, 10.26%. Found, %: C, 61.49; H, 5.21; N, 17.55; S, 10.01. Positive ion ESI-MS for $C_{16}H_{16}N_4OS$, *E*/*Z*-isomers of **H*L*$^{2c'}$** (MeCN/MeOH +1% H_2O): *m*/*z* 313.21 [H*L*$^{2c'}$+H]$^+$, negative ion ESI-MS: *m*/*z* 310.98 [H*L*$^{2c'}$–H]$^-$. ^{1}H NMR (500 MHz, DMSO-*d*$_6$, *E*-isomer) δ 11.58 (s, 1H, H_9), 8.58 (ddd, *J* = 4.8, 1.7, 0.9 Hz, 1H, H_6), 8.20 (s, 1H, H_{18}), 8.07 (d, *J* = 8.1 Hz, 1H, H_3), 7.85 (td, *J* = 7.8, 1.7 Hz, 1H, H_4), 7.36 (ddd, *J* = 7.4, 4.9, 1.0 Hz, 1H, H_5), 7.02 (brs, 1H, H_{13}), 2.40 (s, 3H, $H_{7'}$), 2.28 (s, 3H, H_{20}), 2.22 (s, 3H, H_{19}). ^{13}C NMR (151 MHz, DMSO-*d*$_6$, *E*-izomer) δ 155.15 (C_2), 148.57 (C_6), 148.15 (C_{15}), 136.44 (C_4), 124.07 (C_{14} or C_{16}), 123.35 (C_5), 119.65 (C_3), 117.80 (C_{14} or C_{16}), 17.09 (C_{19}), 15.54 (C_{20}), 12.56 ($C_{7'}$). ^{1}H NMR (500 MHz, DMSO-*d*$_6$, *Z*-isomer) δ 15.00 (s, 1H, H_9), 8.85 (d, *J* = 3.5 Hz, 1H, H_6), 8.22 (s, 1H, H_{18}), 8.08 (t, *J* = 7.3 Hz, 1H, H_4), 7.79 (d, *J* = 7.7 Hz, 1H, H_3), 7.58 (m, 1H, H_5), 7.15 (s, 1H, H_{13}), 2.40 (s, 3H, $H_{7'}$), 2.31 (s, 3H, H_{20}), 2.22 (s, 3H, H_{19}). ^{13}C NMR (151 MHz, DMSO-*d*$_6$, *Z*-izomer) δ 152.61 (C_2), 148.39 (C_{15}), 147.63 (C_6), 138.29 (C_4), 124.26 (C_5), 124.08 (C_3), 118.98 (C_{13}), 117.67 (C_{14} or C_{16}), 21.72 ($C_{7'}$), 17.20 (C_{19}), 15.87 (C_{20}). IR (*E*-isomer, ATR, selected bands, $\tilde{\upsilon}_{max}$): 3309.21, 1565.44, 1462.63, 1428.84, 1213.07, 1149.94, 1103.32, 1016.42 cm^{-1}. IR (*Z*-isomer, ATR, selected bands, $\tilde{\upsilon}_{max}$): 2921.95, 2854.29, 2322.04, 1727.80, 1541.22, 1463.34, 1063.33, 832.32, 648.31 cm^{-1}. UV–vis (*E*-isomer, MeOH, λ_{max}, nm (ε, M^{-1}cm^{-1})): 256 (16778), 346 (23875), 464 sh. UV–vis (*Z*-isomer, MeOH, λ_{max}, nm (ε, M^{-1}cm^{-1})): 266 (3638), 367 (4387), 474 sh.

5,7-Dimethyl-2-{[1-(pyridin-2-yl)ethylidene]hydrazineylidene}benzo[*d*]thiazol-6(2*H*)-one (H*L*$^{2c''}$). A solution of phenyliodine(III) diacetate (PIDA, 103 mg, 0.32 mmol) in glacial acetic acid (4 mL) was added to a suspension of **HL^2·0.2H$_2$O** (50 mg, 0.16 mmol) in glacial acetic acid (1 mL). The solution was stirred at room temperature for 1.5 h. Then the solvent was partially removed under reduced pressure and the concentrated solution was left for crystallization at 4 °C. The red precipitate of **H*L*$^{2c''}$** was filtered off and washed with cold methanol. The collected precipitate was recrystallized from chloroform and afterwards dried *in vacuo*. Yield: 31 mg, 48.4%. Anal. Calcd for $C_{16}H_{14}N_4OS{\cdot}0.75CHCl_3$ (M_r = 399.91): C, 50.31; H, 3.72; N, 14.01; S, 8.02%. Found, %: C, 50.32; H, 3.67; N, 14.24; S, 8.43. Positive ion ESI-MS for $C_{16}H_{14}N_4OS$ (MeCN/MeOH +1% H_2O): *m*/*z* 311.12 [H*L*$^{2c''}$+H]$^+$, 333.11 [H*L*$^{2c''}$+Na]$^+$, 643.14 [2H*L*$^{2c''}$+Na]$^+$, negative ion ESI-MS: *m*/*z* 309.01 [H*L*$^{2c''}$–H]$^-$. ^{1}H NMR (500 MHz, DMSO-*d*$_6$) δ 8.73 (s, 1H), 8.25 (d, *J* = 5.7 Hz, 1H), 8.06 – 7.91 (m, 1H), 7.65 (s, 1H), 7.57 (s, 1H), 2.61 (s, 3H), 2.10 (s, 3H), 2.06 (s, 3H). ^{1}H NMR (500 MHz, $CDCl_3$) δ 8.70 (d, *J* = 4.3 Hz, 1H, H_6), 8.34 (d, *J* = 8.0 Hz, 1H, H_3), 7.80 (td, *J* = 7.8, 1.3 Hz, 1H, H_4), 7.44 (brs, 1H, H_{13}), 7.38 (dd, *J* = 6.7, 5.2 Hz, 1H, H_5), 2.72 (s, 3H, $H_{7'}$), 2.18 (s, 3H, H_{19}), 2.14 (s, 3H, H_{20}). ^{13}C NMR (151 MHz, $CDCl_3$) δ 184.43 (C_{15}), 173.74

(C_{10}), 169.74 (C_{12}), 169.42 (C_7), 154.97 (C_2), 149.29 (C_6), 145.86 (C_{14} or C_{16}), 145.41 (C_{14} or C_{16}), 136.46 (C_4), 129.52 (C_{13}), 129.25 (C_{17}), 125.34 (C_5), 122.31 (C_3), 17.38 (C_{19}), 15.54 (C_{20}), 14.88 ($C_{7'}$). IR (ATR, selected bands, $\tilde{\upsilon}_{max}$): 2918.15, 1616.23, 1584.52, 1464.22, 1362.22, 1245.16, 1157.17, 907.55, 780.68, 744.00, 687.98 cm^{-1}. UV–vis (MeOH), λ_{max}, nm (ε, $M^{-1}cm^{-1}$): 290 (8232), 391 (12367), 480 (8985). Crystals of **H*L*$^{2c''}$·0.5CHCl$_3$** suitable for X-ray diffraction study were obtained from chloroform solution.

4-(4-Hydroxy-3,5-dimethylphenyl)-5-methyl-5-(pyridin-2-yl)-4,5-dihydro-3H-1,2,4-triazole-3-thione (**H*L*^{2b}**) and **2-acetylpyridine {5-hydroxy-3-[(4-hydroxy-3,5-dimethylphenyl)amino]-4,6-dimethyl-1*H*-indol-2-yl}hydrazone** (**H*L*^{2e}**). A solution of *p*-benzoquinone (PBQ, 27 mg, 0.25 mmol) in methanol (1 mL) was added to suspension of **HL^2·0.2H$_2$O** (80 mg, 0.25 mmol) in methanol (8 mL). The reaction mixture was heated at 60 °C overnight. The methanol was removed by evaporation, the residue was chromatographed, using as eluent, mixture of ethyl acetate, hexane and methanol (2:6:1) to obtain the oxidation products **H*L*^{2b}** (R_f = 0.69) and **H*L*^{2e}** (R_f = 0.57) in small amounts. Positive ion ESI-MS for $C_{16}H_{16}N_4OS$ (**H*L*^{2b}**) (MeCN/MeOH +1% H_2O): *m/z* 313.25 [H*L*^{2b}+H]$^+$, 335.14 [H*L*^{2b}+Na]$^+$, negative ion ESI-MS: *m/z* 310.99 [H*L*^{2b}–H]$^-$. ^{1}H NMR (**H*L*^{2b}**, 500 MHz, DMSO-*d$_6$*) δ 8.67 (s, 1H, O*H*), 8.65 (ddd, *J* = 4.7, 1.7, 0.8 Hz, 1H, *H*py), 7.94 (td, *J* = 7.8, 1.8 Hz, 1H, *H*py), 7.59 (d, *J* = 8.0 Hz, 1H, *H*py), 7.51 (ddd, *J* = 7.6, 4.7, 0.9 Hz, 1H, *H*py), 6.39 (s, 2H, *H*ph), 2.06 (s, 3H, C*H*$_3$), 2.01 (s, 6H, 2C*H*$_3$). UV–vis (**H*L*^{2b},** MeOH), λ_{max}, nm (ε, $M^{-1}cm^{-1}$): 261 sh, 296 (5339), 346 (4985). Crystals suitable for X-ray diffraction study were obtained from ethanol (**H*L*^{2b}**) or methanol (**H*L*^{2e}**) solutions.

1.2. Synthesis of copper(II) complexes with oxidized ligands

[Cu(H*L*^{1d})$_2$Cl$_2$] (5). $CuCl_2·2H_2O$ (23 mg, 0.13 mmol) was added to **H*L*$^{1a''}$·0.5H$_2$O** (40 mg, 0.13 mmol) in methanol (25 mL) and stirred at 80 °C for 72 h. The reaction mixture was allowed to stand at 4 °C overnight. The bright green precipitate was filtered off, washed with methanol and dried *in vacuo*. Yield: 19.5 mg. Positive ion ESI-MS for $C_{14}H_{12}N_8S_2CuCl_2$ (MeCN/MeOH+1% H_2O): *m/z* 209.50 [Cu(H*L*^{1d})$_2$]$^{2+}$, 419.03 [Cu^I(H*L*^{1d})$_2$]$^+$, 453.93 [Cu(H*L*^{1d})$_2$Cl]$^+$, 459.05 [Cu(H*L*^{1d})$_2$$^{2+}$+MeCN–H]$^+$. IR (ATR, selected bands, $\tilde{\upsilon}_{max}$): 3226.02, 3087.67, 2322.41, 1608.77, 1513.30, 1420.89, 1351.58, 1317.22, 1150.55, 782.01, 720.75 cm^{-1}. UV–vis (MeOH), λ_{max}, nm (ε, $M^{-1}cm^{-1}$): 249 (15476), 281 sh, 343 (13271). Crystals of **[Cu(H*L*^{1d})$_2$Cl$_2$]** suitable for X-ray diffraction study were grown from methanolic solution of **5**.

[Cu(H*L*$^{2c'}$)Cl$_2$] (6). $CuCl_2·2H_2O$ (11 mg, 0.06 mmol) was added in one portion under argon to solution of **H*L*$^{2c'}$** (20 mg, 0.06 mmol) in anoxic methanol (5 mL) and stirred at room temperature

for 1 h. The reaction mixture was allowed to stand at +4 °C overnight. The brown-reddish precipitate was filtered off, washed with anoxic methanol and dried *in vacuo*. Yield: 19 mg. Positive ion ESI-MS for $C_{16}H_{16}N_4OSCuCl_2$ (MeCN/MeOH+1% H_2O): *m/z* 374.08 [Cu(H$L^{2c\prime}$)$^{2+}$–H]$^+$, 410.05 [Cu(H$L^{2c\prime}$)Cl]$^+$, 820.95 [2Cu($L^{2c\prime}$)Cl+H]$^+$, negative ion ESI-MS: *m/z* 407.93 [Cu(H$L^{2c\prime}$)Cl$^+$–2H]$^-$, 854.75 [2Cu($L^{2c\prime}$)+3Cl]$^-$. IR (ATR, selected bands, $\tilde{\upsilon}_{max}$): 2322.16, 1637.16, 1595.67, 1528.72, 1464.37, 1418.27, 1227.93, 1178.21, 1097.33, 1010.12, 896.16 cm^{-1}. UV–vis (MeOH), λ_{max}, nm (ε, M^{-1}cm^{-1}): 288 sh, 335 sh, 402 sh, 473 (5181). Crystals of **[Cu(H$L^{2c\prime}$)Cl$_2$]** suitable for X-ray diffraction study were grown by slow diffusion of diethyl ether in methanol solution of **6**.

2. Characterization of the compounds

2.1. Physical measurements

Elemental analyses were carried out in a Carlo-Erba microanalyzer at the Microanalytical Laboratory of the University of Vienna. Electrospray ionization mass spectrometry (ESI-MS) was carried out with amaZon speed ETD Bruker instrument. Expected and experimental isotope distributions were compared. UV–vis spectra were measured on Perkin Elmer UV–vis spectrophotometer Lambda 35 in the 240 to 700 nm window using samples dissolved in methanol. IR spectra were recorded on a Bucker Vertex 70 Fourier transform IR spectrometer (4000–600 cm^{-1}) using the ATR technique. 1D (^{1}H, ^{13}C) and 2D (^{1}H-^{1}H COSY, ^{1}H-^{1}H TOCSY, ^{1}H-^{1}H NOESY, ^{1}H-^{13}C HSQC, ^{1}H-^{13}C HMBC, ^{1}H-^{15}N HSQC, ^{1}H-^{15}N HMBC) NMR spectra were acquired on a Bruker AV NEO 500 or AV III 600 spectrometers in DMSO-d_6, MeOH-d_4, $CDCl_3$ at 25 °C.

2.2. Crystallographic Structure Determination

X-ray diffraction measurements were performed on either Bruker X8 APEX-II CCD or Bruker D8 Venture diffractometer. Single crystals were positioned at 30, 40, 27, 27, 26, 27, 26, 26, 26, 30, 30, 27, 30 and 26 mm from the detector, and 897, 158, 2225, 500, 1499, 2477, 884, 1279, 2395, 360, 360, 1081, 1959 and 1000 frames were measured, each for 2, 10, 10, 40, 20, 1, 15, 5, 4, 3, 10, 50, 10 and 2 s over 0.6, 1, 0.5, –0.360, 0.360, 0.360, –0.360, 0.360, 0.360, 0.5, 0.5, 0.5, 0.360 and –0.360° scan width for **HL^1·C$_2$H$_5$OH**, **HL^2**, **HL^3**, **H$L^{1a\prime}$**, **H$L^{1a\prime\prime}$**, **ATSC´**, **HL^{2b}**, **HL$^{2c\prime\prime}$·0.5CHCl$_3$**, **HL^{2e}**, **1´·CH$_3$OH**, **3´·CH$_3$OH**, **4–6**, respectively. Compound **2´** was measured at the XRD2 structural biology beamline, Elettra synchrotron. The beamline is equipped with an Arinax MD2S diffractometer and a Pilatus 6M area detector and monochromated X-rays are provided by a

superconducting wiggler followed by a dual crystal monochromator. Frame integration was performed using the XDS package.[1] Crystal data, data collection parameters, and structure refinement details are given in Tables S1–S3 in Supporting Information. The structures were solved by direct methods and refined by full-matrix least-squares techniques. Non-H atoms were refined with anisotropic displacement parameters. H atoms were inserted in calculated positions and refined with a riding model. The following computer programs and hardware were used: structure solution, *SHELXS-2014* and refinement, *SHELXL-2014*;[2] molecular diagrams, *ORTEP*; computer, Intel CoreDuo. CCDC 2074027 (**HL^1·C$_2$H$_5$OH**), 2074029 (**HL^2**), 2074028 (**HL^3**), 2074023 (**H*L*$^{1a'}$**), 2074017 (**H*L*$^{1a''}$**), 2074018 (**ATSC´**), 2074019 (**H*L*^{2b}**), 2074341 (**HL$^{2c''}$·0.5CHCl$_3$**) 2074025 (**H*L*^{2e}**), 2074020 (**1´·CH$_3$OH**), 2074022 (**2´**), 2074021 (**3´·CH$_3$OH**), 2074030 **(4**), 2074026 (**5**) and 2074024 (**6**).

2.3. Spectroscopic studies: UV–vis titrations, kinetic measurements and lipophilicity determination

An Agilent Carry 8454 diode array spectrophotometer was used to record the UV–vis spectra in the interval 200–800 nm. The path length was 1 cm. Proton dissociation constants (pK_a) of the proligands were calculated by the computer program PSEQUAD.[3] Spectrophotometric titrations were performed on samples containing the proligands at 50-100 µM concentration by a KOH solution in the presence of 0.1 M KCl at 25.0 ± 0.1 °C in the pH range from 1 to 12.5 in 30% (v/v) DMSO/H_2O solvent mixture. An Orion 710A pH-meter equipped with a Metrohm combined electrode (type 6.0234.100) and a Metrohm 665 Dosimat burette were used for the titrations. The electrode system was calibrated to the pH = −log[H$^+$] scale by means of blank titrations (HCl vs KOH) according to the method suggested by Irving *et al.*[4] The average water ionization constant (pK_w) is 14.54 ± 0.05 in 30% (v/v) DMSO/H_2O, which corresponds well to literature data.[5] Argon was also passed over the solutions during the titrations. Measurements were carried out in the range *ca*. 1.0–2.0 by preparing individual samples in which KCl was partially or completely replaced by HCl and pH values were calculated from the strong acid content. The conditional stability constants (*K*') of the copper(II) complexes were calculated at pH 5.90 based on the spectral changes via the displacement reaction with EDTA in the presence 50 mM MES and 0.1 M KCl in 30% (v/v) DMSO/H_2O. In the competition experiments the samples contained 25 µM copper(II), 25 µM proligand and the concentration of EDTA was varied in the range from 0 to 150 µM. The conditional stability constants of the metal complexes (*K*') and the individual spectra of the species were calculated by the computer program PSEQUAD.[3]

The time-dependence of UV–vis absorption spectra was monitored for proligands **HL²** at pH 7.4, 1.5 and 10.0 in 30% (v/v) DMSO/H_2O, and for **HL¹** and **HL³** at pH 7.4.

The redox reaction of the copper(II) complexes (25 µM) with GSH (1250 µM) was studied at 7.40 (50 mM HEPES buffer with 0.1 M KCl) and at 25.0 ± 0.1 °C on Hewlett Packard 8452A diode array spectrophotometer using a special, tightly closed tandem cuvette (Hellma Tandem Cell, 238-QS) as described previously.[6,7] Calculation of the observed rate constants (k_{obs}) of the redox reaction was performed by using a reported approach.[6,7]

Distribution coefficients ($D_{7.4}$) values were determined by the traditional shake-flask method in *n*-octanol/buffered aqueous solution at pH 7.40 (50 mM HEPES buffer at 25.0 ± 0.2 °C as described previously in our previous work.[8]

2.4. Spectroelectrochemical studies

Cyclic voltammetry experiments with 0.5 mM solutions of Cu(II) complexes and corresponding ligands in 0.1 M *n*-Bu_4NPF_6 in both DMSO and methanol were performed under argon atmosphere using a three-electrode arrangement with glassy carbon 1 mm disc working electrode (from Ionode, Australia), platinum wire as counter electrode, and silver wire as pseudo-reference electrode. As a supporting electrolyte, 0.1 M *n*-Bu_4NPF_6 was used in DMSO (SeccoSolv max. 0.025% H_2O, Merck) and 0.1 M $LiClO_4$ (≥95.0%, Merck) in methanol. Ferrocene (Fc, from Sigma-Aldrich) served as the internal potential standard. The potentials vs Fc^+/Fc in DMSO solutions were recalculated to the corresponding values vs NHE using known value of $E_{1/2}$(Fc^+/Fc) = +0.435 V vs SCE in DMSO[9] (the potential of Saturated Calomel Electrode (SCE) is +0.241 V vs NHE). A Heka PG310USB (Lambrecht, Germany) potentiostat with a PotMaster 2.73 software package served for the potential control in voltammetry studies. In situ UV-visible-near infrared (UV–vis–NIR) spectroelectrochemical measurements were performed on a spectrometer Avantes (Model AvaSpec-2048x14-USB2) in the spectroelectrochemical cell kit (AKSTCKIT3) with the Pt-microstructured honeycomb working electrode (Pine Research Instrumentation). The cell was positioned in the CUV–UV Cuvette Holder (Ocean Optics) connected to the diode-array UV–vis–NIR spectrometer by optical fibres. UV–vis–NIR spectra were processed using the AvaSoft 7.7 software package. Halogen and deuterium lamps were used as light sources (Avantes, Model AvaLight-DH-S-BAL). The *in situ* EPR spectroelectrochemical experiments were carried out under argon atmosphere in the EPR flat cell equipped with a large platinum mesh working electrode. The freshly prepared solutions were carefully purged with argon and the electrolytic cell was polarised in the galvanostatic mode directly in the cylindrical EPR cavity TM-110 (ER 4103

TM) and the EPR spectra were measured *in situ*. The EPR spectra were recorded at room temperature with the EMX Bruker spectrometer (Germany).

2.5. *In vitro* cell studies

2.5.1. Cell lines and culture conditions

All cell culture reagents were obtained from Sigma-Aldrich and plastic ware from Sarstedt (Germany). Doxorubicin-sensitive human colonic Colo 205 and multidrug resistant Colo 320/MDR-LRP adenocarcinoma cell lines were purchased from LGC Promochem, Teddington, UK. The cells were cultured in Roswell Park Memorial Institute (RPMI) 1640 medium supplemented with 10% heat-inactivated fetal bovine serum, 2 mM L-glutamine, 1 mM Na-pyruvate and 100 mM Hepes. The cell lines were incubated at 37°C, in a 5% CO_2, 95% air atmosphere.

MRC-5 human embryonal lung fibroblast cell line (ATCC CCL-171) was purchased from LGC Promochem, Teddington, UK. The cell line was cultured in Eagle's Minimal Essential Medium (EMEM, containing 4.5 g/L glucose) supplemented with a non-essential amino acid mixture, a selection of vitamins and 10% heat-inactivated fetal bovine serum. The cells were incubated at 37°C, in a 5% CO_2, 95% air atmosphere. All cell lines were detached with Trypsin-Versene (EDTA) solution for 5 min at 37 °C.

2.5.2. MTT assays

The tested compounds were dissolved in DMSO using 10 mM concentration. Then stock solutions were diluted in complete culture medium in 96-well microtiter plates, and two-fold serial dilutions of compounds were prepared in 100 µL of RPMI 1640, horizontally. The semi-adherent colonic adenocarcinoma cells were treated with Trypsin-Versene (EDTA) solution. They were adjusted to a density of 1×10^4 cells in 100 µL of RPMI 1640 medium, and were added to each well, with the exception of the medium control wells. The final volume of the wells containing compounds and cells was 200 µL. The culture were incubated at 37 °C for 72 h; at the end of the incubation period, 20 µL of MTT solution (from a stock solution of 5 mg/mL) were added to each well. After incubation at 37 °C for 4 h, 100 µL of SDS solution (10% in 0.01 M HCI) were added to each well and the plates were further incubated at 37 °C overnight. The cell growth was determined using both methods by measuring the optical density (OD) at 450 nm (ref. 620 nm) with a Multiscan EX ELISA reader. Inhibition of the cell growth (expressed as IC_{50}: inhibitory concentration that reduces by 50% the growth of the cells exposed to the tested compounds) was determined from

the sigmoid curve where 100 – ((OD_{sample} – $OD_{medium\ control}$)/($OD_{cell\ control}$ – $OD_{medium\ control}$)) × 100 values were plotted against the logarithm of compound concentrations. Curves were fitted by GraphPad Prism software[10] using the sigmoidal dose-response model (comparing variable and fixed slopes).

2.5.3. Mechanisms of cell death: assay for apoptosis induction

The assay was carried out for selected compounds using an Annexin V-FITC Apoptosis Detection Kit (Cat. No. APOAF-50TST) from Sigma according to the manufacturer's instructions. The concentration of the Colo320 cell suspension was adjusted to approximately 0.5 × 10^6 cells per mL in RPMI medium and the cell suspension was distributed in 1 mL aliquots into a 24-well plate, and then incubated overnight at 37 °C and 5% CO_2. On the following day, the medium was removed and replaced by 1 mL RPMI medium containing the compounds except the control samples. Colo320 cells were incubated in the presence of the compounds at 4 and 8 μM (**HL^1**), or 0.25 and 0.50 μM (**$Cu(HL^2)Cl_2$**) in the 24-well plate at 37 °C for 3 h, and 12*H*-benzo[α]phenothiazine (M627, 20 μM)[11] and cisplatin (Teva, 15 and 30 μM) were used as positive controls. After the incubation period the samples were washed with PBS and fresh RPMI medium was added to the samples. The cells were incubated overnight at 37 °C and 5% CO_2. The next day, 200 μL 0.25% Trypsin (Trypsin-Versen) was added to the samples until cells appeared detached followed by the addition of 400 μL of RPMI medium supplemented with 10% bovine serum. The cells were collected in Eppendorf tubes and centrifuged at 2000 *g* for 2 min. The harvested cells were resuspended in fresh serum free RPMI medium culture medium. After this step, the apoptosis assay was carried out according to the instructions of the manufacturer. The fluorescence was analyzed immediately using a ParTec CyFlow flow cytometer (Partec GmbH, Münster, Germany).

2.6. Tyrosyl radical reduction in mouse R2 RNR protein

The tyrosyl radical reduction in mR2 protein by **HL^1**, **HL^2**, **HL^3**, **$Cu(HL^1)Cl_2$**, **$[Cu(L^2)Cl]$**, **$Cu(HL^3)Cl_2$**, **$HL^{1a'}$**, **$HL^{1a''}$**, and **$HL^{2c'}\cdot CH_3COOH$** was measured by EPR spectroscopy at 30 K, on a Bruker Elexsys II E540 EPR spectrometer with an Oxford Instruments ER 4112HV helium cryostat, as described previously.[12,13] mR2 protein was expressed, purified, and iron-reconstituted as described previously,[14] and passed through a 5 mL HiTrap desalting column (GE Healthcare) to remove excess iron. The purified, iron-reconstituted mR2 protein resulted in the formation of 0.76 tyrosyl radical/polypeptide. Samples containing 20 µM mR2 in 50 mM HEPES buffer, pH 7.50/100 mM NaCl, and 20 µM respective compound in 1% (v/v) DMSO/H_2O, and 2 mM

dithiothreitol (DTT) were incubated for indicated times and quickly frozen in cold isopentane. The same samples were used for repeated incubations at room temperature. The experiments were performed in duplicates.

2.7. Computational details

The geometry optimizations of all key stationary points were carried out at the B3LYP level of theory[15] using Gaussian 16 suite[16] of quantum chemical programs. Pople's basis sets, 6-311++G(d,p) from Gaussian library[16] for all atoms were employed for geometry optimization. The effect of continuum solvation was incorporated by using the polarizable continuum model (PCM). Stability of the optimized structures was tested by vibrational analysis (no imaginary vibrations). Enthalpies and Gibbs free energies were obtained by adding unscaled zero-point vibrational energy corrections (ZPVE) and thermal contributions to the energies (temperature 298.150 Kelvin, pressure 1.000 atm). All transition states were fully optimized and characterized as a first-order saddle point by harmonic vibrational frequency analysis. One and only one imaginary frequency of the first-order saddle point was subjected to visual inspection to examine whether it represented the desired reaction coordinate. Furthermore, the intrinsic reaction coordinate (IRC) analysis was performed to authenticate that the transition state pertains to the desired reaction coordinate. The IRC calculations were done at the B3LYP/6-311++G(d,p) level of theory. Molecular orbitals were drawn using Molekel software.[17]

3. Schemes for assignment of resonances in HL^1 – HL^3 and their isomers

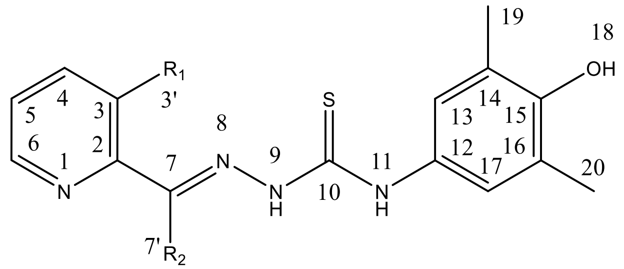

Scheme S1. The atom numbering for the assignment of resonances in $\mathbf{HL^1}$ – $\mathbf{HL^3}$.

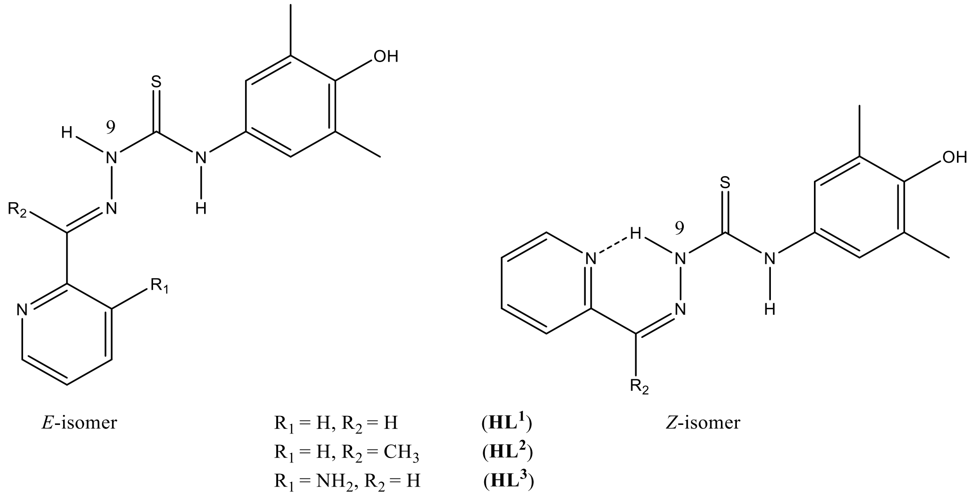

Scheme S2. The most stable conformers of $\mathbf{HL^1}$ – $\mathbf{HL^3}$ in solution.

4. Oxidative cyclization in the presence of Ag_2O

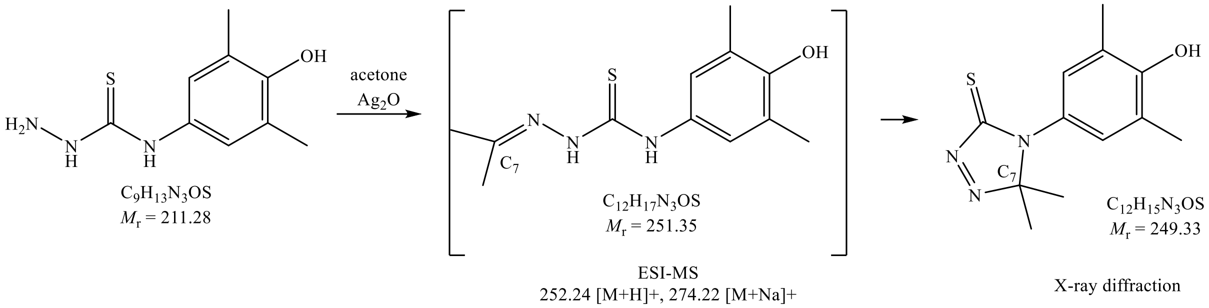

Scheme S3. The formation of acetone thiosemicarbazone in solution and its oxidative cyclization in the presence of Ag_2O.

Oxidation of $\mathbf{HL^1}$ with Ag_2O (at the molar ratio $\mathbf{HL^1}$/Ag_2O 1:1 at room temperature in acetone for 1–2 h) afforded minor amounts of 1,3,4-thiadiazoles $\mathbf{H}\boldsymbol{L}^{\mathbf{1a}'}$ and $\mathbf{H}\boldsymbol{L}^{\mathbf{1a}''}$. The reaction mainly led to complex formation of $\mathbf{HL^1}$ with Ag(I) (70–90%). This was confirmed by ESI mass spectra, in which peaks at *m*/*z* 407.15, 709.20 attributed to $[Ag(HL^1)]^+$ and $[Ag(HL^1)_2]^+$, respectively, as well as at *m*/*z* 402.73, 706.98 assigned to $[Ag(HL^{1a'})–2H]^–$, $[Ag(HL^1)_2–2H]^–$ were found. By applying the same reaction conditions (Ag_2O in acetone) to the starting thiosemicarbazide, *N*-(4-hydroxy-3,5-dimethylphenyl)hydrazinecarbothioamide (Scheme S3 in Supporting Information), the 1,2,4-triazole-3-thione ring formation as reported previously for $\mathbf{H}\boldsymbol{L}^{\mathbf{2b}}$ was confirmed by SC-XRD (Figure S2), along with coordination of the oxidized product to Ag(I) by ESI-MS.

5. Schemes for assignment of resonances in H$L^{1a'}$, H$L^{1a''}$, H$L^{2c'}$, H$L^{2c''}$ and their isomers

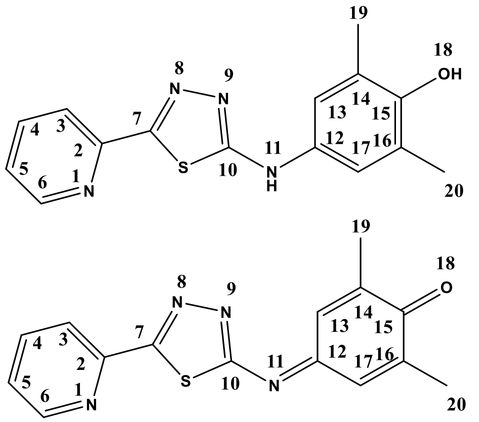

H$L^{1a'}$

H$L^{1a''}$

Scheme S4. The atom numbering for the assignment of resonances in **H*L*$^{1a'}$** and **H*L*$^{1a''}$**.

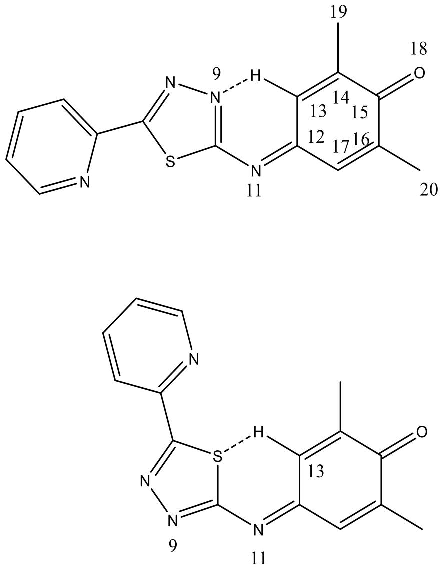

Scheme S5. The possible hydrogen bond formations of H_{13} to *N*- or *S*- of 1,3,4-thiadiazole ring in **H*L*$^{1a''}$**.

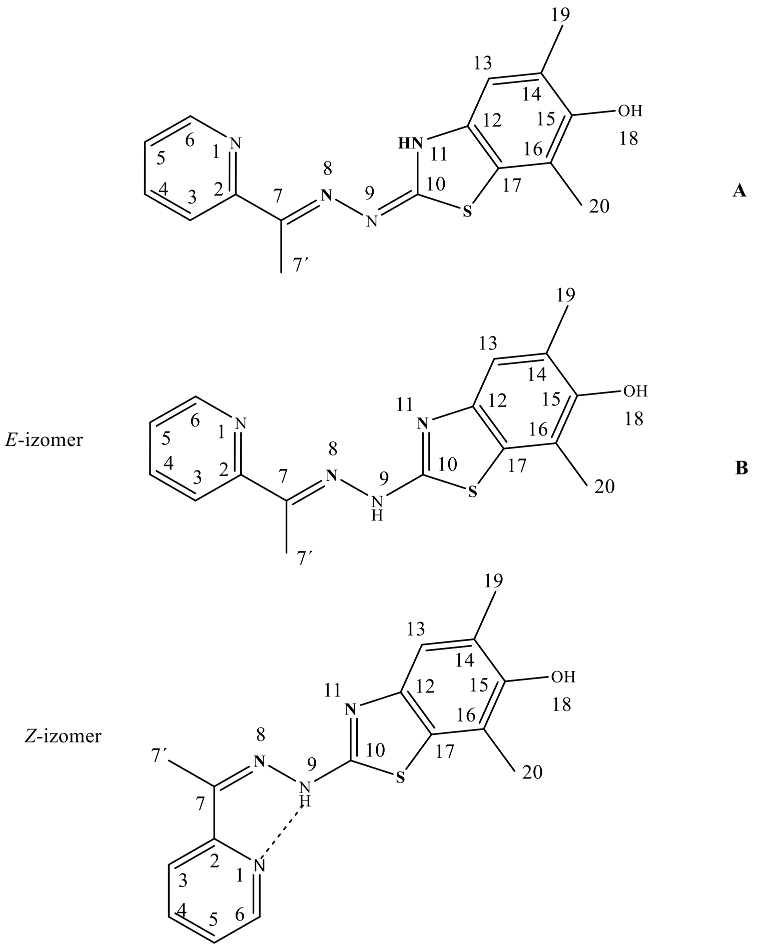

Scheme S6. The atom numbering for the assignment of resonances in **H*L*$^{\mathbf{2c'}}$** and tautomeric forms of **H*L*$^{\mathbf{2c'}}$** in solution.

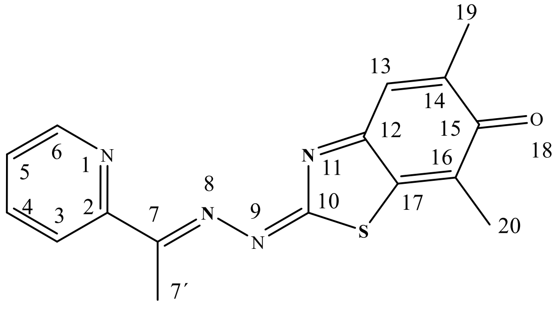

Scheme S7. The atom numbering for the assignment of resonances in **H*L*$^{\mathbf{2c''}}$**.

6. Oxidation of HL^2 with PBQ

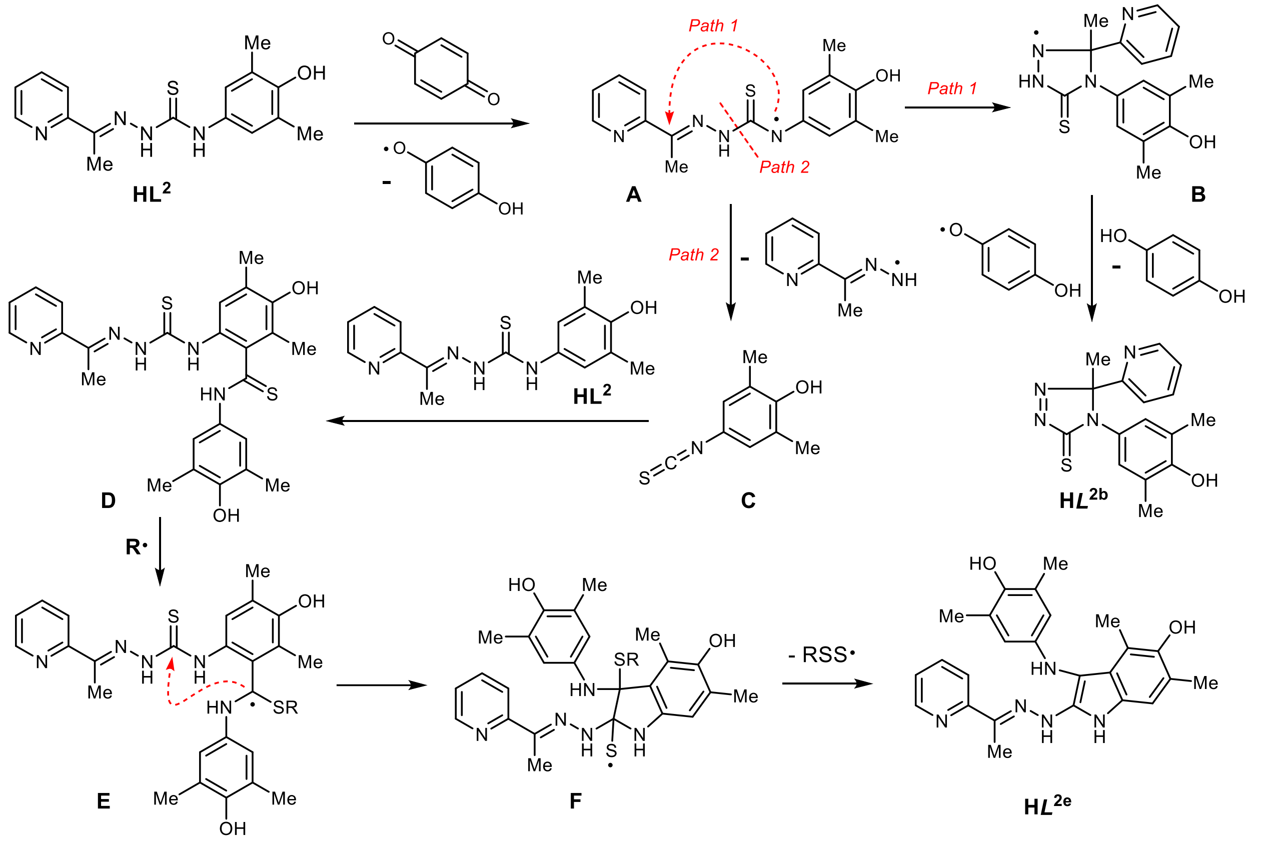

Scheme S8. Plausible pathway for the formation of compounds **H*L*^{2b}** and **H*L*^{2e}** by the oxidation of **HL^2** with PBQ.

Discussion of Scheme S8. We suppose that the initial step of the reaction of **HL^2** with PBQ involves one-electron oxidation of **HL^2** together with NH deprotonation to give a highly conjugated N/S-centered free radical species **A**. Intramolecular addition of the N4 atom in radical species **A** to the C=N double bond results in the triazole-based radical **B** which is then oxidized and deprotonated to form **H*L*^{2b}** (*Path 1*). Another transformation of radical **A** involves its fragmentation affording isothiocyanate **C** (*Path 2*). This path is supported by the literature data describing isothiocyanate syntheses by oxidation of thiosemicarbazides.[18,19] It should be also noted that formation of intermediate species **C** was observed upon oxidation of N-(4-hydroxy-3,5-dimethylphenyl)hydrazinecarbothioamide or **ATSC´** (Ag_2O, acetone), of HL^1 (Ag_2O, acetone; PBQ in ethanol), DDQ in methanol) and confirmed by the negative ion ESI mass spectra showing a peak with *m*/*z* value 177.95 attributed to $[M–H]^–$. Moreover this species was separated by column chromatography on silica when using ethyl acetate/hexane (v/v 1/1, R_f = 0.85). Electrophilic substitution in highly activated benzene ring of **HL^2** under the action of isothiocyanate **C** gives the

corresponding thioamide **D**. Analogous S_E2 reactions have been described in the literature.[20] The final steps of indole **H*L*^{2e}** formation proceed presumably according to radical promoted Fukuyama-like indole synthesis.[21,22] Thus, the attack of a radical on the thioamide sulfur atom affords radical **E** followed by its cyclization into **F** and then aromatization to afford **H*L*^{2e}**.

7. Suggested structures for [Cu(L^1)(H$_2$O)]$^+$ and [Cu(HL^1)(H$_2$O)]$^{2+}$

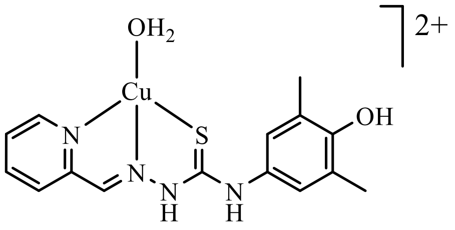

[Cu((HL^1)(H$_2$O)]$^{2+}$

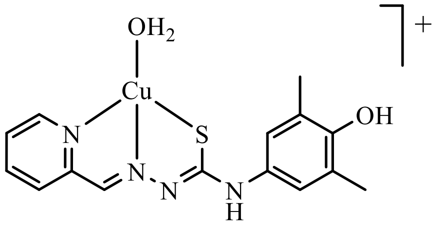

[Cu((L^1)(H$_2$O)]$^+$

Chart S1. Suggested structures for **[Cu(L^1)(H$_2$O)]$^+$** and **[Cu(HL^1)(H$_2$O)]$^{2+}$** complexes. (Notably, the chloride ions originally coordinated in solid phase are most likely replaced by water molecules due to the weak binding of the chlorido ligands in aqueous solution).

8. Frontier orbitals in HL^2

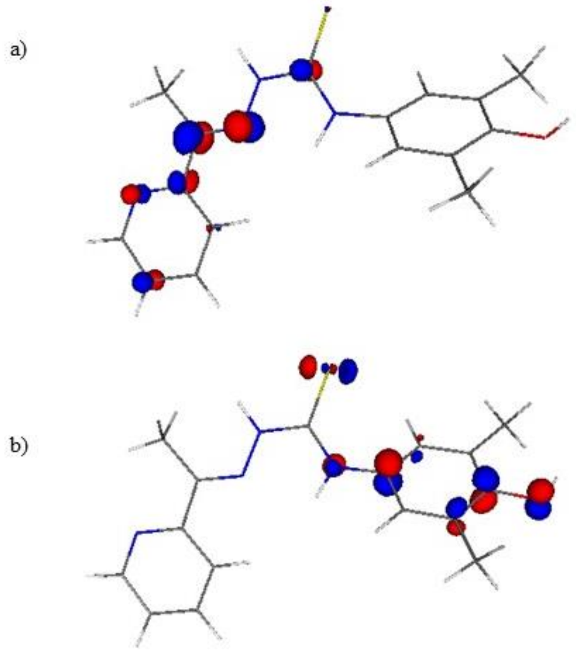

Figure S1. Frontier orbitals in $\mathbf{HL^2}$: a) LUMO and b) HOMO.

9. ORTEP view of the ATSC

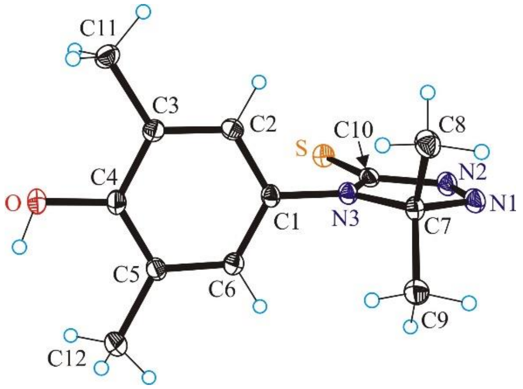

Figure S2. ORTEP view of the oxidation product of acetone *N*-(4-hydroxy-3,5-dimethylphenylthiosemicarbazone (ATSC) by Ag_2O.

10. *E*/*Z*-isomerization of H*L*$^{2c'}$

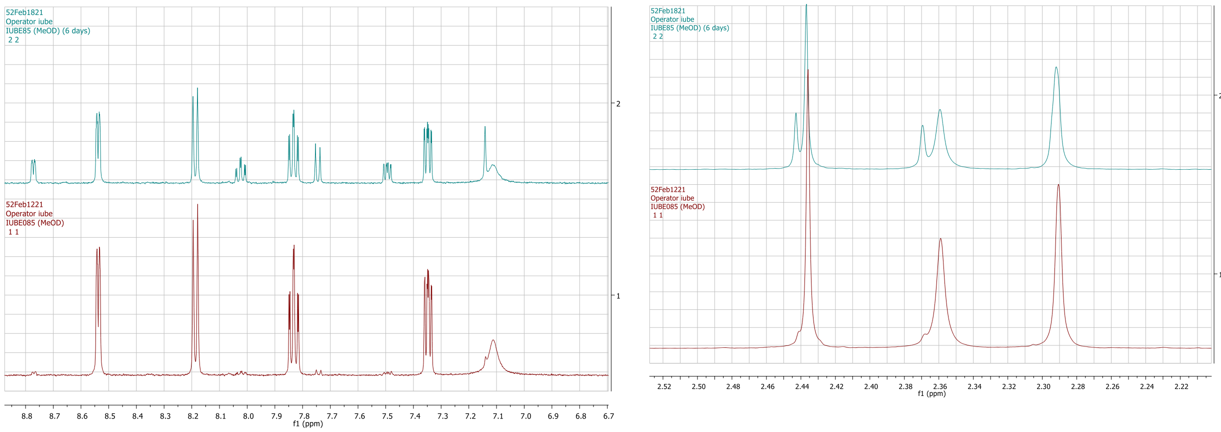

Figure S3. *E*/*Z*-isomerization of **H*L*$^{2c'}$** in MeOH-d_4 in 6 days (red spectra – fresh (*E*-), blue spectra – in 6 days (*E*-/*Z*- as 3/1)).

-^{1}H NMR (**H*L*$^{2c'}$**, 500 MHz, MeOH-d_4) δ 8.77 (ddd, *J* = 4.9, 1.7, 0.8 Hz, 1H, *Z*-isomer), 8.54 (ddd, *J* = 4.9, 1.6, 0.9 Hz, 3H, *E*-isomer), 8.19 (d, *J* = 8.1 Hz, 3H, *E*-isomer), 8.02 (td, *J* = 7.9, 1.8 Hz, 1H, *Z*-isomer), 7.83 (td, *J* = 7.8, 1.8 Hz, 3H, *E*-isomer), 7.75 (d, *J* = 8.1 Hz, 1H, *Z*-isomer), 7.49 (ddd, *J* = 7.6, 4.9, 0.9 Hz, 1H, *Z*-isomer), 7.35 (ddd, *J* = 7.4, 4.9, 1.1 Hz, 3H, *E*-isomer), 7.14 (s, 1H, *Z*-isomer), 7.11 (s, 3H, *E*-isomer), 2.44 (s, 3H, *Z*-isomer), 2.43 (s, 9H, *E*-isomer), 2.37 (s, 3H, *Z*-isomer), 2.36 (s, 9H, *E*-isomer), 2.29 (s, 3H+9H, *Z*-isomer and *E*-isomer).

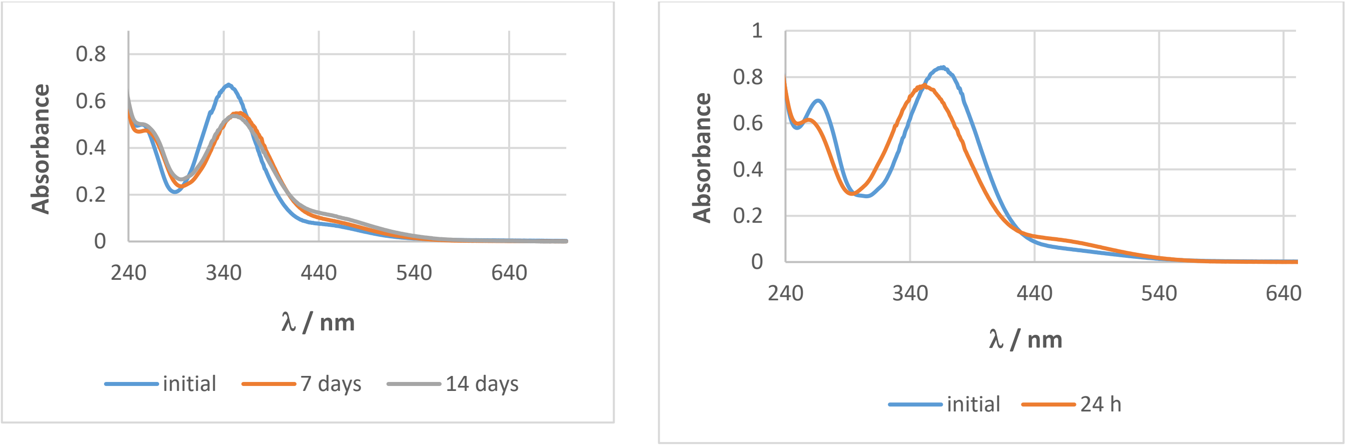

E-isomer *Z*-isomer

Figure S4. *E*/*Z*-isomerization of **H*L*$^{2c'}$** in MeOH investigated by UV–vis spectroscopy.

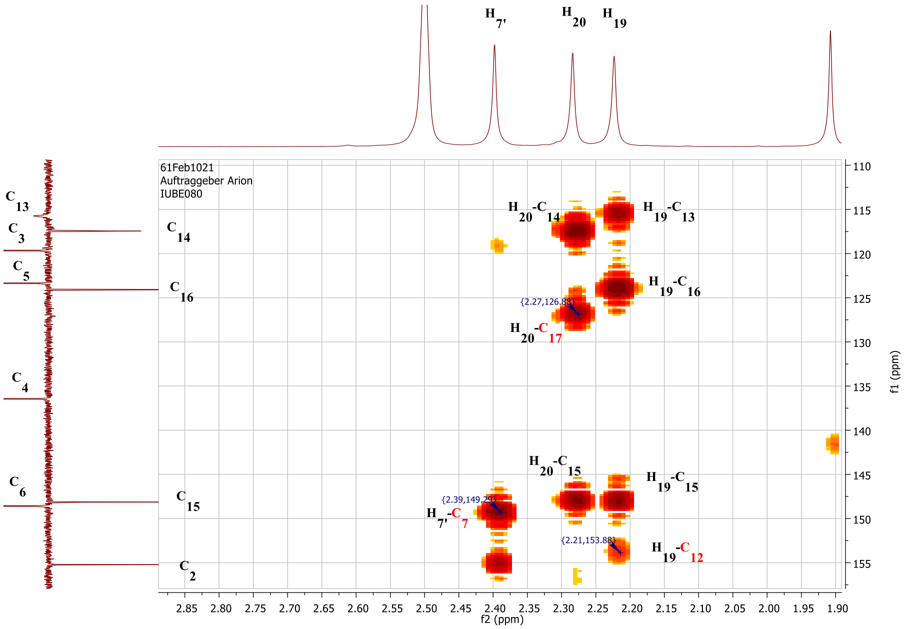

Figure S5. The fragment of $^{1}H,^{13}C$ HMBC of **H*L*$^{2c'}$·CH$_3$COOH** (to assign carbons C_{12}, C_7 and C_{17}).

11. ORTEP view of H*L*^{2e} and [Cu(*L*$^{1c´}$)Cl] (4)

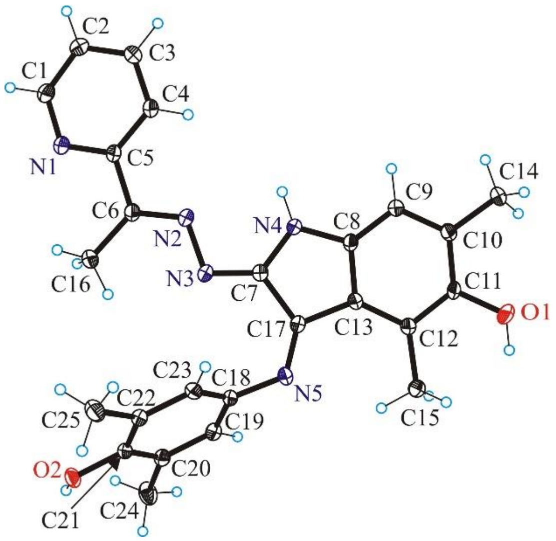

Figure S6. ORTEP view of **H*L*^{2e}** with thermal displacement parameters at 50% probability level. Selected bond distances (Å) and bond angles (°): C6–N2 1.2908(13), N2–N3 1.3999(12), N3–C7 1.2987(13), C7–N4 1.3656(12), N4–C8 1.3969(12), C8–C13 1.4006(14), C13–C17 1.4763(13), C17–C7 1.5149(14), C11–O1 1.3812(12), C17–N5 1.2861(13), N5–C18 1.4150(12), C21–O2 1.3729(11).

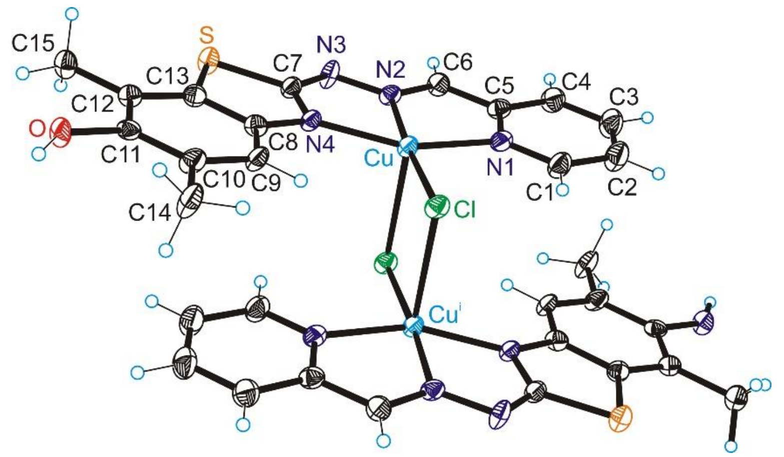

Figure S7. ORTEP view of centrosymmetric dimer in **[Cu(*L*[1c´])Cl]** (**4**) with thermal ellipsoids at 50% probability level. Bond lengths to bridging µ-chlorido ligands: Cu–Cl = 2.2575(12) Å, Cu–Cl^{i} = 2.7633(13) Å; (i denotes equivalent atom generated by symmetry transformation –*x* + 1, –*y* + 1, –*z* + 1.

12. Spectroscopic data

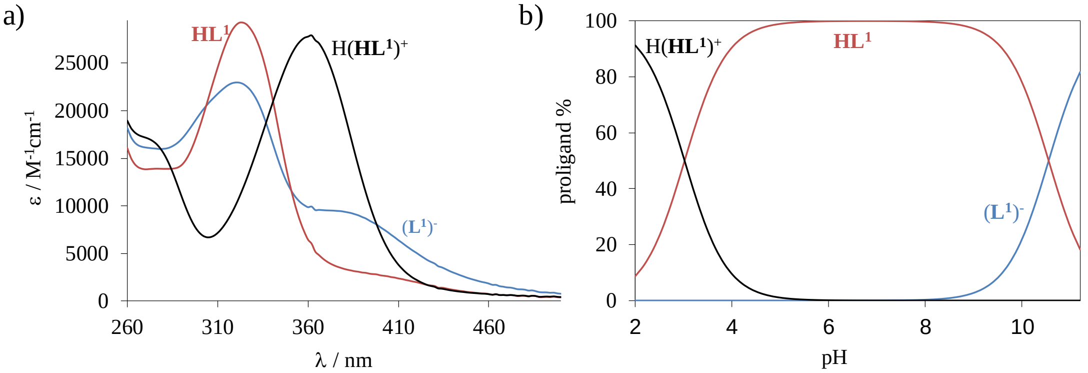

Figure S8. a) Molar absorbance spectra of the metal-free ligand $\mathbf{HL^1}$ in different protonation states. b) Concentration distribution curves for H($\mathbf{HL^1}$)$^{+}$, $\mathbf{HL^1}$ and ($\mathbf{L^1}$)$^{-}$. {c_{HL} = 50 µM; 30% (v/v) DMSO/H_2O; *I* = 0.1 M (KCl); *T* = 25 °C}

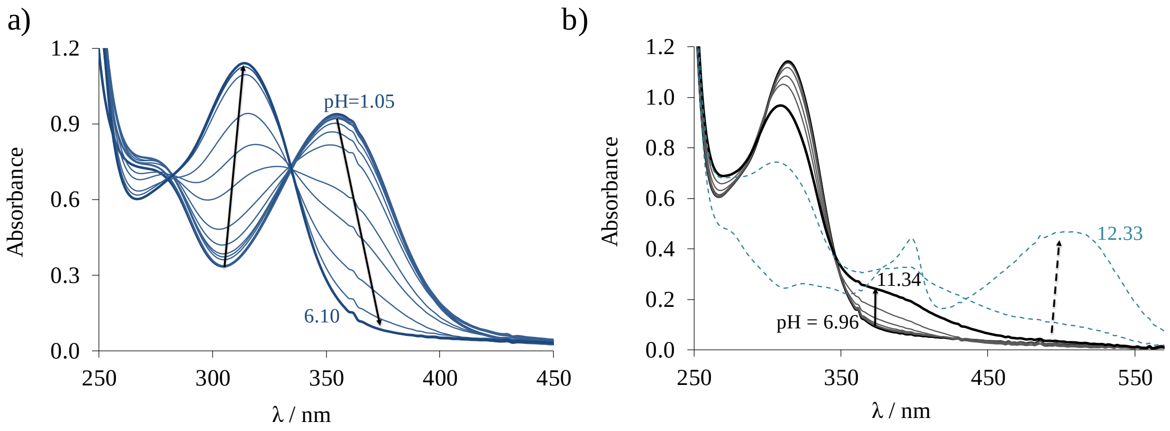

Figure S9. UV–vis absorption spectra recorded for proligand $\mathbf{HL^2}$ in the pH range a) 1.05 – 6.10 and b) 6.96 – 12.33. {$c_{\mathbf{HL}}$ = 50 µM; 30% (v/v) DMSO/H_2O; I = 0.1 M (KCl); T = 25 °C}.

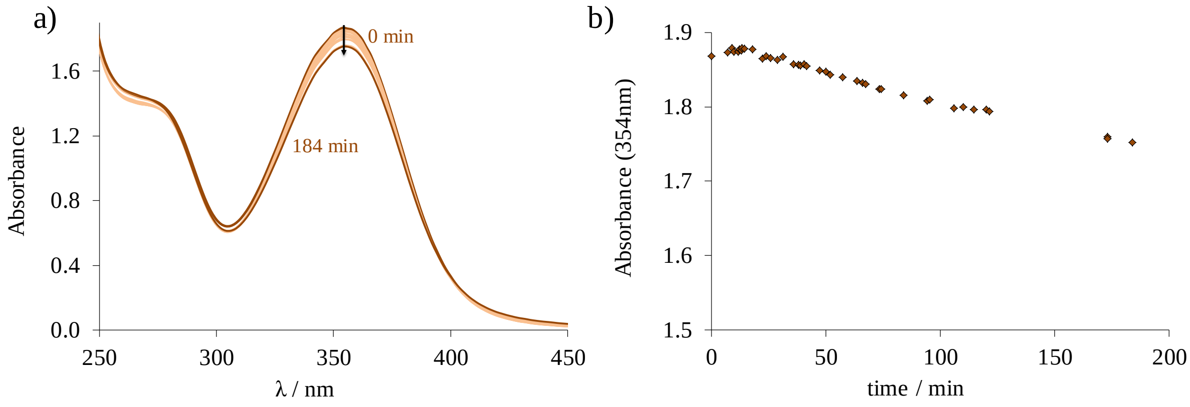

Figure S10. a) UV–vis absorption spectra recorded for proligand $\mathbf{HL^2}$ at pH 1.5 over 3 h. b) Absorbance values at 354 nm plotted against the time. {$c_{\mathbf{HL}}$ = 100 µM; 30% (v/v) DMSO/H_2O; I = 0.1 M (KCl); T = 25 °C}.

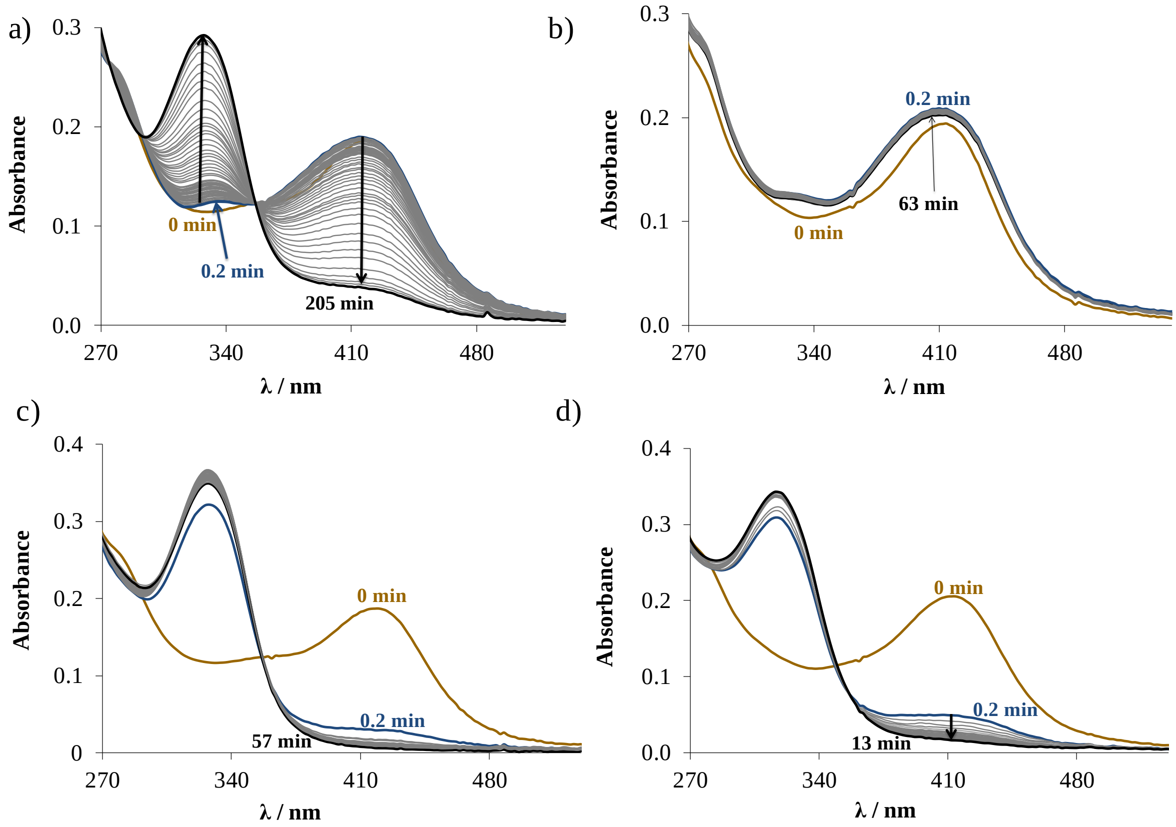

Figure S11. Time dependent changes of the UV–vis spectra of a) **Cu(HL[1])Cl$_2$** and b) **Cu(L[2])Cl** in the presence of 50 equiv GSH, c) **Cu(HL[1])Cl$_2$** and d) **Cu(L[2])Cl** in the presence of 50 equiv DTT, at pH 7.4 in 60% (v/v) DMSO/H_2O under anaerobic conditions {$c_{complex}$ = 12.5 µM; $c_{GSH\ or\ DTT}$ = 600 µM; pH = 7.40; I = 0.1 M (KCl); T = 25 °C}.

13. Spectroelectrochemical data

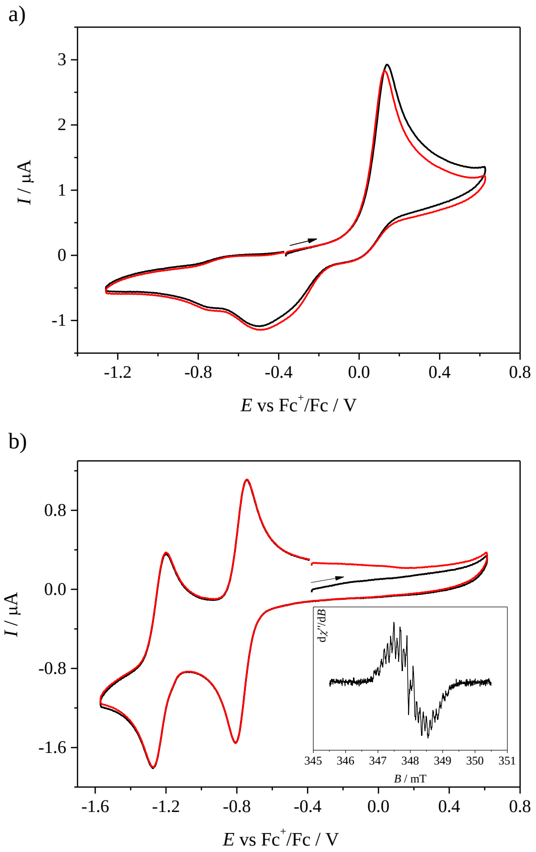

Figure S12. Cyclic voltammograms of a) 0.5 mM **H*L***$^{\mathbf{1a'}}$ and b) **H*L***$^{\mathbf{1a''}}$ in DMSO/*n*-Bu_4NPF_6 at glassy-carbon working electrode at scan rate of 100 mV s^{-1}. Inset in b): EPR spectrum measured upon cathodic reduction of **H*L***$^{\mathbf{1a''}}$ at the first reduction peak.

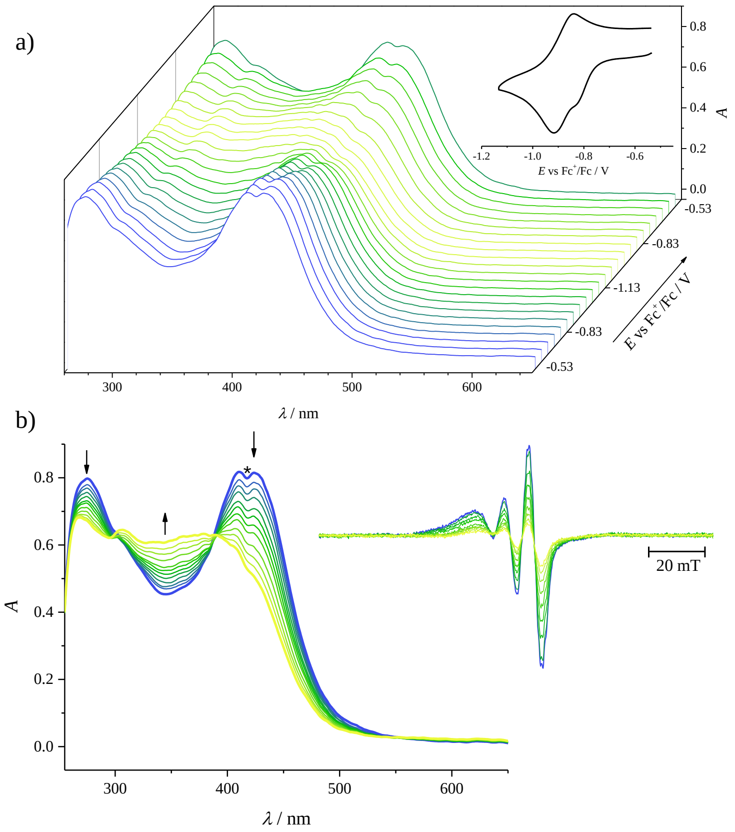

Figure S13. Spectroelectrochemistry of **2´** in *n*-Bu_4NPF_6/DMSO in the region of the first cathodic peak. a) Potential dependence of UV–vis spectra with the corresponding in situ cyclic voltammogram (Pt-microstructured honeycomb working electrode, scan rate of 5 mV s^{-1}); b) evolution of UV–vis spectra in 2D projection upon forward scan (* the peak marked by an asterisk is due to an artefact caused by the instrument; inset: evolution of EPR spectra measured at the first reduction peak using Pt mesh working electrode).

14. EPR measurement

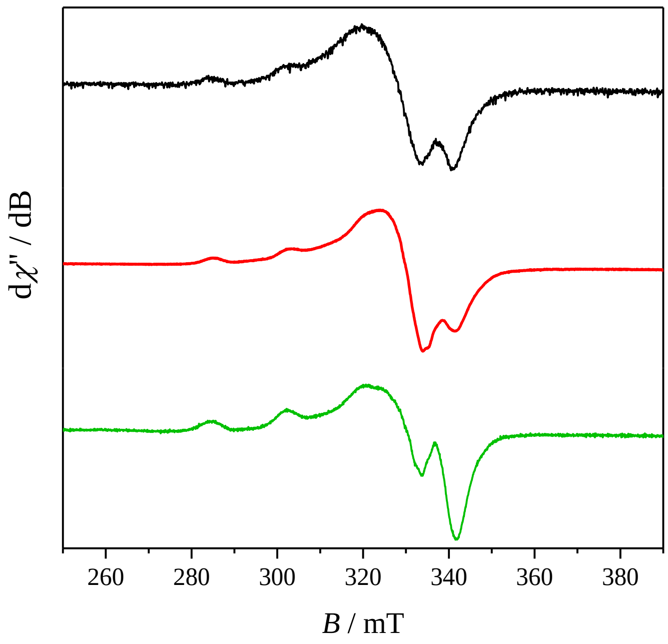

Figure S14. EPR spectra of **1** (black trace), **2´** (red trace) and **3** (green trace) measured in frozen *n*-Bu_4NPF_6/DMSO at 77 K.

15. Tyrosyl radical reduction kinetics in mouse R2 RNR protein

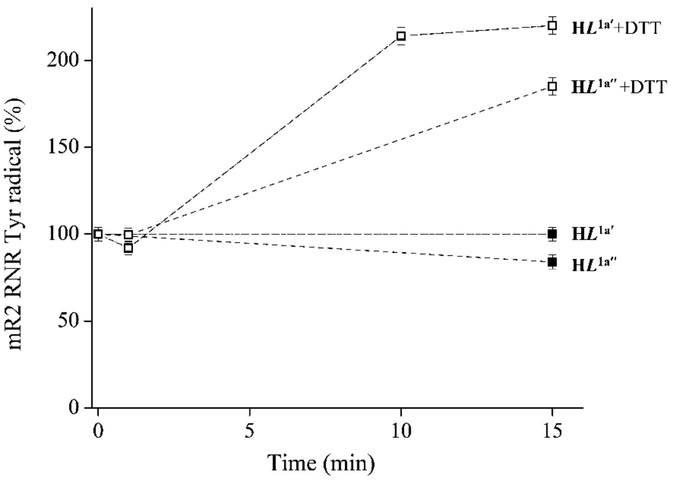

Figure S15. Tyrosyl radical reduction kinetics in mouse R2 RNR protein by **H*L*$^{1a'}$** and **H*L*$^{1a''}$**, in absence and presence of an external reductant, measured by 30 K EPR spectroscopy. The samples contained 20 µM mR2 in 50 mM Hepes buffer, pH 7.60 / 100 mM KCl, 20 µM compound in 1% (v/v) DMSO/H_2O, and 2 mM dithiothreiotol (DTT).

16. Cell cycle arrest

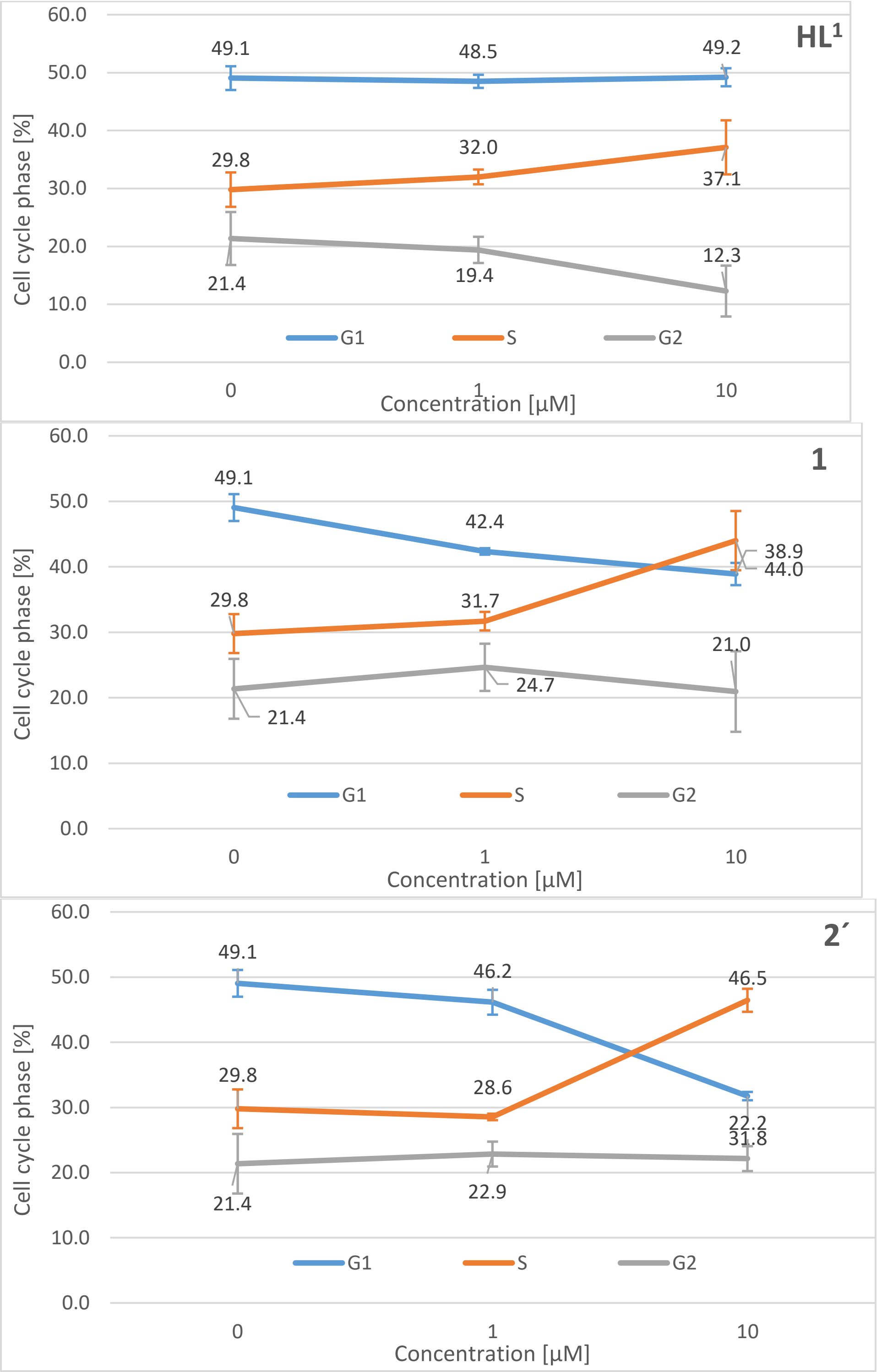

Figure S16. Cell cycle distribution of SW480 cells induced by increased concentrations of **HL**[1] (top) and complexes **1** (middle) and **2´**(bottom) for 24 h compared to negative control (DMSO).

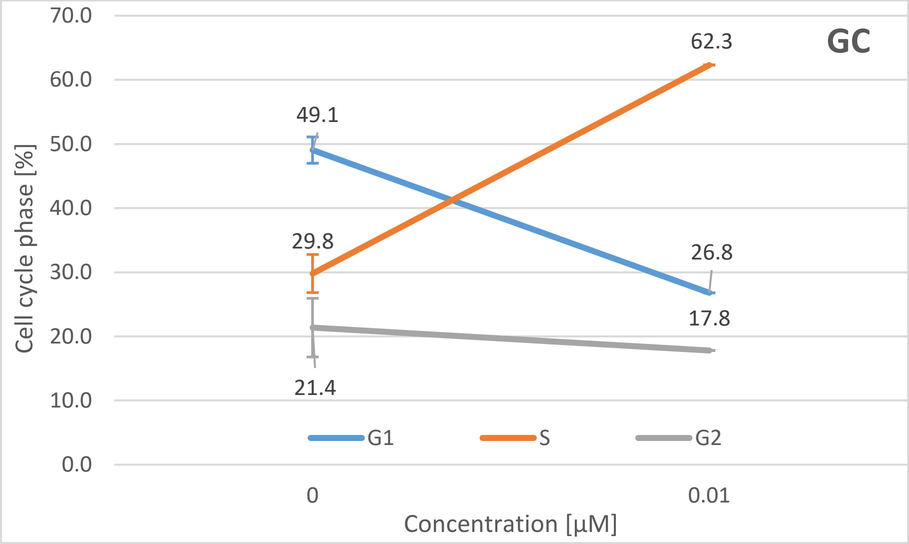

Figure S17. Cell cycle distribution of SW480 cells induced by increased concentrations of Gemcitabine (GC) for 24 h compared to negative control (DMSO).

17. Crystal Data and Details of Data Collection

Table S1. Crystal Data and Details of Data Collection for **HL¹**·C_2H_5OH, **HL²** and **HL³**

Compound	**HL¹**·C_2H_5OH	**HL²**	**HL³**
empirical formula	$C_{17}H_{22}N_4O_2S$	$C_{16}H_{18}N_4OS$	$C_{15}H_{17}N_5OS$
fw	346.45	314.40	315.40
space group	triclinic, $P\bar{1}$	monoclinic, $P2_1/c$	monoclinic, $P2_1/c$
a, Å	8.0232 (4)	7.4870 (11)	14.7916 (7)
b, Å	10.4578 (6)	8.6775 (15)	13.1867 (7)
c, Å	11.2363 (5)	24.709 (5)	15.9256 (8)
α,°	76.4523 (19)	90	90
β, °	74.485 (3)	90.387 (6)	105.1976 (19)
γ, °	82.121 (2)	90	90
V [Å^3]	880.36 (8)	1605.3 (5)	2997.7 (3)
Z	2	4	8
λ [Å]	0.71073	0.71073	0.71073
ρ_{calcd}, g cm^{-3}	1.307	1.301	1.398
cryst size, mm^3	0.10 × 0.09 × 0.04	0.10 × 0.08 × 0.01	0.20 × 0.10 × 0.08
T [K]	110(2)	120(2)	120(2)
μ, mm^{-1}	0.201	0.209	0.226
R_1[a]	0.0347	0.0570	0.0562
wR_2[b]	0.0827	0.1871	0.1526
GOF[c]	1.047	1.072	1.008

[a] $R_1 = \Sigma||F_o| - |F_c||/\Sigma|F_o|$. [b] $wR_2 = \{\Sigma[w(F_o^2 - F_c^2)^2]/\Sigma[w(F_o^2)^2]\}^{1/2}$. [c] GOF = $\{\Sigma[w(F_o^2 - F_c^2)^2]/(n - p)\}^{1/2}$, where n is the number of reflections and p is the total number of parameters refined .

Table S2. Crystal Data and Details of Data Collection for **H*L*$^{1a'}$**, **H*L*$^{1a''}$**, **H*L*^{2b}**, **H*L*^{2e}** and **ATSC′**

Compound	**H*L*$^{1a'}$**	**H*L*$^{1a''}$**	**H*L*^{2b}**	**HL$^{2c''}$·0.5CHCl$_3$**	**H*L*^{2e}**	**ATSC′**
empirical formula	$C_{15}H_{14}N_4OS$	$C_{15}H_{12}N_4OS$	$C_{16}H_{18}N_4OS$	$C_{16.5}H_{14.5}Cl_{1.5}N_4OS$	$C_{25}H_{25}N_5O_2$	$C_{12}H_{15}N_3OS$
fw	298.36	296.35	314.40	370.06	427.50	249.33
space group	monoclinic, $P2_1/n$	monoclinic, *Cc*	monoclinic, $P2_1/c$	triclinic, *P*-1	triclinic, $P\bar{1}$	monoclinic, $P2_1/n$
a, Å	13.7285(3)	22.449(2)	7.5533(4)	8.5138(3)	9.8871(7)	12.3755(3)
b, Å	4.8713(1)	3.8288(4)	20.6597(10)	11.0999(5)	10.8202(5)	7.2202(2)
c, Å	20.8797(4)	16.289(3)	9.5891(7)	17.9548(6)	11.9861(5)	13.9172(4)
α,°				85.5846(15)	116.4987(18)	
β, °	106.100(1)	100.513(6)	91.511(3)	87.0782(14)	92.850(2)	91.824(1)
γ, °				89.039(3)	106.217(3)	
V [Å^3]	1341.58(5)	1376.5(3)	1495.85(15)	1689.39(11)	1078.99(10)	1242.92(6)
Z	4	4	4	4	2	4
λ [Å]	0.71073	0.71073	0.71073	0.71073	0.71073	0.71073
ρ_{calcd}, g cm^{-3}	1.477	1.430	1.387	1.455	1.316	1.332
cryst size, mm^3	0.35 × 0.23 × 0.01	0.09 × 0.06 × 0.03	0.14 × 0.13 × 0.08	0.20 × 0.15 × 0.01	0.23 × 0.20 × 0.09	0.62 × 0.55 ×0.13
T [K]	100(2)	100(2)	100(2)	125(2)	100(2)	100(2)
μ, mm^{-1}	0.246	0.239	0.224	0.440	0.086	0.248
$R_1{}^a$	0.0355	0.0314	0.0466	0.0581	0.0456	0.0323
$wR_2{}^b$	0.0932	0.0724	0.1174	0.1653	0.1360	0.0843
GOF^c	1.090	1.026	1.055	1.069	1.046	1.064

^{a} $R_1 = \Sigma||F_o| - |F_c||/\Sigma|F_o|$. ^{b} $wR_2 = \{\Sigma[w(F_o^2 - F_c^2)^2]/\Sigma[w(F_o^2)^2]\}^{1/2}$. ^{c} GOF $= \{\Sigma[w(F_o^2 - F_c^2)^2]/(n - p)\}^{1/2}$, where n is the number of reflections and p is the total number of parameters refined .

Table S3. Crystal Data and Details of Data Collection for **1′**·CH_3OH, **2′**, **3′**·CH_3OH, **4–6**

Compound	**1′**·CH_3OH	**2′**	**3′**·CH_3OH	**4**	**5**	**6**
empirical formula	$C_{16}H_{19}N_4O_2SCuCl$	$C_{16}H_{17}N_4OSCuCl$	$C_{16}H_{20}N_5O_2SCuCl$	$C_{15}H_{13}N_4OSCuCl$	$C_{14}H_{16}Cl_2CuN_8O_2S$	$C_{17}H_{20}Cl_2CuN_4O_2S$
fw	430.40	412.39	445.42	396.34	526.91	478.87
space group	monoclinic, $P2_1/c$	triclinic, $P\bar{1}$	monoclinic, $P2_1/c$	monoclinic, $P2_1/c$	monoclinic, $P2_1/n$	triclinic, $P\bar{1}$
a, Å	14.0310(5)	7.3880(15)	13.9941 (10)	8.8847(16)	9.8513(2)	8.4395(5)
b, Å	6.7988(2)	8.6970(17)	9.6773 (4)	18.122(2)	8.12370(10)	10.9171(13)
c, Å	18.4126(6)	13.884(3)	14.0005 (9)	9.2690(17)	12.6408(2)	11.1165(7)
α, °		84.84(3)				79.343(4)
β, °	91.286(3)	87.45(3)	103.980 (3)	92.814(6)	101.7347(8)	86.602(2)
γ, °		68.64(3)				71.706(4)
V [Å^3]	1756.01(10)	827.4(3)	1839.86 (19)	1490.5(4)	990.49(3)	955.68(14)
Z	4	2	4	4	2	2
λ [Å]	0.71073	0.70000	0.71073	0.71073	0.71073	0.71073
ρ_{calcd}, g cm^{-3}	1.628	1.655	1.608	1.766	1.767	1.664
cryst size, mm^3	0.12 × 0.12 × 0.03	0.05 × 0.02 × 0.01	0.09 × 0.09 × 0.02	0.15 × 0.03 × 0.02	0.19 × 0.13 × 0.08	0.06 × 0.05 × 0.04
T [K]	120(2)	100(2)	120(2)	100(2)	100(2)	100(2)
μ, mm^{-1}	1.533	1.556	1.467	1.793	1.614	1.553
$R_1{}^a$	0.0369	0.0659	0.0348	0.0477	0.0238	0.0283
$wR_2{}^b$	0.0951	0.1999	0.0802	0.1116	0.0599	0.0668
GOF^c	1.089	1.060	1.054	1.054	1.078	1.058

^{a} $R_1 = \Sigma||F_o| - |F_c||/\Sigma|F_o|$. ^{b} $wR_2 = \{\Sigma[w(F_o{}^2 - F_c{}^2)^2]/\Sigma[w(F_o{}^2)^2]\}^{1/2}$. ^{c} GOF = $\{\Sigma[w(F_o{}^2 - F_c{}^2)^2]/(n - p)\}^{1/2}$, where n is the number of reflections and p is the total number of parameters refined.

18. ^{1}H and ^{13}C NMR Data

Table S4. ^{1}H NMR (DMSO-d_6)

		**HL^1** (*E*-)	**HL^2** (*E*-)	**HL^3** (*E*-)	**$HL^{1a'}$**	**$HL^{1a''}$**	**$HL^{2c'}$**·CH_3COOH (*E*-)	**$HL^{2c'}$** (*E*-)	**$HL^{2c''}$** ($CDCl_3$)
H_3	CH_{py}	8.43 (d, 1H)	8.54 (d, 1H)	-	8.11 (d, 1H)	8.26 (dt, 1H)	8.08 (d, 1H)	8.07 (d, 1H)	8.34 (d, 1H)
$H_{3'}$	NH_2	-	-	6.49 (s, 2H)	-	-	-	-	-
H_4	CH_{py}	7.82 (td, 1H)	7.79 (td, 1H)	7.15 (dd, 1H)	7.95 (td, 1H)	8.06 (td, 1H)	7.85 (td, 1H)	7.85 (td, 1H)	7.80 (td, 1H)
H_5	CH_{py}	7.37 (m, 1H)	7.39 (dd, 1H)	7.08 (dd, 1H)	7.46 (ddd, 1H)	7.60 (ddd, 1H)	7.36 (dd, 1H)	7.36 (ddd, 1H)	7.38 (dd, 1H)
H_6	CH_{py}	8.57 (d, 1H)	8.59 (d, 1H)	7.85 (dd, 1H)	8.61 (d, 1H)	8.71 (ddd, 1H)	8.58 (d, 1H)	8.58 (ddd, 1H)	8.70 (d, 1H)
H_7	CH=N	8.16 (s, 1H)	-	8.39 (s, 1H)	-	-	-	.	
$H_{7'}$	CH_3(C=N)	-	2.44 (s, 3H)	-	-	-	2.40 (s, 3H)	2.40 (s, 3H)	2.72 (s, 3H)
H_9	N*H* (closer to py)	11.86 (s, 1H)	10.46 (s, 1H)	11.47 (s, 1H)	-	-	11.76 (s, 1H)	11.58 (s, 1H)	
H_{11}	N*H* (closer to ph)	10.00 (s, 1H)	9.94 (s, 1H)	9.70 (s, 1H)	10.20 (s, 1H)	-	-	-	
H_{13}+H_{17}	CH_{ph}	7.02 (s, 2H)	7.02 (s, 2H)	6.92 (s, 2H)	7.16 (s, 2H)	7.46 (dq, 1H, H_{13})	7.02 (s, 1H, H_{13})	7.02 (brs, 1H)	7.44 (brs, 1H)
						7.20 (dq, 1H, H_{17})			
H_{18}	OH_{ph}	8.22 (s, 1H)	8.22 (s, 1H)	8.21 (s, 1H)	8.08 (s, 1H)	-	8.18 (s, 1H)	8.20 (s, 1H)	
H_{19}+H_{20}	CH_{3ph}	2.17 (s, 6H)	2.17 (s, 6H)	2.16 (s, 6H)	2.17 (s, 6H)	2.05 (d, 3H, H_{20})	2.28 (s, 3H, H_{20})	2.28 (s, 3H, H_{20})	2.18 (s, 3H, H_{19})
						2.00 (d, 3H, $H_{19)}$	2.22 (s, 3H, H_{19})	2.22 (s, 3H, H_{19})	2.14 (s, 3H, H_{20})

		**HL^1** (*Z*-)	**HL^2** (*Z*-)	**HL^3** (*Z*-)	**$HL^{2c'}$** (*Z*-)
H_3	CH_{py}	n.a.	n.a.	n.a.	7.79 (d, 1H)
$H_{3'}$	NH_2	-	-	6.21 (s, 2H)	-
H_4	CH_{py}	n.a.	n.a.	n.a.	8.08 (t, 1H)
H_5	CH_{py}	n.a.	n.a.	n.a.	7.58 (m, 1H)
H_6	CH_{py}	n.a.	n.a.	n.a.	8.85 (d, 1H)
$H_{7'}$	CH_3(C=N)	-	2.43 (s, 3H)	-	2.40 (s, 3H)
H_9	N*H* (closer to py)	14.26 (s, 1H)	14.40 (s, 1H)	14.57 (s, 1H)	15.00 (s, 1H)
H_{11}	N*H* (closer to ph)	10.17 (s, 1H)	9.99 (s, 1H)	9.95 (s, 1H)	-
H_{13}	CH_{ph}	n.a.	n.a.	n.a.	7.15 (s, 1H)
H_{18}	OH_{ph}	n.a.	n.a.	n.a.	8.22 (s,1H)
H_{19}+H_{20}	CH_{3ph}	2.16 (s, 6H)	2.16 (s, 6H)	2.15 (s, 6H)	2.31 (s, 3H, H_{20})
					2.22 (s, 3H, H_{19})

Table S5. ^{13}C NMR (DMSO-d_6)

		**HL^1** (*E*-)	**HL^2** (*E*-)	**HL^3** (*E*-)	**H*L*$^{1a'}$**	**H*L*$^{1a''}$**	**H*L*$^{2c'}$**·CH_3COOH (*E*-)	**H*L*$^{2c'}$** (*E*-)	**H*L*$^{2c''}$** ($CDCl_3$)
C_2	*C*$_{py}$	153.31	154.59	132.97	149.28	148.19	155.22	155.15	154.97
C_3	*C*H$_{py}$/*C*$_{py}$	120.54	121.18	143.99	119.25	120.04	119.65	119.65	122.31
C_4	*C*H$_{py}$	136.43	136.34	122.34	137.52	138.06	136.43	136.44	136.46
C_5	*C*H$_{py}$	124.10	124.00	124.52	124.61	126.14	123.35	123.35	125.34
C_6	*C*H$_{py}$	149.27	148.43	137.25	149.73	150.25	148.57	148.57	149.29
C_7	*C*=N	142.51	148.54	149.23	-	-	149.29*	n.a.	169.42
C_7	thiadiazole	-	-	-	158.40	169.98	-	-	-
$C_{7'}$	*C*H$_3$(C=N)	-	12.31	-	-	-	12.55	12.56	14.88
C_{10}	*C*=S	176.55	177.36	176.13	-	-	-	-	-
C_{10}	S-*C*-N	-	-	-	166.77	171.58	166.47	n.a.	173.74
C_{12}	*C*$_{ph}$	130.18	130.36	130.59	132.53	162.21	153.88*	n.a.	169.74
C_{13}+C_{17}	*C*H$_{ph}$	126.26	126.33	126.88	118.67	136.08 (C_{17})	126.88* (C_{17})	n.a.	129.52 (C_{13})
						125.96 (C_{13})	115.78 (C_{13})	n.a.	129.25 (C_{17})
C_{14}+C_{16}	*C*(CH$_3$)$_{ph}$	123.84	123.83	123.83	125.11	143.89 (C_{14})	124.07	124.07	145.86
						143.11 (C_{16})	117.44	117.80*	145.41
C_{15}	*C*(OH)$_{ph}$	151.10	151.12	151.17	148.97	187.14	148.14	148.15	184.43
C_{19}+C_{20}	C(*C*H$_3$)$_{ph}$	16.62	16.63	16.63	16.87	16.10 (C_{19})	17.08 (C_{19})	17.09 (C_{19})	17.38 (C_{19})
						15.66 (C_{20})	15.47 (C_{20})	15.54C_{20})	15.54 (C_{20})

		**HL^1** (*Z*-)	**HL^2** (*Z*-)	**HL^3** (*Z*-)	**H*L*$^{2c'}$** (*Z*-)
C_2	*C*$_{py}$				152.61
C_3	*C*H$_{py}$				124.08*
C_4	*C*H$_{py}$				138.29
C_5	*C*H$_{py}$				124.26*
C_6	*C*H$_{py}$				147.63
$C_{7'}$	*C*H$_3$(C=N)	-	21.73	-	21.72
C_{13}	*C*H$_{ph}$				118.98 (C_{13})
C_{14}+C_{16}	*C*(CH$_3$)$_{ph}$				117.67* (C_{14} or C_{16})
C_{15}	*C*(OH)$_{ph}$				148.39
C_{19}+C_{20}	C(*C*H$_3$)$_{ph}$				17.20 (C_{19})
					15.87 (C_{20})

* detected from ^{1}H,^{13}C HMBC, ^{1}H,^{13}C HSQC

Table S6. ^{15}N NMR (DMSO-d_6)

		**HL^1** (*E*-)	**HL^2** (*E*-)	**HL^3** (*E*-)	**H*L*$^{2c'}$**·CH_3COOH (*E*-)
N_1	*N*$_{py}$	315.07	310.61	321.53	309.86
$N_{3'}$	*N*H$_2$	-	-	71.10	-
N_8	C=*N*	325.04	312.94	312.8	337.75
N_9	*N*H (closer to py)	174.22	168.53	174.57	n.a.
N_{11}	*N*H (closer to ph)	128.93	129.34	126.69	-

Assignment of *E*- and *Z*-isomers in $\mathbf{HL^1}$–$\mathbf{HL^3}$. The assignment of *E*- and *Z*-isomers was based on ^{1}H, ^{1}H NOESY cross-peaks between N*H*(9) and protons of R_1 in *E*-isomer, whereas the 'diagnostic' signature of *Z*-isomer is the downfield shifted proton N*H*(9) due to the hydrogen bond to pyridine nitrogen atom. The N*H* signals of the *Z*-isomer of $\mathbf{HL^1}$–$\mathbf{HL^3}$ are seen at 14.26, 14.40, 14.57 (H_9) and 10.17, 9.99, 9.95 (H_{11}) ppm, while those for the *E*-isomer at 11.86, 10.46, 11.47 (H_9), and 10.00, 9.94, 9.70 (H_{11}) ppm. The *E*/*Z* ratio is 23:1, 17:1 and 31:1 for $\mathbf{HL^1}$–$\mathbf{HL^3}$, respectively. The predominance of *E*-isomer in DMSO makes the assignment of other protons of minor *Z*-isomer difficult, since they are often overlapped with the proton resonances of the *E*-isomer. Two N*H* signals for *E*-isomers were assigned based on ^{1}H, ^{1}H NOESY spectra, where the correlations between the H_{11} signal and the C*H* protons (H_{13} and H_{17}) of phenolic moiety, as well as between the H_9 signal and the aldimine C*H* proton (H_7) in $\mathbf{HL^1}$, $\mathbf{HL^3}$ or the methyl protons at the ketimine group ($H_{7'}$) in $\mathbf{HL^2}$ were found.

Even more remarkable differences due to the presence of two different isomeric forms in DMSO were observed in the ^{13}C NMR spectrum of $\mathbf{HL^2}$. The carbon atom of the methyl group ($C_{7'}$) in *E*-isomer resonates at 12.31 ppm, whereas in *Z*-isomer at 21.73 ppm, as also reported for 2-acetylpyridine *N*,*N*-dimethyl(chalcogen)semicarbazones.[23] Of note is also that the signals of methyl protons in both isomers in the ^{1}H NMR spectrum show almost the same chemical shifts at 2.44 (*E*) and 2.43 (*Z*) ppm. The protons of the amine group ($H_{3'}$) in $\mathbf{HL^3}$ due to the hydrogen bond formation to nitrogen N8 in *E*-isomer resonate at 6.49 ppm, whereas in the case of *Z*-isomer the signal of $H_{3'}$ protons is slightly upfield shifted to 6.21 ppm.

Assignment of resonances in $\mathbf{H}\boldsymbol{L}^{\mathbf{1a'}}$ and $\mathbf{H}\boldsymbol{L}^{\mathbf{1a''}}$. The protons H_{13}/H_{17}, H_{19}/H_{20}, and carbon atoms C_{13}/C_{17}, C_{14}/C_{16} and C_{19}/C_{20} in $\mathbf{H}\boldsymbol{L}^{\mathbf{1a''}}$ are magnetically non-equivalent in contrast to these atoms in $\mathbf{HL^1}$–$\mathbf{HL^3}$ and $\mathbf{H}\boldsymbol{L}^{\mathbf{1a'}}$. This can be explained by the involvement of atom H_{13} and nitrogen atom N_9 in hydrogen bond formation, the latter being more electronegative than the S atom of 1,3,4-thiadiazole ring (see Scheme S5). It is also worth mentioning the different multiplicity of protons H_{13}/H_{17}, H_{19}/H_{20} of 1,4-benzoquinone imine moiety in $\mathbf{H}\boldsymbol{L}^{\mathbf{1a''}}$. The protons of C*H* ($H_{13}$+$H_{17}$) or C*H*$_3$ (H_{19}+H_{20}) groups in $\mathbf{HL^1}$–$\mathbf{HL^3}$ and $\mathbf{H}\boldsymbol{L}^{\mathbf{1a'}}$ appear as singlets with intensity ratio 2:6, correspondingly, whereas in $\mathbf{H}\boldsymbol{L}^{\mathbf{1a''}}$ as two doublets of quartet for H_{13} and H_{17} (dq, ^4J = 2.8 (or 2.7), 1.3 (or 1.5) Hz, coupled with C*H* and C*H$_3$* protons, respectively) and as two doublets for methyl protons H_{20} and H_{19} (d, ^4J = 1.5 Hz, coupled with the C*H* proton) with the intensity ratio 1(dq):1(dq):3(d):3(d). The resonance signal of hydrogen bond proton H_{13} is downfield shifted to 7.46 ppm vs 7.20 ppm for atom H_{17}. The assignment of methyl groups in $\mathbf{H}\boldsymbol{L}^{\mathbf{1a''}}$ was done

accounting for the correlation between the C*H* and the CH_3 protons (H_{13} – H_{19}, H_{17} – H_{20}) in the ^{1}H, ^{1}H NOESY spectrum.

19. Complexation of H$L^{1a'}$ and H$L^{1a''}$

Table S7. Summarized ESI-MS experiment of the complexation of 1,3,4-thiadiazoles **H***L*$^{1a'}$ and **H***L*$^{1a''}$

H*L*	Reaction conditions	Positive ESI-MS peaks detected			
**H***L*$^{1a'}$	MeOH, r.t. under argon<br>MeOH, r.t. under air<br>MeOH, 50°C under air, 3 h<br>DMF, r.t., under air, 10 min<br>DMF, ice bad, under air, 1 h	659.16<br>$[Cu(\mathbf{H}L^{1a'})_2]^+$<br>657.13<br>$[Cu(\mathbf{H}L^{1a'})(\mathbf{H}L^{1a''})]^+$<br>655.18<br>$[Cu(\mathbf{H}L^{1a''})_2]^+$	402.10<br>$[Cu(\mathbf{H}L^{1a'})+CH_3CN]^+$<br>400.10<br>$[Cu(\mathbf{H}L^{1a''})+CH_3CN]^+$		
	chromatography<br>(SiO_2, MeOH)	655.18<br>$[Cu(\mathbf{H}L^{1a''})_2]^+$	400.10<br>$[Cu(\mathbf{H}L^{1a''})+CH_3CN]^+$	537.15<br>$[Cu(\mathbf{H}L^{1a''})(\mathbf{H}L^{1d})]^+$	
**H***L*$^{1a''}$	MeOH, 50°C under air, 1 h			537.15<br>$[Cu(\mathbf{H}L^{1a''})(\mathbf{H}L^{1d})]^+$	419.08<br>$[Cu(\mathbf{H}L^{1d})_2]^+$
	MeOH, 50°C under air, 36 h				419.08<br>$[Cu(\mathbf{H}L^{1d})_2]^+$

20. Cell apoptosis assay

Table S8. Gated events (%) in Colo320 cells measured by flow cytometry with 3 h induction time

**Colo320 colon adenocarcinoma cells**		Gated events %		
A+: annexin positive<br>I+: propidium iodide positive	Concentration	**Early apoptosis %**<br>**Q3**	**Late apoptosis** and **necrosis %**<br>**Q2**	**Cell death %**<br>**Q1**
Control A-I+	-	0	0	0.074
Control A+I-	-	0.032	0.010	5.24
Control A+I+	-	3.70	5.34	3.44
Control A+I+	-	3.25	9.99	6.93
DMSO	0.8%	3.26	3.05	1.35
M627 (12*H*-benzophenothiazine) *positive control*	10 µM	12.4	31.5	4.59
M627 (12*H*-benzophenothiazine) *positive control*	20 µM	11.1	26.1	7.85
cisplatin (Teva) *positive control*	15 µM	1.68	2.24	1.64
cisplatin (Teva) *positive control*	30 µM	4.90	4.07	0.79
**HL**^{1}	4 µM	6.19	12.4	2.79
**HL**^{1}	8 µM	7.65	7.0	1.57
**Cu(L**^{2}**)Cl**	0.25 µM	6.28	5.12	1.13
**Cu(L**^{2}**)Cl**	0.5 µM	6.33	17.2	3.76
Control A+I+	-	4.73	7.18	2.89

Table S9. Percentage of cell cycle distributions of SW480 cells induced by the TSC **HL**^{1} and complexes **1** and **2´** at the concentration of 10 µM for 24 h compared to the negative control

Cell cycle phase	Negativ control	**HL**^{1}	**1**	**2´**
G0/G1	49.1 ± 2.1	49.2 ± 1.6	38.9 ± 1.7	31.8 ± 0.6
S	29.8 ± 3.0	37.1 ± 4.7	44.0 ± 4.5	46.5 ± 1.8
G2/M	21.4 ± 4.6	12.3 ± 4.4	21.0 ± 6.2	22.2 ± 1.9

21. Computational data

Geometry optimization of *s-trans*- and *s-cis*-conformers around the N2-C3 thioamide bond for *E*- and *Z*-isomers of HL[1]–HL[3]

Data 1: Cartesian coordinates and energies of the optimized geometry for the *s-trans*-conformer of (*E*)-**HL[1]** (DMSO solution).

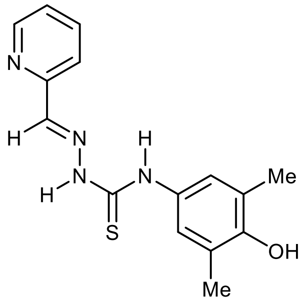

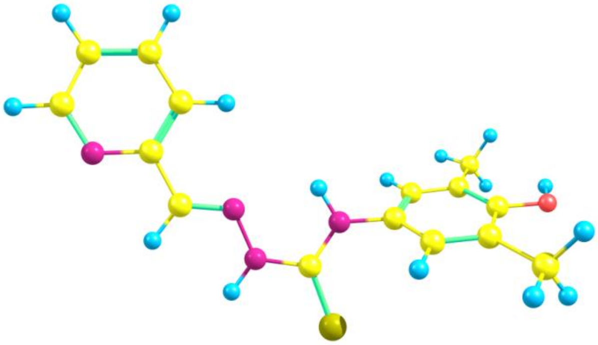

Electronic Energy =	-1273.92279515 a.u.
Zero-point correction =	0.290366 (Hartree/Particle)
Thermal correction to Energy =	0.310592
Thermal correction to Enthalpy =	0.311537
Thermal correction to Gibbs Free Energy =	0.238748
Sum of electronic and zero-point Energies =	-1273.632429
Sum of electronic and thermal Energies =	-1273.612203
Sum of electronic and thermal Enthalpies =	-1273.611259
Sum of electronic and thermal Free Energies =	-1273.684047

Standard orientation:

Center Number	Atomic Number	Atomic Type	Coordinates (Angstroms) X	Y	Z
1	6	0	6.354331	-1.876923	0.484469
2	6	0	5.022374	-2.281758	0.567958
3	6	0	4.020322	-1.345744	0.353563
4	6	0	4.385427	-0.024199	0.059896
5	7	0	5.669748	0.371471	-0.021612
6	6	0	6.622125	-0.542318	0.187761
7	7	0	2.139385	0.809972	-0.142103
8	7	0	1.305047	1.850332	-0.384060
9	6	0	-0.057903	1.695421	-0.379636
10	16	0	-1.026132	3.039151	-0.733290
11	7	0	-0.485091	0.453246	-0.096686
12	6	0	-1.840524	0.004615	-0.014167
13	6	0	-2.274403	-0.993306	-0.882296
14	6	0	-3.572125	-1.504034	-0.798827
15	6	0	-4.428186	-0.974129	0.177792
16	6	0	-4.010072	0.028557	1.067887
17	6	0	-2.703752	0.501626	0.961281
18	6	0	-4.957861	0.560914	2.111552
19	6	0	-4.044863	-2.589625	-1.734778
20	8	0	-5.720569	-1.413485	0.320114
21	6	0	3.401237	1.035836	-0.184541
22	1	0	1.663572	2.771749	-0.617638
23	1	0	0.247970	-0.240446	0.013234
24	1	0	-5.921282	-2.089256	-0.337672

25	1	0	7.166983	-2.574533	0.644307
26	1	0	4.771091	-3.310909	0.796334
27	1	0	2.974526	-1.618220	0.408562
28	1	0	7.647030	-0.191227	0.114744
29	1	0	-1.596654	-1.378703	-1.635988
30	1	0	-2.355303	1.265447	1.645510
31	1	0	-4.469784	1.318908	2.725736
32	1	0	-5.842853	1.009888	1.650723
33	1	0	-5.314964	-0.237684	2.768402
34	1	0	-4.905379	-2.268196	-2.332659
35	1	0	-4.338423	-3.496052	-1.192911
36	1	0	-3.253560	-2.869010	-2.430803
37	1	0	3.809319	2.024966	-0.404396

Data 2: Cartesian coordinates and energies of the optimized geometry for the *s*-*cis*-conformer of (*E*)-**HL**[1] (DMSO solution).

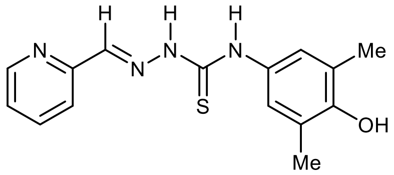

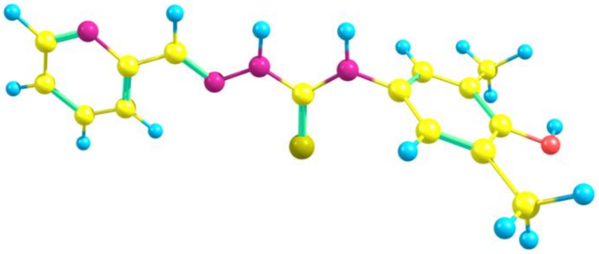

Electronic Energy =	-1273.91322461 a.u.
Zero-point correction =	0.289784 (Hartree/Particle)
Thermal correction to Energy =	0.310316
Thermal correction to Enthalpy =	0.311261
Thermal correction to Gibbs Free Energy =	0.236149
Sum of electronic and zero-point Energies =	-1273.623441
Sum of electronic and thermal Energies =	-1273.602908
Sum of electronic and thermal Enthalpies =	-1273.601964
Sum of electronic and thermal Free Energies =	-1273.677075

Standard orientation:

Center Number	Atomic Number	Atomic Type	Coordinates (Angstroms) X	Y	Z
1	6	0	-7.403343	0.211676	0.505771
2	6	0	-6.366146	0.604539	1.351675
3	6	0	-5.054316	0.417087	0.939585
4	6	0	-4.819235	-0.161892	-0.315835
5	7	0	-5.816661	-0.543016	-1.135531
6	6	0	-7.074369	-0.355341	-0.723146
7	7	0	-2.423788	-0.082110	-0.166164
8	7	0	-1.226563	-0.343876	-0.737898
9	6	0	-0.029096	-0.062044	-0.124829
10	16	0	0.096594	0.692569	1.377698
11	7	0	1.035870	-0.460041	-0.862417
12	6	0	2.415456	-0.269656	-0.516982
13	6	0	3.152631	-1.337414	-0.015552
14	6	0	4.510979	-1.195072	0.278200
15	6	0	5.105736	0.056576	0.057211
16	6	0	4.381876	1.146917	-0.452805
17	6	0	3.031385	0.960094	-0.741094
18	6	0	5.063191	2.471549	-0.680698
19	6	0	5.319125	-2.349132	0.819770
20	8	0	6.432264	0.280551	0.324345
21	6	0	-3.467881	-0.399985	-0.838100

22	1	0	-1.218199	-0.777935	-1.659166
23	1	0	0.861386	-0.975774	-1.717040
24	1	0	6.845794	-0.518165	0.671661
25	1	0	-8.440959	0.339622	0.788033
26	1	0	-6.578985	1.049495	2.316690
27	1	0	-4.219398	0.706657	1.564075
28	1	0	-7.855202	-0.673270	-1.407522
29	1	0	2.665447	-2.291447	0.151956
30	1	0	2.449649	1.782520	-1.141175
31	1	0	4.363003	3.200611	-1.090858
32	1	0	5.470058	2.873227	0.252162
33	1	0	5.903220	2.372198	-1.374407
34	1	0	6.148272	-2.615144	0.154072
35	1	0	4.695520	-3.236345	0.931959
36	1	0	5.744724	-2.123661	1.804259
37	1	0	-3.415910	-0.862209	-1.827589

Data 3: Cartesian coordinates and energies of the optimized geometry for the *s*-*trans*-conformer of (*Z*)-**HL**[1] (DMSO solution).

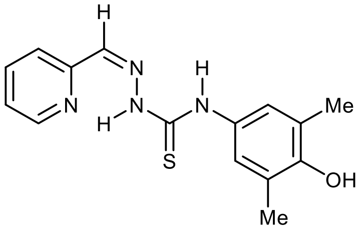

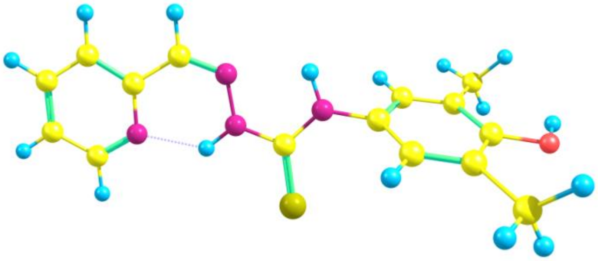

Electronic Energy =	-1273.92510682 a.u.
Zero-point correction =	0.290951 (Hartree/Particle)
Thermal correction to Energy =	0.310883
Thermal correction to Enthalpy =	0.311827
Thermal correction to Gibbs Free Energy =	0.239855
Sum of electronic and zero-point Energies =	-1273.634156
Sum of electronic and thermal Energies =	-1273.614224
Sum of electronic and thermal Enthalpies=	-1273.613280
Sum of electronic and thermal Free Energies=	-1273.685251

Standard orientation:

Center Number	Atomic Number	Atomic Type	Coordinates (Angstroms) X	Y	Z
1	6	0	6.660772	0.595628	-0.414896
2	6	0	6.762452	-0.562047	0.353143
3	6	0	5.598466	-1.190785	0.776857
4	6	0	4.358412	-0.641034	0.418763
5	7	0	4.263846	0.482332	-0.325951
6	6	0	5.390323	1.076347	-0.726770
7	7	0	1.895795	-0.993456	0.668379
8	7	0	1.591263	0.111097	-0.054136
9	6	0	0.283260	0.449436	-0.286942
10	16	0	-0.049410	1.826932	-1.217498
11	7	0	-0.625387	-0.379623	0.253884
12	6	0	-2.049138	-0.266572	0.179211
13	6	0	-2.773426	-1.248662	-0.490358
14	6	0	-4.169411	-1.208044	-0.534297
15	6	0	-4.819071	-0.145370	0.110853
16	6	0	-4.109234	0.855425	0.794323

17	6	0	-2.718495	0.772960	0.823501
18	6	0	-4.847024	1.975877	1.480857
19	6	0	-4.958609	-2.269706	-1.261023
20	8	0	-6.186678	-0.028940	0.111421
21	6	0	3.131638	-1.309698	0.867175
22	1	0	2.352039	0.677647	-0.440824
23	1	0	-0.235349	-1.191362	0.724017
24	1	0	-6.587865	-0.762777	-0.368273
25	1	0	7.541137	1.118272	-0.766791
26	1	0	7.731913	-0.967629	0.616871
27	1	0	5.638217	-2.092786	1.375082
28	1	0	5.271631	1.975407	-1.322833
29	1	0	-2.246628	-2.055771	-0.987357
30	1	0	-2.149701	1.526348	1.354954
31	1	0	-4.148085	2.655793	1.969982
32	1	0	-5.444695	2.551905	0.768033
33	1	0	-5.539757	1.591973	2.235550
34	1	0	-5.651163	-2.793675	-0.592450
35	1	0	-4.292457	-3.019000	-1.689368
36	1	0	-5.547850	-1.848685	-2.083688
37	1	0	3.268490	-2.210010	1.458089

Data 4: Cartesian coordinates and energies of the optimized geometry for the *s*-*cis*-conformer of (*Z*)-**HL**[1] (DMSO solution).

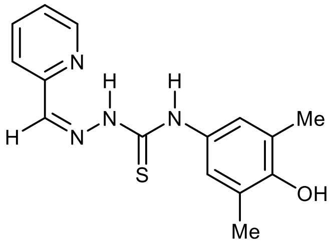

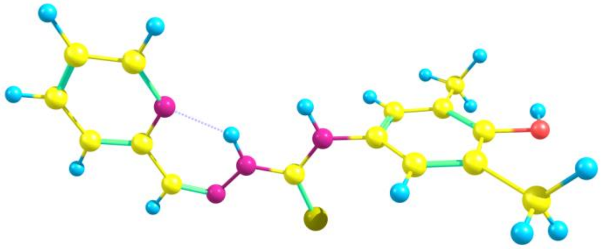

Electronic Energy =	-1273.91615582 a.u.
Zero-point correction =	0.290434 (Hartree/Particle)
Thermal correction to Energy =	0.309645
Thermal correction to Enthalpy =	0.310589
Thermal correction to Gibbs Free Energy =	0.240966
Sum of electronic and zero-point Energies =	-1273.625722
Sum of electronic and thermal Energies =	-1273.606511
Sum of electronic and thermal Enthalpies =	-1273.605567
Sum of electronic and thermal Free Energies =	-1273.675190

Standard orientation:

Center Number	Atomic Number	Atomic Type	Coordinates (Angstroms) X	Y	Z
1	6	0	-5.736314	2.161206	0.447164
2	6	0	-6.504162	1.021234	0.217872
3	6	0	-5.860405	-0.184280	-0.029962
4	6	0	-4.457984	-0.218277	-0.042171
5	7	0	-3.717798	0.890248	0.180967
6	6	0	-4.348654	2.043480	0.418124
7	7	0	-2.518563	-1.771466	-0.365938
8	7	0	-1.623851	-0.778751	-0.166512
9	6	0	-0.270013	-1.001679	-0.215046
10	16	0	0.437287	-2.510801	-0.484485
11	7	0	0.439575	0.137540	-0.023579
12	6	0	1.869189	0.245245	0.020726

13	6	0	2.560063	0.712396	-1.092687
14	6	0	3.946795	0.877700	-1.058823
15	6	0	4.620597	0.555938	0.129326
16	6	0	3.943393	0.087795	1.267214
17	6	0	2.559119	-0.056017	1.193500
18	6	0	4.706310	-0.240088	2.524641
19	6	0	4.703646	1.385312	-2.261919
20	8	0	5.981265	0.687761	0.243828
21	6	0	-3.778112	-1.493414	-0.304819
22	1	0	-2.012291	0.156827	0.024074
23	1	0	-0.076410	1.004250	0.072251
24	1	0	6.360703	1.001191	-0.585337
25	1	0	-6.196481	3.121078	0.644281
26	1	0	-7.586358	1.071923	0.232238
27	1	0	-6.424776	-1.090593	-0.212228
28	1	0	-3.716774	2.908359	0.592031
29	1	0	2.014421	0.947834	-1.999556
30	1	0	2.010357	-0.410859	2.058333
31	1	0	4.029165	-0.580855	3.309130
32	1	0	5.447184	-1.024898	2.345176
33	1	0	5.253520	0.631338	2.896182
34	1	0	5.218768	2.329520	-2.051632
35	1	0	4.025140	1.563969	-3.096381
36	1	0	5.457182	0.666488	-2.603460
37	1	0	-4.426398	-2.347143	-0.477374

Data 5: Cartesian coordinates and energies of the optimized geometry for the *s-trans*-conformer of (*E*)-**HL**^{2} (DMSO solution).

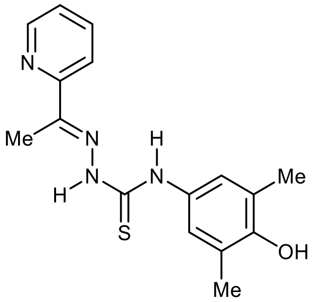

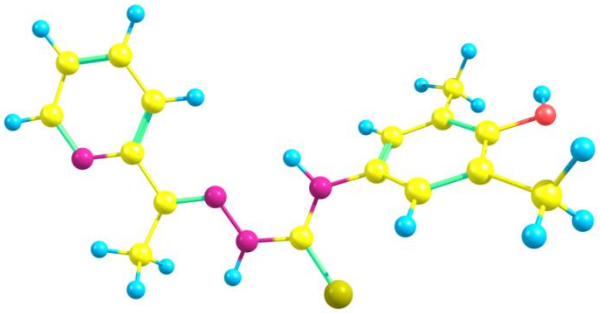

Electronic Energy =	-1313.25113329 a.u.
Zero-point correction =	0.317993 (Hartree/Particle)
Thermal correction to Energy =	0.339858
Thermal correction to Enthalpy =	0.340802
Thermal correction to Gibbs Free Energy =	0.263747
Sum of electronic and zero-point Energies =	-1312.933140
Sum of electronic and thermal Energies =	-1312.911275
Sum of electronic and thermal Enthalpies =	-1312.910331
Sum of electronic and thermal Free Energies =	-1312.987386

Standard orientation:

Center Number	Atomic Number	Atomic Type	Coordinates (Angstroms) X	Y	Z
1	6	0	5.773179	-2.460848	0.491110
2	6	0	4.391878	-2.641751	0.560430
3	6	0	3.558347	-1.550015	0.367322
4	6	0	4.129701	-0.293556	0.107115
5	7	0	5.460191	-0.120262	0.042662
6	6	0	6.252444	-1.181127	0.230335
7	7	0	2.009304	0.736192	-0.093054

8	7	0	1.200705	1.809045	-0.290196
9	6	0	-0.165367	1.677993	-0.300354
10	16	0	-1.110003	3.053271	-0.597170
11	7	0	-0.614336	0.431387	-0.078133
12	6	0	-1.973497	-0.005751	-0.018106
13	6	0	-2.402367	-0.991196	-0.903259
14	6	0	-3.704011	-1.494997	-0.842878
15	6	0	-4.569996	-0.970978	0.128025
16	6	0	-4.157444	0.018681	1.035032
17	6	0	-2.846981	0.485084	0.951578
18	6	0	-5.115529	0.544299	2.072770
19	6	0	-4.170340	-2.567066	-1.797452
20	8	0	-5.867002	-1.404012	0.248118
21	6	0	3.287957	0.915536	-0.112905
22	1	0	1.565190	2.735294	-0.481601
23	1	0	0.111663	-0.274324	-0.002152
24	1	0	-6.062744	-2.071400	-0.419638
25	1	0	6.460288	-3.285264	0.635556
26	1	0	3.972045	-3.620260	0.762848
27	1	0	2.483792	-1.655390	0.415984
28	1	0	7.320814	-0.996088	0.169030
29	1	0	-1.717115	-1.372520	-1.652282
30	1	0	-2.503542	1.238259	1.649752
31	1	0	-4.630660	1.291118	2.702993
32	1	0	-5.991335	1.005122	1.606092
33	1	0	-5.486680	-0.260073	2.714627
34	1	0	-5.019240	-2.232930	-2.404900
35	1	0	-4.478792	-3.476902	-1.269757
36	1	0	-3.370556	-2.845116	-2.484274
37	6	0	3.937446	2.252107	-0.341811
38	1	0	3.660372	2.657495	-1.321025
39	1	0	3.620693	2.972374	0.420192
40	1	0	5.018304	2.157341	-0.302328

Data 6: Cartesian coordinates and energies of the optimized geometry for the *s-cis*-conformer of (*E*)-**HL**^{2} (DMSO solution).

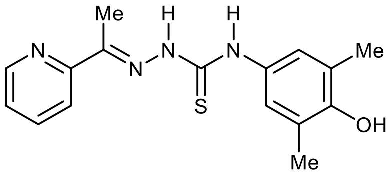

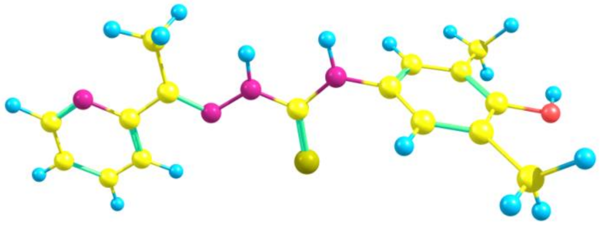

Electronic Energy =	-1313.24090817 a.u.
Zero-point correction =	0.317413 (Hartree/Particle)
Thermal correction to Energy =	0.339522
Thermal correction to Enthalpy =	0.340466
Thermal correction to Gibbs Free Energy =	0.262335
Sum of electronic and zero-point Energies =	-1312.923495
Sum of electronic and thermal Energies =	-1312.901386
Sum of electronic and thermal Enthalpies =	-1312.900442
Sum of electronic and thermal Free Energies =	-1312.978573

Standard orientation:

Center Number	Atomic Number	Atomic Type	Coordinates (Angstroms) X	Y	Z
1	6	0	-7.083824	-0.889030	-0.618321
2	6	0	-5.925049	-1.503056	-1.094885
3	6	0	-4.690433	-0.976928	-0.745542

4	6	0	-4.642717	0.159747	0.079019
5	7	0	-5.758522	0.751021	0.536071
6	6	0	-6.942941	0.232723	0.191662
7	7	0	-2.283891	0.156881	0.074584
8	7	0	-1.080333	0.671516	0.419861
9	6	0	0.107000	0.089141	0.042965
10	16	0	0.210044	-1.364093	-0.806180
11	7	0	1.183941	0.817227	0.430766
12	6	0	2.559899	0.459044	0.248795
13	6	0	3.330991	1.154239	-0.677241
14	6	0	4.689758	0.870334	-0.837653
15	6	0	5.249789	-0.141465	-0.043456
16	6	0	4.491057	-0.853991	0.900078
17	6	0	3.142711	-0.531474	1.037470
18	6	0	5.135525	-1.928428	1.737327
19	6	0	5.531974	1.625048	-1.837466
20	8	0	6.573928	-0.486528	-0.141857
21	6	0	-3.343661	0.765752	0.488528
22	1	0	-1.045356	1.524053	0.970881
23	1	0	1.019013	1.730437	0.837265
24	1	0	7.013183	0.047905	-0.813364
25	1	0	-8.068037	-1.266414	-0.866724
26	1	0	-5.985567	-2.379038	-1.730412
27	1	0	-3.770477	-1.424047	-1.095636
28	1	0	-7.818894	0.741329	0.583586
29	1	0	2.870790	1.925454	-1.284869
30	1	0	2.537144	-1.059762	1.764522
31	1	0	4.411970	-2.367906	2.425317
32	1	0	5.542944	-2.727455	1.110654
33	1	0	5.969657	-1.529854	2.322195
34	1	0	6.353588	2.165697	-1.353793
35	1	0	4.928836	2.360463	-2.370471
36	1	0	5.971195	0.958582	-2.588546
37	6	0	-3.331787	2.010449	1.335028
38	1	0	-4.347887	2.323729	1.555928
39	1	0	-2.810436	1.834478	2.282329
40	1	0	-2.821903	2.830933	0.818043

Data 7: Cartesian coordinates and energies of the optimized geometry for the *s*-*trans*-conformer of (*Z*)-**HL**^{2} (DMSO solution).

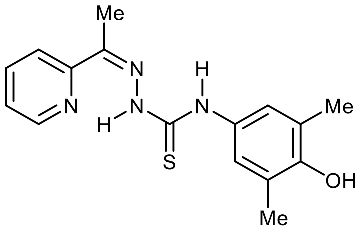

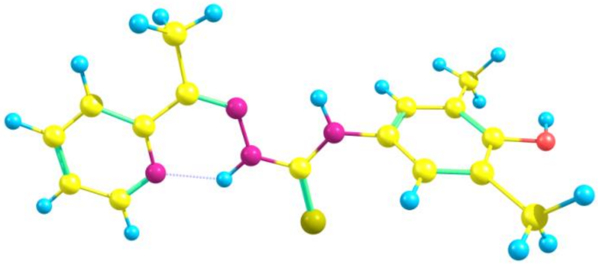

Electronic Energy = -1313.25257029 a.u.
Zero-point correction = 0.318281 (Hartree/Particle)
Thermal correction to Energy = 0.339815
Thermal correction to Enthalpy = 0.340760
Thermal correction to Gibbs Free Energy = 0.265192
Sum of electronic and zero-point Energies = -1312.934290
Sum of electronic and thermal Energies = -1312.912755
Sum of electronic and thermal Enthalpies = -1312.911811
Sum of electronic and thermal Free Energies = -1312.987378

Standard orientation:

Center Number	Atomic Number	Atomic Type	Coordinates (Angstroms) X	Y	Z
1	6	0	-6.410224	-1.080010	-0.432918
2	6	0	-6.607361	0.202899	0.068490
3	6	0	-5.502355	0.987009	0.379325
4	6	0	-4.212560	0.468127	0.181190
5	7	0	-4.033946	-0.779569	-0.306846
6	6	0	-5.101108	-1.523623	-0.602092
7	7	0	-1.773092	0.924599	0.373502
8	7	0	-1.434183	-0.301527	-0.098492
9	6	0	-0.117439	-0.641758	-0.240484
10	16	0	0.271161	-2.173785	-0.862446
11	7	0	0.762942	0.305994	0.126926
12	6	0	2.189618	0.220319	0.091296
13	6	0	2.898819	1.053776	-0.768989
14	6	0	4.295905	1.043256	-0.791982
15	6	0	4.963262	0.161991	0.071443
16	6	0	4.269392	-0.684953	0.951040
17	6	0	2.876708	-0.635367	0.951429
18	6	0	5.025555	-1.609660	1.869881
19	6	0	5.067496	1.947790	-1.721892
20	8	0	6.333425	0.084516	0.106672
21	6	0	-3.011831	1.282255	0.503719
22	1	0	-2.180752	-0.951322	-0.364786
23	1	0	0.342082	1.185009	0.414356
24	1	0	6.722323	0.695671	-0.529560
25	1	0	-7.242775	-1.723393	-0.687660
26	1	0	-7.608059	0.591067	0.216726
27	1	0	-5.643799	1.984709	0.769254
28	1	0	-4.898844	-2.516690	-0.990431
29	1	0	2.358921	1.719658	-1.433122
30	1	0	2.319688	-1.271175	1.629049
31	1	0	4.337318	-2.183091	2.492449
32	1	0	5.643532	-2.312914	1.303556
33	1	0	5.701515	-1.053112	2.525748
34	1	0	5.723684	2.633349	-1.173533
35	1	0	4.387590	2.556748	-2.318225
36	1	0	5.692435	1.379300	-2.420095
37	6	0	-3.212490	2.677700	1.041797
38	1	0	-2.241742	3.135810	1.223259
39	1	0	-3.765886	3.302648	0.334752
40	1	0	-3.773563	2.667179	1.980875

Data 8: Cartesian coordinates and energies of the optimized geometry for the *s*-*cis*-conformer of (*Z*)-**HL**^{2} (DMSO solution).

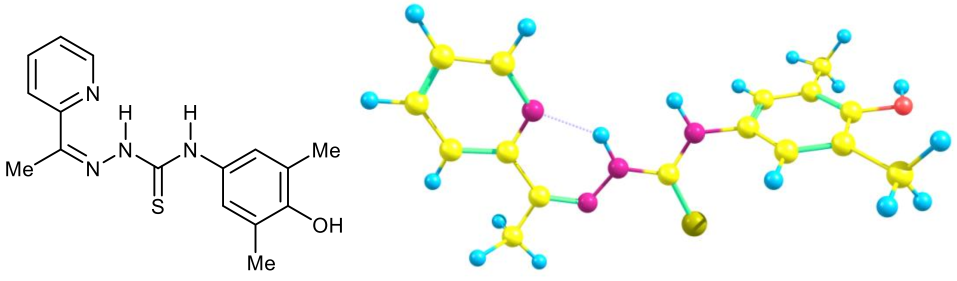

Electronic Energy = -1313.24330419 a.u.
Zero-point correction = 0.317896 (Hartree/Particle)
Thermal correction to Energy = 0.339566
Thermal correction to Enthalpy = 0.340511
Thermal correction to Gibbs Free Energy = 0.264186

Sum of electronic and zero-point Energies = -1312.925408
Sum of electronic and thermal Energies = -1312.903738
Sum of electronic and thermal Enthalpies = -1312.902794
Sum of electronic and thermal Free Energies = -1312.979118

Standard orientation:

Center Number	Atomic Number	Atomic Type	Coordinates (Angstroms) X	Y	Z
1	6	0	-5.265614	2.595963	0.614420
2	6	0	-6.139591	1.540176	0.372104
3	6	0	-5.622708	0.285023	0.073205
4	6	0	-4.230506	0.107217	0.022150
5	7	0	-3.393045	1.141949	0.259285
6	6	0	-3.898641	2.343475	0.545251
7	7	0	-2.385738	-1.527886	-0.366184
8	7	0	-1.438056	-0.586607	-0.149963
9	6	0	-0.102499	-0.883642	-0.208685
10	16	0	0.522143	-2.431693	-0.473768
11	7	0	0.670416	0.220031	-0.031880
12	6	0	2.100730	0.266945	0.011173
13	6	0	2.793783	0.896297	-1.018529
14	6	0	4.185503	1.017620	-0.980101
15	6	0	4.863978	0.478230	0.122577
16	6	0	4.185697	-0.158380	1.175215
17	6	0	2.797124	-0.246595	1.104454
18	6	0	4.953205	-0.716403	2.345965
19	6	0	4.940637	1.701698	-2.093734
20	8	0	6.230098	0.548924	0.234549
21	6	0	-3.643379	-1.222519	-0.292327
22	1	0	-1.781355	0.363106	0.064912
23	1	0	0.200160	1.116575	-0.000612
24	1	0	6.607912	0.999767	-0.529362
25	1	0	-5.627116	3.588640	0.850718
26	1	0	-7.211920	1.689744	0.414739
27	1	0	-6.292609	-0.541149	-0.116540
28	1	0	-3.178423	3.134827	0.726201
29	1	0	2.246167	1.296569	-1.864667
30	1	0	2.250080	-0.723377	1.909063
31	1	0	4.274769	-1.159496	3.076376
32	1	0	5.661903	-1.485689	2.024738
33	1	0	5.537487	0.061813	2.845721
34	1	0	5.508823	2.566226	-1.731720
35	1	0	4.253452	2.062833	-2.859289
36	1	0	5.647741	1.023466	-2.584788
37	6	0	-4.584579	-2.372256	-0.561185
38	1	0	-5.232481	-2.168287	-1.418959
39	1	0	-4.002787	-3.267895	-0.772074
40	1	0	-5.230749	-2.571493	0.298905

Data 9: Cartesian coordinates and energies of the optimized geometry for the *s*-*trans*-conformer of (*E*)-**HL**^{3} (DMSO solution).

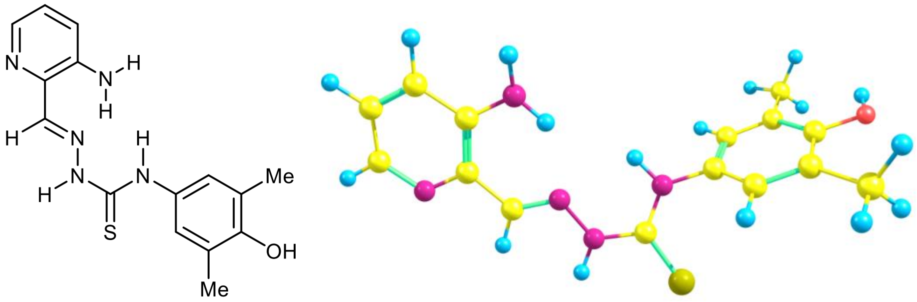

Electronic Energy =	-1329.3054550 a.u.
Zero-point correction =	0.306858 (Hartree/Particle)
Thermal correction to Energy =	0.328532
Thermal correction to Enthalpy =	0.329476
Thermal correction to Gibbs Free Energy =	0.253551
Sum of electronic and zero-point Energies =	-1328.998597
Sum of electronic and thermal Energies =	-1328.976923
Sum of electronic and thermal Enthalpies =	-1328.975979
Sum of electronic and thermal Free Energies =	-1329.051904

Standard orientation:

Center Number	Atomic Number	Atomic Type	Coordinates (Angstroms) X	Y	Z
1	6	0	6.502298	-1.495965	0.291889
2	6	0	5.263337	-2.009369	0.618894
3	6	0	4.108585	-1.219080	0.454614
4	6	0	4.295128	0.101109	-0.044809
5	7	0	5.519877	0.584917	-0.352840
6	6	0	6.587870	-0.184806	-0.196071
7	7	0	1.969237	0.815349	-0.088513
8	7	0	1.116415	1.860853	-0.299114
9	6	0	-0.241329	1.713164	-0.305812
10	16	0	-1.206783	3.085740	-0.558735
11	7	0	-0.684852	0.456051	-0.120611
12	6	0	-2.046045	0.023295	-0.051515
13	6	0	-2.514720	-0.885563	-0.996000
14	6	0	-3.819528	-1.380891	-0.931153
15	6	0	-4.646298	-0.928175	0.107532
16	6	0	-4.193153	-0.015211	1.073925
17	6	0	-2.881048	0.444669	0.982313
18	6	0	-5.110807	0.437394	2.180299
19	6	0	-4.330263	-2.367070	-1.953188
20	8	0	-5.943184	-1.358144	0.238364
21	6	0	3.224963	1.063408	-0.253867
22	1	0	1.476323	2.785633	-0.514897
23	1	0	0.029670	-0.262045	-0.112447
24	1	0	-6.164364	-1.982536	-0.462217
25	1	0	7.397980	-2.093235	0.412733
26	1	0	5.163994	-3.019334	1.001713
27	1	0	7.547654	0.248053	-0.459221
28	1	0	-1.859425	-1.212276	-1.795912
29	1	0	-2.505532	1.138561	1.724363
30	1	0	-4.595012	1.123877	2.853131
31	1	0	-5.992413	0.947024	1.779932
32	1	0	-5.475829	-0.411161	2.766252
33	1	0	-5.179800	-1.966870	-2.518132

34	1	0	-4.654219	-3.305684	-1.489276
35	1	0	-3.549666	-2.613922	-2.673213
36	1	0	3.567218	2.051754	-0.570221
37	7	0	2.869659	-1.728843	0.733710
38	1	0	2.102874	-1.076942	0.812224
39	1	0	2.818262	-2.557320	1.307301

Data 10: Cartesian coordinates and energies of the optimized geometry for the *s-cis*-conformer of (*E*)-**HL**[3] (DMSO solution).

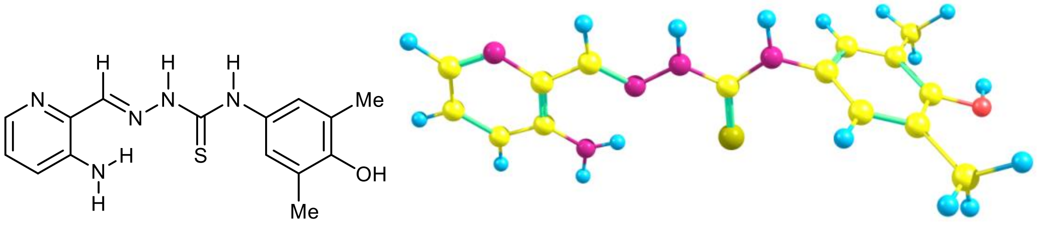

Electronic Energy =	-1329.29878937 a.u.
Zero-point correction =	0.306062 (Hartree/Particle)
Thermal correction to Energy =	0.328048
Thermal correction to Enthalpy =	0.328992
Thermal correction to Gibbs Free Energy =	0.251925
Sum of electronic and zero-point Energies =	-1328.992727
Sum of electronic and thermal Energies =	-1328.970742
Sum of electronic and thermal Enthalpies =	-1328.969798
Sum of electronic and thermal Free Energies =	-1329.046864

Standard orientation:

Center Number	Atomic Number	Atomic Type	Coordinates (Angstroms) X	Y	Z
1	6	0	-7.234294	0.201152	0.193219
2	6	0	-6.252195	0.843318	0.920859
3	6	0	-4.891992	0.573235	0.662872
4	6	0	-4.610760	-0.369827	-0.371339
5	7	0	-5.589070	-0.987337	-1.068069
6	6	0	-6.859578	-0.715986	-0.797775
7	7	0	-2.211726	-0.246000	-0.228100
8	7	0	-1.008650	-0.660227	-0.697430
9	6	0	0.171773	-0.193375	-0.183090
10	16	0	0.247867	0.966183	1.043400
11	7	0	1.260595	-0.760806	-0.758449
12	6	0	2.628398	-0.447097	-0.460335
13	6	0	3.375915	-1.313056	0.331219
14	6	0	4.724431	-1.060297	0.594629
15	6	0	5.299103	0.094289	0.041666
16	6	0	4.564957	0.979957	-0.764296
17	6	0	3.224984	0.686299	-1.009567
18	6	0	5.224754	2.204290	-1.344337
19	6	0	5.542503	-1.996428	1.450519
20	8	0	6.614874	0.417577	0.255895
21	6	0	-3.264749	-0.743629	-0.777023
22	1	0	-0.992527	-1.338330	-1.456688
23	1	0	1.113089	-1.507239	-1.427348
24	1	0	7.036364	-0.240299	0.820859
25	1	0	-8.282073	0.400972	0.383471
26	1	0	-6.513498	1.556034	1.695713
27	1	0	-7.608949	-1.237684	-1.384424
28	1	0	2.904200	-2.194190	0.751608
29	1	0	2.636316	1.349464	-1.632892

30	1	0	4.517773	2.773777	-1.949239
31	1	0	5.607932	2.858922	-0.555887
32	1	0	6.078260	1.935286	-1.973651
33	1	0	6.391260	-2.417226	0.899362
34	1	0	4.933864	-2.832002	1.796905
35	1	0	5.941710	-1.494191	2.339122
36	1	0	-3.209372	-1.475525	-1.587593
37	7	0	-3.909670	1.173717	1.391089
38	1	0	-4.148178	1.926610	2.016520
39	1	0	-2.940895	1.043583	1.125990

Data 11: Cartesian coordinates and energies of the optimized geometry for the *s-trans*-conformer of (*Z*)-**HL**[3] (DMSO solution).

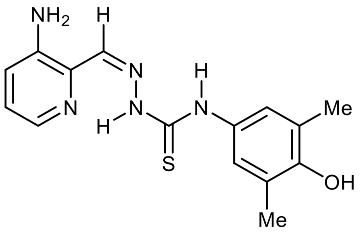

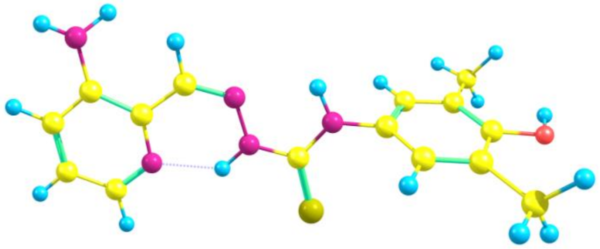

Electronic Energy =	-1329.30412103 a.u.
Zero-point correction =	0.307308 (Hartree/Particle)
Thermal correction to Energy =	0.328812
Thermal correction to Enthalpy =	0.329757
Thermal correction to Gibbs Free Energy =	0.253594
Sum of electronic and zero-point Energies =	-1328.996813
Sum of electronic and thermal Energies =	-1328.975309
Sum of electronic and thermal Enthalpies =	-1328.974364
Sum of electronic and thermal Free Energies =	-1329.050527

Standard orientation:

Center Number	Atomic Number	Atomic Type	Coordinates (Angstroms) X	Y	Z
1	6	0	-6.285087	-1.136183	-0.487999
2	6	0	-6.502376	0.129006	0.026292
3	6	0	-5.413996	0.944253	0.381614
4	6	0	-4.110285	0.396555	0.212013
5	7	0	-3.926239	-0.837274	-0.310041
6	6	0	-4.971339	-1.583044	-0.651194
7	7	0	-1.672415	0.875512	0.495502
8	7	0	-1.299930	-0.283015	-0.103917
9	6	0	0.024614	-0.583252	-0.267482
10	16	0	0.448734	-2.042894	-1.022707
11	7	0	0.884452	0.342895	0.189998
12	6	0	2.313703	0.276954	0.146698
13	6	0	3.010605	1.114605	-0.718878
14	6	0	4.407535	1.115318	-0.749025
15	6	0	5.086363	0.242733	0.114685
16	6	0	4.404479	-0.608514	0.999556
17	6	0	3.011312	-0.571776	1.004739
18	6	0	5.173185	-1.524275	1.916860
19	6	0	5.167335	2.023237	-1.685263
20	8	0	6.456964	0.178023	0.144315
21	6	0	-2.929835	1.157386	0.618964
22	1	0	-2.030191	-0.932439	-0.413163
23	1	0	0.451295	1.179485	0.569972

24	1	0	6.837894	0.792223	-0.493832
25	1	0	-7.117100	-1.769348	-0.770362
26	1	0	-7.511388	0.506994	0.150456
27	1	0	-4.758948	-2.563406	-1.063556
28	1	0	2.461208	1.774170	-1.381397
29	1	0	2.461737	-1.212571	1.684237
30	1	0	4.492941	-2.104738	2.541680
31	1	0	5.797461	-2.220935	1.349383
32	1	0	5.844575	-0.959738	2.570622
33	1	0	5.819545	2.716695	-1.142129
34	1	0	4.479581	2.623843	-2.281050
35	1	0	5.794437	1.457615	-2.383829
36	1	0	-3.093860	2.101514	1.125705
37	7	0	-5.638108	2.194880	0.923734
38	1	0	-6.576638	2.554550	0.822747
39	1	0	-4.939593	2.909205	0.786891

Data 12: Cartesian coordinates and energies of the optimized geometry for the *s*-*cis*-conformer of (*Z*)-**HL**^{3} (DMSO solution).

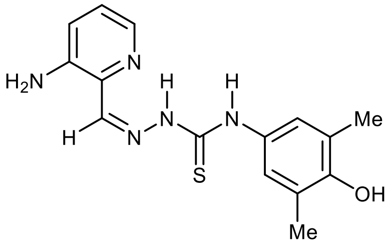

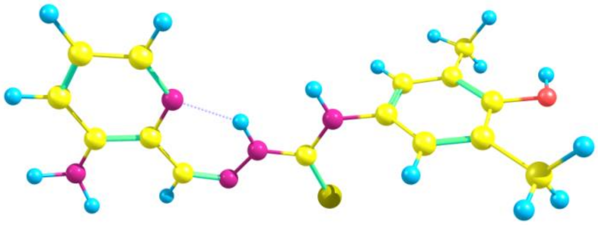

Electronic Energy =	-1329.2951134 a.u.
Zero-point correction =	0.306872 (Hartree/Particle)
Thermal correction to Energy =	0.327593
Thermal correction to Enthalpy =	0.328537
Thermal correction to Gibbs Free Energy =	0.255784
Sum of electronic and zero-point Energies =	-1328.988241
Sum of electronic and thermal Energies =	-1328.967520
Sum of electronic and thermal Enthalpies =	-1328.966576
Sum of electronic and thermal Free Energies =	-1329.039330

Standard orientation:

Center Number	Atomic Number	Atomic Type	Coordinates (Angstroms) X	Y	Z
1	6	0	-5.276443	2.400869	0.520168
2	6	0	-6.107099	1.309660	0.337216
3	6	0	-5.562172	0.042766	0.068227
4	6	0	-4.144287	-0.047564	-0.028787
5	7	0	-3.356347	1.033314	0.169871
6	6	0	-3.894585	2.219395	0.436480
7	7	0	-2.246844	-1.629921	-0.450307
8	7	0	-1.323209	-0.675114	-0.194089
9	6	0	0.021240	-0.935349	-0.247281
10	16	0	0.685412	-2.460393	-0.543621
11	7	0	0.765437	0.179038	-0.033545
12	6	0	2.196546	0.245084	0.020273
13	6	0	2.901875	0.763753	-1.060982
14	6	0	4.292373	0.892204	-1.016227
15	6	0	4.955599	0.477721	0.148634
16	6	0	4.264294	-0.044662	1.254106

17	6	0	2.876929	-0.146996	1.171799
18	6	0	5.016334	-0.471567	2.488229
19	6	0	5.063378	1.456339	-2.184742
20	8	0	6.319302	0.566043	0.271492
21	6	0	-3.500757	-1.319026	-0.364348
22	1	0	-1.692286	0.265936	0.015381
23	1	0	0.275280	1.061351	0.052928
24	1	0	6.707331	0.929742	-0.532767
25	1	0	-5.687542	3.379720	0.733848
26	1	0	-7.183915	1.419901	0.407567
27	1	0	-3.208460	3.045916	0.585144
28	1	0	2.364437	1.069585	-1.951693
29	1	0	2.318060	-0.540568	2.012982
30	1	0	4.328924	-0.840809	3.250600
31	1	0	5.734497	-1.264819	2.259943
32	1	0	5.588043	0.359001	2.912660
33	1	0	5.610454	2.366159	-1.912124
34	1	0	4.389192	1.715525	-3.001414
35	1	0	5.791167	0.737989	-2.579116
36	1	0	-4.147854	-2.154760	-0.605519
37	7	0	-6.398478	-1.035570	-0.147044
38	1	0	-7.358110	-0.910775	0.142395
39	1	0	-6.053494	-1.959148	0.064398

Pathways of Z/E-isomerization of 2-formylpyridine thiosemicarbazone

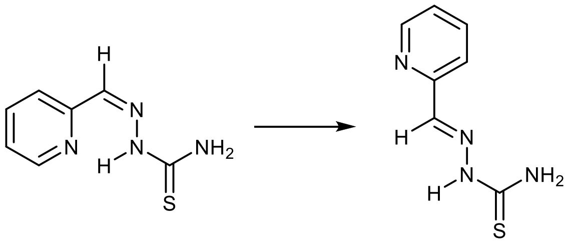

Z-isomer *E*-isomer

Pathway 1: *inversion at the imine nitrogen*

Data 13: Cartesian coordinates and energies of the optimized geometry for the *s-trans*-conformer of (*E*)-2-formylpyridine thiosemicarbazone (the gas phase).

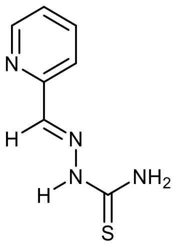

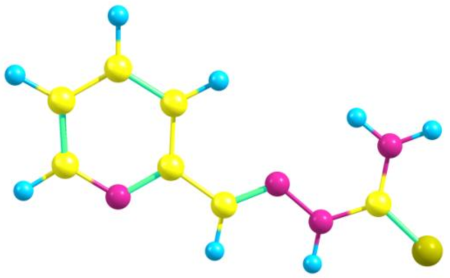

Electronic Energy =	-888.903948096 a.u.
Zero-point correction =	0.150920 (Hartree/Particle)
Thermal correction to Energy =	0.162107
Thermal correction to Enthalpy =	0.163051
Thermal correction to Gibbs Free Energy =	0.112272
Sum of electronic and zero-point Energies =	-888.753028

Sum of electronic and thermal Energies = -888.741842
Sum of electronic and thermal Enthalpies = -888.740897
Sum of electronic and thermal Free Energies = -888.791676

Standard orientation:

Center Number	Atomic Number	Atomic Type	Coordinates (Angstroms) X	Y	Z
1	7	0	-2.734797	1.582155	0.000009
2	6	0	-2.957665	0.254332	0.000006
3	16	0	-4.470016	-0.460424	-0.000024
4	7	0	-1.826491	-0.527053	0.000034
5	7	0	-0.584168	0.006379	0.000021
6	6	0	0.412457	-0.802118	0.000014
7	6	0	1.801146	-0.333263	0.000004
8	7	0	2.720722	-1.313890	-0.000000
9	6	0	4.008538	-0.966137	-0.000009
10	6	0	4.448645	0.355673	-0.000013
11	6	0	3.491256	1.369770	-0.000009
12	6	0	2.147578	1.026098	-0.000001
13	1	0	-1.792388	1.942562	0.000018
14	1	0	-3.528899	2.198351	-0.000007
15	1	0	-1.975561	-1.532220	-0.000000
16	1	0	0.285972	-1.887965	0.000015
17	1	0	4.722406	-1.784850	-0.000011
18	1	0	5.508357	0.580208	-0.000021
19	1	0	3.790770	2.411774	-0.000013
20	1	0	1.371004	1.779666	0.000003

Data 14: Cartesian coordinates and energies of the optimized geometry for the *s-trans*-conformer of (*Z*)-2-formylpyridine thiosemicarbazone (the gas phase).

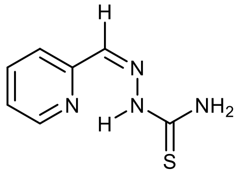

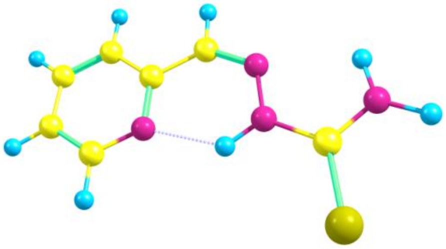

Electronic Energy = -888.907275770 a.u.
Zero-point correction = 0.151825 (Hartree/Particle)
Thermal correction to Energy = 0.162581
Thermal correction to Enthalpy = 0.163525
Thermal correction to Gibbs Free Energy = 0.114097
Sum of electronic and zero-point Energies = -888.755451
Sum of electronic and thermal Energies = -888.744695
Sum of electronic and thermal Enthalpies = -888.743751
Sum of electronic and thermal Free Energies = -888.793178

Standard orientation:

Center Number	Atomic Number	Atomic Type	Coordinates (Angstroms) X	Y	Z
1	7	0	3.352750	1.232371	-0.000010
2	6	0	2.641479	0.089384	-0.000002
3	16	0	3.309919	-1.444462	0.000003
4	7	0	1.273601	0.249257	-0.000003
5	7	0	0.732573	1.479665	0.000002
6	6	0	-0.551314	1.632909	0.000004
7	6	0	-1.616111	0.627305	0.000002
8	7	0	-1.286020	-0.681378	0.000001
9	6	0	-2.265084	-1.587462	-0.000001
10	6	0	-3.617214	-1.254278	-0.000002
11	6	0	-3.964879	0.095783	-0.000001
12	6	0	-2.956907	1.048777	0.000001
13	1	0	2.877856	2.123552	0.000011
14	1	0	4.355810	1.170652	0.000017
15	1	0	0.666987	-0.574100	-0.000006
16	1	0	-0.871957	2.670365	0.000007
17	1	0	-1.950236	-2.626525	-0.000001
18	1	0	-4.371161	-2.031622	-0.000004
19	1	0	-5.005910	0.398165	-0.000002
20	1	0	-3.190242	2.107003	0.000001

Data 15: Cartesian coordinates and energies of the optimized geometry for the *s-trans*-conformer of (*E*)-2-formylpyridine thiosemicarbazone (DMSO solution).

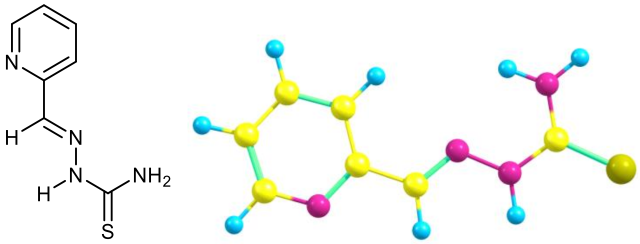

Electronic Energy =	-888.919911607 a.u.
Zero-point correction =	0.151064 (Hartree/Particle)
Thermal correction to Energy =	0.162156
Thermal correction to Enthalpy =	0.163100
Thermal correction to Gibbs Free Energy =	0.112612
Sum of electronic and zero-point Energies =	-888.768847
Sum of electronic and thermal Energies =	-888.757755
Sum of electronic and thermal Enthalpies =	-888.756811
Sum of electronic and thermal Free Energies =	-888.807299

Standard orientation:

Center Number	Atomic Number	Atomic Type	Coordinates (Angstroms) X	Y	Z
1	7	0	-2.717623	1.576292	0.000008
2	6	0	-2.935974	0.257524	-0.000007
3	16	0	-4.478509	-0.448616	0.000014
4	7	0	-1.827995	-0.537936	-0.000004
5	7	0	-0.579445	-0.004332	-0.000004
6	6	0	0.411486	-0.817764	0.000001

7	6	0	1.799823	-0.342262	-0.000003
8	7	0	2.728326	-1.316911	0.000002
9	6	0	4.015227	-0.956666	-0.000001
10	6	0	4.441448	0.369791	-0.000008
11	6	0	3.475508	1.375608	-0.000013
12	6	0	2.134096	1.019233	-0.000010
13	1	0	-1.774504	1.939818	0.000007
14	1	0	-3.502624	2.206044	0.000022
15	1	0	-1.972931	-1.543565	0.000000
16	1	0	0.278039	-1.902014	0.000006
17	1	0	4.739288	-1.765801	0.000003
18	1	0	5.498838	0.603361	-0.000010
19	1	0	3.765088	2.419894	-0.000018
20	1	0	1.352426	1.767536	-0.000013

Data 16: Cartesian coordinates and energies of the optimized geometry for the *s-cis*-conformer of (*E*)-2-formylpyridine thiosemicarbazone (DMSO solution).

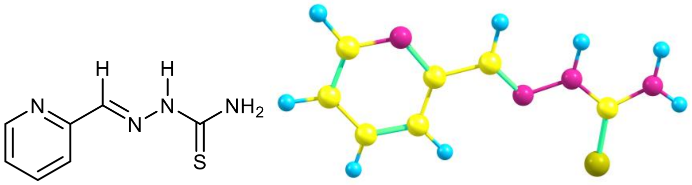

Electronic Energy = -888.911230345 a.u.
Zero-point correction= 0.150613 (Hartree/Particle)
Thermal correction to Energy= 0.161969
Thermal correction to Enthalpy= 0.162913
Thermal correction to Gibbs Free Energy= 0.111671
Sum of electronic and zero-point Energies= -888.760617
Sum of electronic and thermal Energies= -888.749261
Sum of electronic and thermal Enthalpies= -888.748317
Sum of electronic and thermal Free Energies= -888.799560

Standard orientation:

Center Number	Atomic Number	Atomic Type	Coordinates (Angstroms) X	Y	Z
1	7	0	-4.130487	-1.047284	0.000023
2	6	0	-3.059984	-0.229883	-0.000024
3	16	0	-3.224327	1.452494	0.000002
4	7	0	-1.862075	-0.889360	-0.000018
5	7	0	-0.672317	-0.241639	-0.000012
6	6	0	0.381118	-0.970714	-0.000006
7	6	0	1.724856	-0.378338	-0.000002
8	7	0	2.733813	-1.269112	0.000002
9	6	0	3.985220	-0.799277	0.000006
10	6	0	4.295762	0.558636	0.000007
11	6	0	3.246423	1.477387	0.000003
12	6	0	1.940673	1.007024	-0.000002
13	1	0	-4.058490	-2.054941	0.000042
14	1	0	-5.049722	-0.636865	0.000056
15	1	0	-1.859996	-1.907882	-0.000013
16	1	0	0.343549	-2.063364	-0.000005
17	1	0	4.776163	-1.543222	0.000010
18	1	0	5.329138	0.882396	0.000011
19	1	0	3.445152	2.542735	0.000004
20	1	0	1.096496	1.683990	-0.000005

Data 17: Cartesian coordinates and energies of the optimized geometry for the *s*-*trans*-conformer of (*Z*)-2-formylpyridine thiosemicarbazone (DMSO solution).

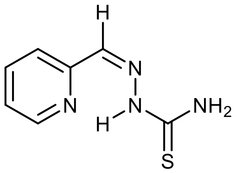

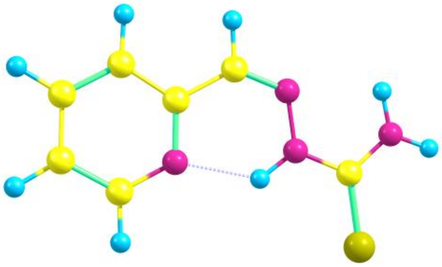

Electronic Energy =	-888.922156341 a.u.
Zero-point correction =	0.151750 (Hartree/Particle)
Thermal correction to Energy =	0.162479
Thermal correction to Enthalpy =	0.163423
Thermal correction to Gibbs Free Energy =	0.114031
Sum of electronic and zero-point Energies =	-888.770407
Sum of electronic and thermal Energies =	-888.759678
Sum of electronic and thermal Enthalpies =	-888.758734
Sum of electronic and thermal Free Energies =	-888.808126

Standard orientation:

Center Number	Atomic Number	Atomic Type	Coordinates (Angstroms) X	Y	Z
1	7	0	-3.354941	-1.222205	-0.000002
2	6	0	-2.623073	-0.103060	0.000000
3	16	0	-3.291342	1.458426	-0.000000
4	7	0	-1.269228	-0.260841	0.000001
5	7	0	-0.730347	-1.506926	0.000003
6	6	0	0.552739	-1.643983	0.000003
7	6	0	1.611545	-0.627166	0.000001
8	7	0	1.271881	0.680435	-0.000001
9	6	0	2.248054	1.590967	-0.000003
10	6	0	3.601974	1.259578	-0.000003
11	6	0	3.956139	-0.087415	-0.000002
12	6	0	2.950175	-1.045724	0.000000
13	1	0	-2.900226	-2.125272	0.000006
14	1	0	-4.359121	-1.157757	0.000007
15	1	0	-0.644015	0.551238	-0.000001
16	1	0	0.886856	-2.676953	0.000005
17	1	0	1.934210	2.629779	-0.000004
18	1	0	4.352316	2.039979	-0.000005
19	1	0	4.997743	-0.385562	-0.000002
20	1	0	3.186845	-2.102676	0.000002

Data 18: Cartesian coordinates and energies of the optimized geometry for the *s*-*cis*-conformer of (*Z*)-2-formylpyridine thiosemicarbazone (DMSO solution).

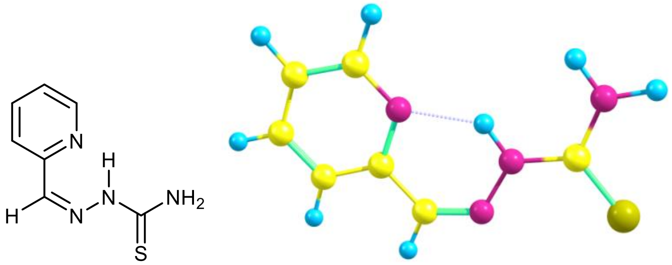

Electronic Energy =	-888.914231651 a.u.
Zero-point correction =	0.151507 (Hartree/Particle)
Thermal correction to Energy =	0.162382
Thermal correction to Enthalpy =	0.163326
Thermal correction to Gibbs Free Energy =	0.113630
Sum of electronic and zero-point Energies =	-888.762725
Sum of electronic and thermal Energies =	-888.751849
Sum of electronic and thermal Enthalpies =	-888.750905
Sum of electronic and thermal Free Energies =	-888.800602

Standard orientation:

Center Number	Atomic Number	Atomic Type	Coordinates (Angstroms) X	Y	Z
1	7	0	2.696044	1.820786	-0.000001
2	6	0	2.525585	0.483940	0.000001
3	16	0	3.847473	-0.574243	-0.000003
4	7	0	1.214427	0.101239	0.000004
5	7	0	0.856849	-1.204860	0.000002
6	6	0	-0.398699	-1.502650	0.000001
7	6	0	-1.582958	-0.633044	0.000000
8	7	0	-1.415401	0.707772	-0.000001
9	6	0	-2.499605	1.487431	-0.000002
10	6	0	-3.799162	0.985251	-0.000002
11	6	0	-3.977117	-0.396452	-0.000000
12	6	0	-2.857292	-1.218606	0.000001
13	1	0	1.924127	2.472242	0.000018
14	1	0	3.632619	2.189648	0.000011
15	1	0	0.446011	0.788985	0.000006
16	1	0	-0.596799	-2.570236	0.000002
17	1	0	-2.320305	2.557578	-0.000003
18	1	0	-4.643697	1.662516	-0.000002
19	1	0	-4.971942	-0.825567	0.000000
20	1	0	-2.957525	-2.297054	0.000002

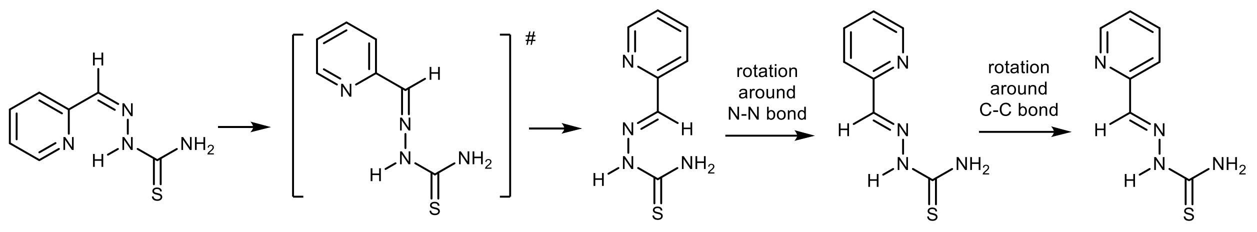

Z-isomer **TS** Conformer **A** Conformer **B** *E*-isomer

The gas phase

Data 19: Cartesian coordinates, energy, imaginary frequency of the transition state for the transformation of the most stable conformer of (*Z*)-2-formylpyridine thiosemicarbazone into conformer **A** of the (*E*)-isomer (the gas phase).

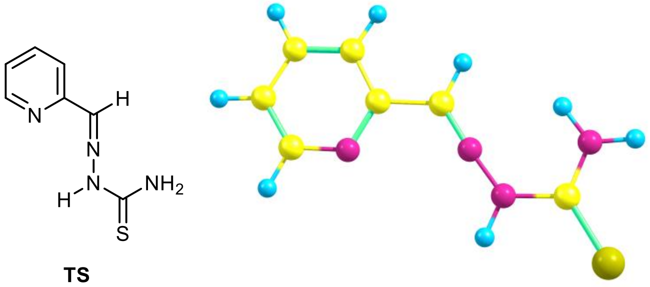

TS

Imaginary Frequency =	-389.4919 cm^{-1}
Electronic Energy =	-888.848199567 a.u.
Zero-point correction =	0.149264 (Hartree/Particle)
Thermal correction to Energy =	0.160171
Thermal correction to Enthalpy =	0.161115
Thermal correction to Gibbs Free Energy =	0.111157
Sum of electronic and zero-point Energies =	-888.698936
Sum of electronic and thermal Energies =	-888.688029
Sum of electronic and thermal Enthalpies =	-888.687085
Sum of electronic and thermal Free Energies =	-888.737042

Standard orientation:

Center Number	Atomic Number	Atomic Type	Coordinates (Angstroms) X	Y	Z
1	7	0	2.965830	1.497613	0.000000
2	6	0	2.980319	0.153637	0.000000
3	16	0	4.336690	-0.808205	0.000000
4	7	0	1.705393	-0.432123	0.000000
5	7	0	0.594941	0.208011	-0.000000
6	6	0	-0.442777	0.917679	-0.000000
7	6	0	-1.815759	0.349077	-0.000000
8	7	0	-1.945175	-0.987080	-0.000000
9	6	0	-3.176507	-1.493810	-0.000000
10	6	0	-4.336982	-0.716764	0.000000
11	6	0	-4.199865	0.667919	0.000000
12	6	0	-2.919823	1.210787	0.000000
13	1	0	2.094148	2.004995	-0.000001
14	1	0	3.841828	1.990956	-0.000001
15	1	0	1.704933	-1.451068	0.000000

16	1	0	-0.406909	2.020878	-0.000001
17	1	0	-3.245728	-2.578534	-0.000000
18	1	0	-5.312012	-1.188869	0.000000
19	1	0	-5.072307	1.311808	0.000001
20	1	0	-2.769549	2.285015	0.000000

Data 20: The intrinsic reaction coordinate analysis for the transformation of the most stable conformer of (*Z*)-2-formylpyridine thiosemicarbazone into conformer **A** of the (*E*)-isomer (the gas phase).

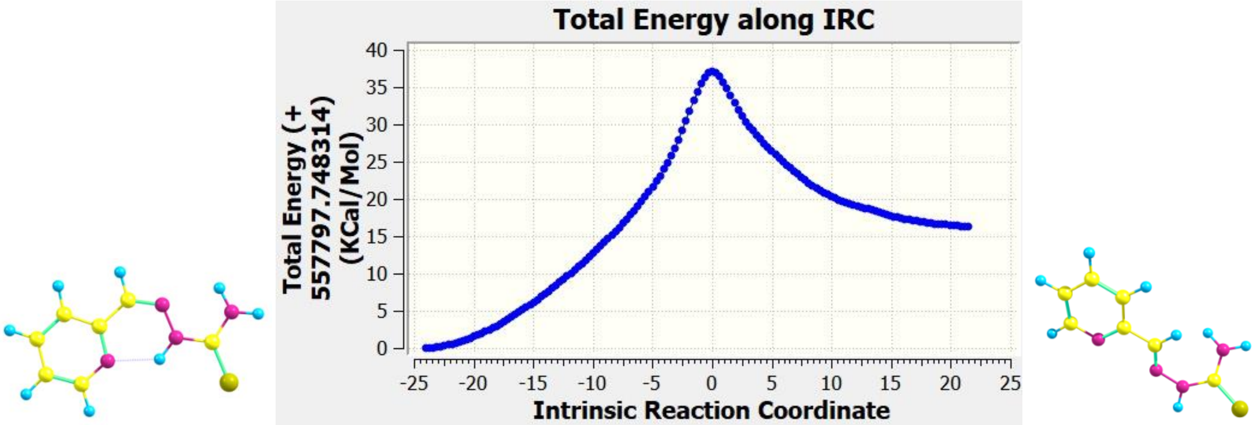

Data 21: Cartesian coordinates and energies of the optimized geometry for the conformer **A** of (*E*)-2-formylpyridine thiosemicarbazone (the gas phase).

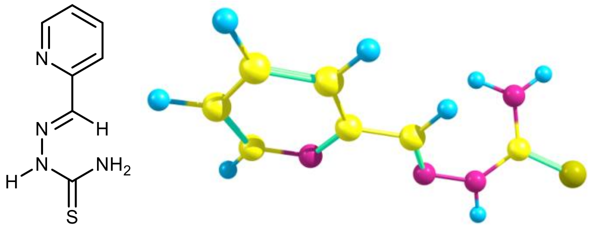

Electronic Energy =	-888.881488140 a.u.
Zero-point correction =	0.151444 (Hartree/Particle)
Thermal correction to Energy =	0.162429
Thermal correction to Enthalpy =	0.163374
Thermal correction to Gibbs Free Energy =	0.112737
Sum of electronic and zero-point Energies =	-888.730044
Sum of electronic and thermal Energies =	-888.719059
Sum of electronic and thermal Enthalpies =	-888.718115
Sum of electronic and thermal Free Energies =	-888.768751

Standard orientation:

Center Number	Atomic Number	Atomic Type	Coordinates (Angstroms) X	Y	Z
1	7	0	-2.356166	1.250978	0.756314
2	6	0	-2.810104	0.126306	0.130661
3	16	0	-4.418003	-0.105958	-0.260216

4	7	0	-1.885383	-0.850278	-0.171453
5	7	0	-0.524587	-0.919550	-0.061724
6	6	0	0.203235	0.132558	-0.162203
7	6	0	1.671161	0.054193	-0.078780
8	7	0	2.250147	-1.110859	0.254459
9	6	0	3.578004	-1.152979	0.306566
10	6	0	4.404361	-0.057224	0.041213
11	6	0	3.804036	1.150336	-0.296542
12	6	0	2.415847	1.210158	-0.355933
13	1	0	-1.512521	1.203954	1.311998
14	1	0	-3.088278	1.846683	1.112339
15	1	0	-2.298364	-1.743152	-0.397569
16	1	0	-0.207978	1.120654	-0.368922
17	1	0	4.012499	-2.111268	0.578849
18	1	0	5.481401	-0.155776	0.103343
19	1	0	4.402297	2.028816	-0.509940
20	1	0	1.911681	2.133299	-0.619716

Data 21: Cartesian coordinates and energies of the optimized geometry for the conformer **B** of (*E*)-2-formylpyridine thiosemicarbazone (the gas phase).

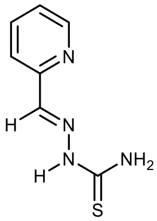

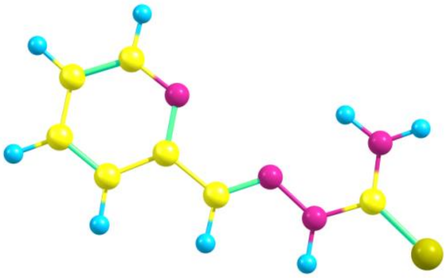

Electronic Energy =	-888.900127943 a.u.
Zero-point correction =	0.150988 (Hartree/Particle)
Thermal correction to Energy =	0.162051
Thermal correction to Enthalpy =	0.162995
Thermal correction to Gibbs Free Energy =	0.112568
Sum of electronic and zero-point Energies =	-888.749140
Sum of electronic and thermal Energies =	-888.738077
Sum of electronic and thermal Enthalpies =	-888.737133
Sum of electronic and thermal Free Energies =	-888.787560

Standard orientation:

Center Number	Atomic Number	Atomic Type	Coordinates (Angstroms) X	Y	Z
1	7	0	-2.681565	1.575982	0.000003
2	6	0	-2.945576	0.258133	-0.000000
3	16	0	-4.474355	-0.421858	-0.000003
4	7	0	-1.831536	-0.553033	0.000003
5	7	0	-0.590195	-0.031995	0.000002
6	6	0	0.409701	-0.833773	0.000002
7	6	0	1.796720	-0.354545	0.000001
8	7	0	2.027911	0.969899	-0.000000
9	6	0	3.292873	1.380158	-0.000001
10	6	0	4.393060	0.518432	-0.000002
11	6	0	4.152516	-0.851057	-0.000001
12	6	0	2.835604	-1.296996	0.000000
13	1	0	-1.724493	1.902172	0.000002

14	1	0	-3.454868	2.218010	-0.000000
15	1	0	-2.004097	-1.554967	-0.000001
16	1	0	0.265567	-1.919364	0.000002
17	1	0	3.443150	2.456576	-0.000002
18	1	0	5.400523	0.916396	-0.000003
19	1	0	4.972819	-1.559776	-0.000001
20	1	0	2.609382	-2.357400	0.000001

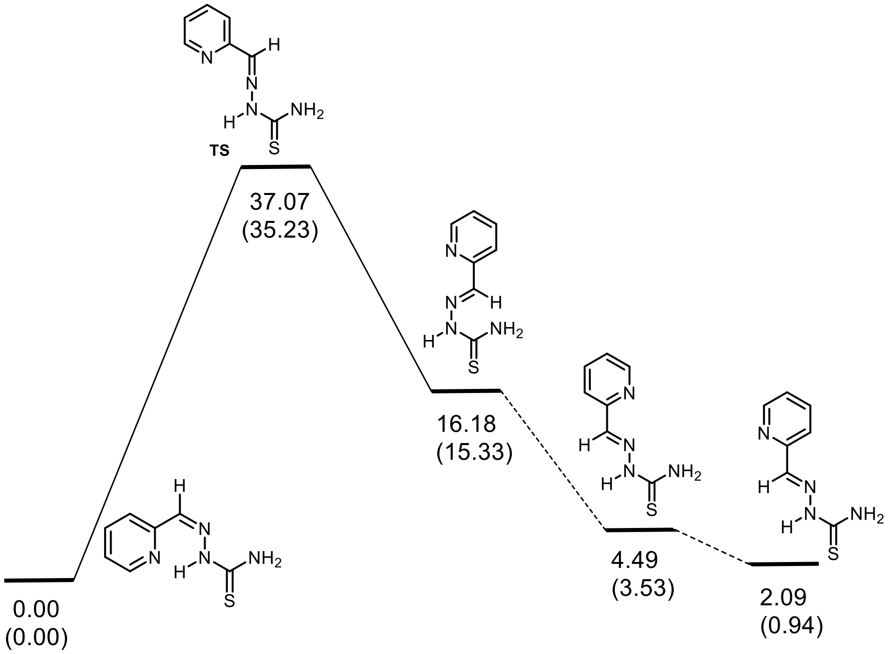

Figure S14. Electronic energy and Gibbs free energy profiles (in kcal/mol) for the transformation of the most stable conformer of (*Z*)-2-formylpyridine thiosemicarbazone into the most stable conformer of (*E*)-2-formylpyridine thiosemicarbazone in the gas phase. Free energies (in parentheses) at 298 K and 1 atm.

<u>DMSO solution</u>

Data 22: Cartesian coordinates, energy, imaginary frequency of the transition state for the transformation of the most stable conformer of (*Z*)-2-formylpyridine thiosemicarbazone into conformer **A** of the (*E*)-isomer (DMSO solution).

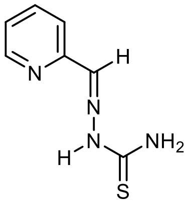

TS

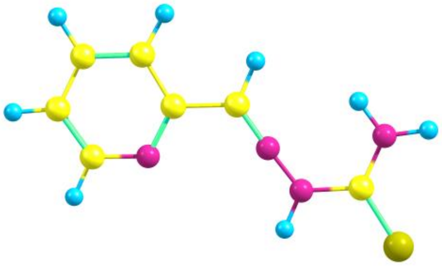

Imaginary Frequency = -414.4982 cm^{-1}

Electronic Energy = -888.86340949 a.u.

Zero-point correction = 0.149585 (Hartree/Particle)

Thermal correction to Energy = 0.160351
Thermal correction to Enthalpy = 0.161295
Thermal correction to Gibbs Free Energy = 0.111633
Sum of electronic and zero-point Energies = -888.713825
Sum of electronic and thermal Energies = -888.703059
Sum of electronic and thermal Enthalpies = -888.702115
Sum of electronic and thermal Free Energies = -888.751777

Standard orientation:

Center Number	Atomic Number	Atomic Type	Coordinates (Angstroms) X	Y	Z
1	7	0	-2.985819	1.496245	0.000000
2	6	0	-2.975101	0.165038	0.000000
3	16	0	-4.337983	-0.825007	0.000000
4	7	0	-1.708226	-0.419986	0.000000
5	7	0	-0.601707	0.233207	0.000000
6	6	0	0.449146	0.925383	0.000000
7	6	0	1.818901	0.346209	-0.000000
8	7	0	1.949694	-0.991850	-0.000000
9	6	0	3.184168	-1.498589	-0.000000
10	6	0	4.342405	-0.718836	-0.000000
11	6	0	4.203748	0.666057	-0.000000
12	6	0	2.922462	1.208000	-0.000000
13	1	0	-2.122289	2.021070	0.000000
14	1	0	-3.863291	1.990142	0.000000
15	1	0	-1.702486	-1.438909	0.000000
16	1	0	0.422023	2.025240	0.000000
17	1	0	3.258066	-2.582561	-0.000000
18	1	0	5.317490	-1.190032	-0.000000
19	1	0	5.074986	1.310770	-0.000000
20	1	0	2.771264	2.281520	0.000000

Data 23: The intrinsic reaction coordinate analysis for the transformation of the most stable conformer of (*Z*)-2-formylpyridine thiosemicarbazone into conformer **A** of the (*E*)-isomer (DMSO solution).

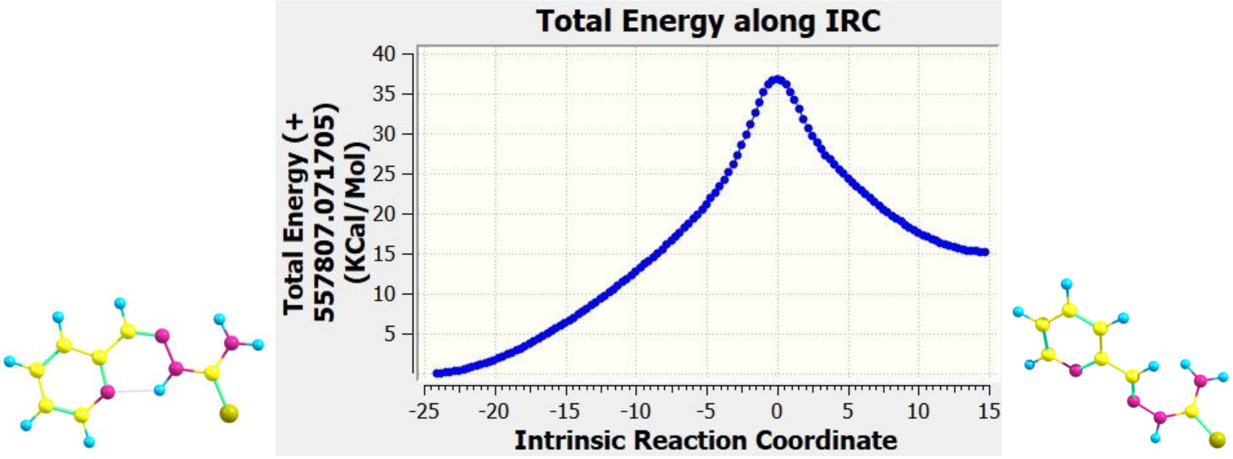

Data 24: Cartesian coordinates and energies of the optimized geometry for the conformer **A** of (*E*)-2-formylpyridine thiosemicarbazone (DMSO solution).

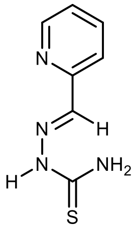

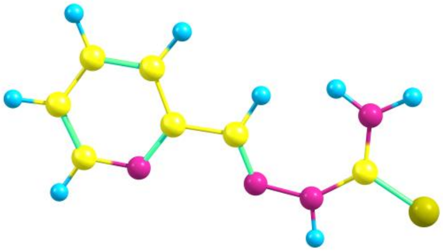

Electronic Energy =	-888.897966374 a.u.
Zero-point correction =	0.150811 (Hartree/Particle)
Thermal correction to Energy =	0.161452
Thermal correction to Enthalpy =	0.162396
Thermal correction to Gibbs Free Energy =	0.112766
Sum of electronic and zero-point Energies =	-888.747156
Sum of electronic and thermal Energies =	-888.736515
Sum of electronic and thermal Enthalpies =	-888.735570
Sum of electronic and thermal Free Energies =	-888.785200

Standard orientation:

Center Number	Atomic Number	Atomic Type	Coordinates (Angstroms) X	Y	Z
1	7	0	-2.864719	-0.820168	-0.000000
2	6	0	-2.123072	-1.933289	-0.000000
3	16	0	-2.837293	-3.476619	-0.000000
4	7	0	-0.748796	-1.871613	-0.000000
5	7	0	0.205122	-0.900508	-0.000000
6	6	0	-0.000000	0.363051	0.000000
7	6	0	1.134085	1.308227	0.000000
8	7	0	2.392453	0.833437	0.000000
9	6	0	3.391405	1.715387	0.000000
10	6	0	3.206029	3.100291	0.000000
11	6	0	1.904518	3.588995	0.000000
12	6	0	0.850487	2.680380	0.000000
13	1	0	-2.499441	0.115232	0.000000
14	1	0	-3.867145	-0.926229	-0.000000
15	1	0	-0.326615	-2.788951	-0.000000
16	1	0	-0.968809	0.853351	0.000000
17	1	0	4.395536	1.300964	0.000000
18	1	0	4.060745	3.765154	0.000000
19	1	0	1.710274	4.654982	0.000000
20	1	0	-0.177001	3.025125	0.000000

Data 25: Cartesian coordinates and energies of the optimized geometry for the conformer **B** of (*E*)-2-formylpyridine thiosemicarbazone (DMSO solution).

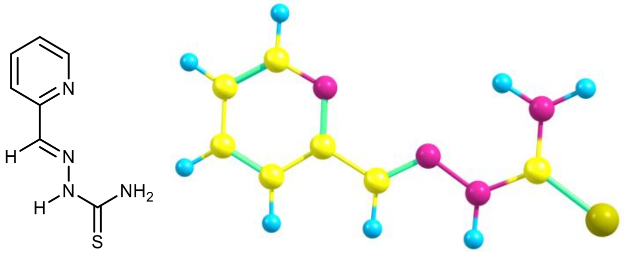

Electronic Energy =	-888.917947875 a.u.
Zero-point correction =	0.151125 (Hartree/Particle)
Thermal correction to Energy =	0.162198
Thermal correction to Enthalpy =	0.163142
Thermal correction to Gibbs Free Energy =	0.112708
Sum of electronic and zero-point Energies =	-888.766823
Sum of electronic and thermal Energies =	-888.755750
Sum of electronic and thermal Enthalpies =	-888.754806
Sum of electronic and thermal Free Energies =	-888.805240

Standard orientation:

Center Number	Atomic Number	Atomic Type	Coordinates (Angstroms) X	Y	Z
1	7	0	-2.727373	1.579333	0.000001
2	6	0	-2.942013	0.260090	-0.000003
3	16	0	-4.482412	-0.451424	0.000002
4	7	0	-1.830685	-0.530703	-0.000002
5	7	0	-0.585746	0.012272	-0.000002
6	6	0	0.407138	-0.798590	-0.000000
7	6	0	1.801646	-0.337079	-0.000000
8	7	0	2.057565	0.984829	-0.000001
9	6	0	3.332481	1.371438	0.000000
10	6	0	4.413525	0.485584	0.000001
11	6	0	4.146643	-0.878782	0.000001
12	6	0	2.820079	-1.299289	0.000001
13	1	0	-1.783987	1.942872	0.000001
14	1	0	-3.513559	2.207549	0.000004
15	1	0	-1.971865	-1.537094	-0.000001
16	1	0	0.254588	-1.880815	-0.000001
17	1	0	3.507749	2.443456	-0.000000
18	1	0	5.428133	0.863888	0.000001
19	1	0	4.952269	-1.603356	0.000002
20	1	0	2.571949	-2.354056	0.000001

Pathway 2: *via tautomeric shift*

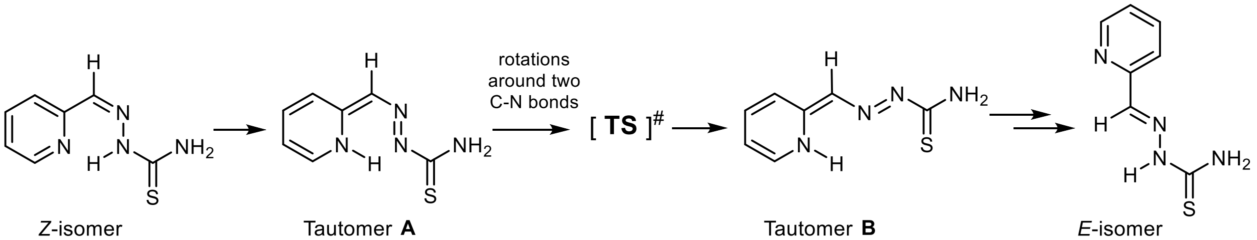

Data 26: Cartesian coordinates and energies of the optimized geometry for the tautomer **A** formed via tautomeric shift in *s*-*trans*-conformer of (*Z*)-2-formylpyridine thiosemicarbazone (the gas phase).

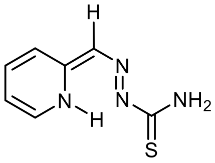

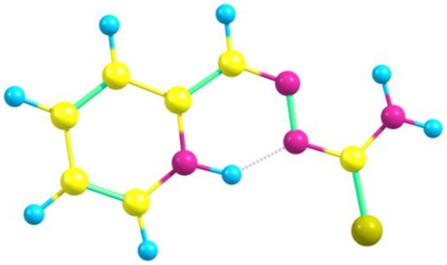

Electronic Energy =	-888.8852658404 a.u.
Zero-point correction =	0.150269 (Hartree/Particle)
Thermal correction to Energy =	0.160867
Thermal correction to Enthalpy =	0.161811
Thermal correction to Gibbs Free Energy =	0.112901
Sum of electronic and zero-point Energies =	-888.734997
Sum of electronic and thermal Energies =	-888.724399
Sum of electronic and thermal Enthalpies =	-888.723455
Sum of electronic and thermal Free Energies =	-888.772365

Standard orientation:

Center Number	Atomic Number	Atomic Type	Coordinates (Angstroms) X	Y	Z
1	7	0	-3.328544	-1.188378	-0.000003
2	6	0	-2.550630	-0.086955	0.000001
3	16	0	-3.163957	1.475238	-0.000001
4	7	0	-1.175554	-0.265794	0.000003
5	7	0	-0.770731	-1.517454	0.000006
6	6	0	0.539792	-1.732737	0.000005
7	6	0	1.559622	-0.739482	0.000001
8	7	0	1.200273	0.582147	0.000001
9	6	0	2.092972	1.592671	-0.000002
10	6	0	3.441755	1.336077	-0.000005
11	6	0	3.866706	-0.011252	-0.000005
12	6	0	2.945464	-1.032573	-0.000001
13	1	0	-2.891987	-2.099465	0.000003
14	1	0	-4.327880	-1.082147	0.000001
15	1	0	0.848307	-2.770547	0.000007
16	1	0	1.675192	2.591108	-0.000001
17	1	0	4.151187	2.151905	-0.000008
18	1	0	4.926153	-0.240043	-0.000007
19	1	0	3.257462	-2.069178	-0.000001
20	1	0	0.132675	0.686420	0.000004

Data 27: Cartesian coordinates, energy, imaginary frequency of the transition state for the transformation of the tautomer **A** into the tautomer **B** (the gas phase).

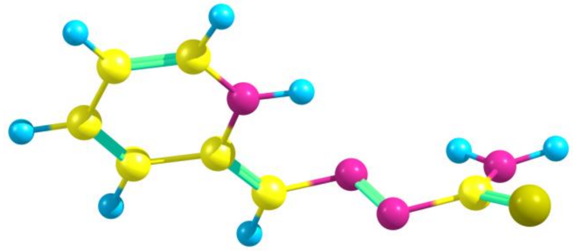

Imaginary Frequency =	-519.9298 cm^{-1}
Electronic Energy =	-888.829551222 a.u.
Zero-point correction =	0.148768 (Hartree/Particle)
Thermal correction to Energy =	0.159665
Thermal correction to Enthalpy =	0.160610
Thermal correction to Gibbs Free Energy =	0.109717
Sum of electronic and zero-point Energies =	-888.680783
Sum of electronic and thermal Energies =	-888.669886
Sum of electronic and thermal Enthalpies =	-888.668942
Sum of electronic and thermal Free Energies =	-888.719834

Standard orientation:

Center Number	Atomic Number	Atomic Type	Coordinates (Angstroms) X	Y	Z
1	7	0	-3.253799	-0.936688	1.023174
2	6	0	-2.813325	-0.124869	0.052076
3	16	0	-3.583918	1.198469	-0.562451
4	7	0	-1.523356	-0.474764	-0.522372
5	7	0	-0.774763	-1.058262	0.284785
6	6	0	0.514532	-1.425210	-0.233039
7	6	0	1.601274	-0.625903	-0.111951
8	7	0	1.508920	0.683542	0.389800
9	6	0	2.587452	1.516014	0.547240
10	6	0	3.839015	1.119526	0.217436
11	6	0	4.005819	-0.206581	-0.320572
12	6	0	2.945073	-1.033211	-0.479104
13	1	0	-2.599089	-1.587714	1.439544
14	1	0	-4.118286	-0.718136	1.492070
15	1	0	0.588179	-2.441093	-0.596501
16	1	0	2.359419	2.496926	0.943722
17	1	0	4.677726	1.787944	0.346960
18	1	0	4.996642	-0.544668	-0.601992
19	1	0	3.067873	-2.030413	-0.883081
20	1	0	0.592176	1.046264	0.598270

Data 28: Cartesian coordinates and energies of the optimized geometry for the tautomer **B** formed from the tautomer **A** (the gas phase).

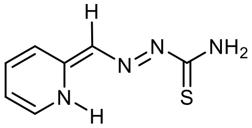

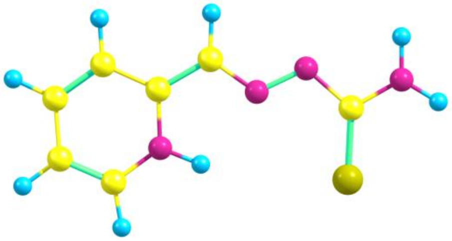

Electronic Energy = -888.876986628 a.u.
Zero-point correction = 0.151034 (Hartree/Particle)
Thermal correction to Energy = 0.161938
Thermal correction to Enthalpy = 0.162883
Thermal correction to Gibbs Free Energy = 0.112987
Sum of electronic and zero-point Energies = -888.725952
Sum of electronic and thermal Energies = -888.715048
Sum of electronic and thermal Enthalpies = -888.714104
Sum of electronic and thermal Free Energies = -888.764000

Standard orientation:

Center Number	Atomic Number	Atomic Type	Coordinates (Angstroms) X	Y	Z
1	7	0	-4.118575	-0.829948	-0.000008
2	6	0	-2.932777	-0.179586	-0.000000
3	16	0	-2.836387	1.497573	0.000007
4	7	0	-1.886599	-1.114137	0.000002
5	7	0	-0.723313	-0.576802	0.000002
6	6	0	0.357087	-1.349003	0.000004
7	6	0	1.584646	-0.659442	-0.000003
8	7	0	1.551196	0.717070	-0.000011
9	6	0	2.650689	1.503200	-0.000006
10	6	0	3.901749	0.947431	0.000002
11	6	0	4.000991	-0.467527	0.000004
12	6	0	2.875936	-1.252434	0.000001
13	1	0	-4.127215	-1.839041	-0.000019
14	1	0	-4.975450	-0.303777	-0.000013
15	1	0	0.319064	-2.435826	0.000008
16	1	0	2.469430	2.569887	-0.000012
17	1	0	4.779039	1.578110	0.000006
18	1	0	4.979780	-0.933355	0.000010
19	1	0	2.944765	-2.332616	0.000004
20	1	0	0.603895	1.106346	-0.000006

Data 29: The intrinsic reaction coordinate analysis for the transformation of the tautomer **A** into the tautomer **B** (the gas phase).

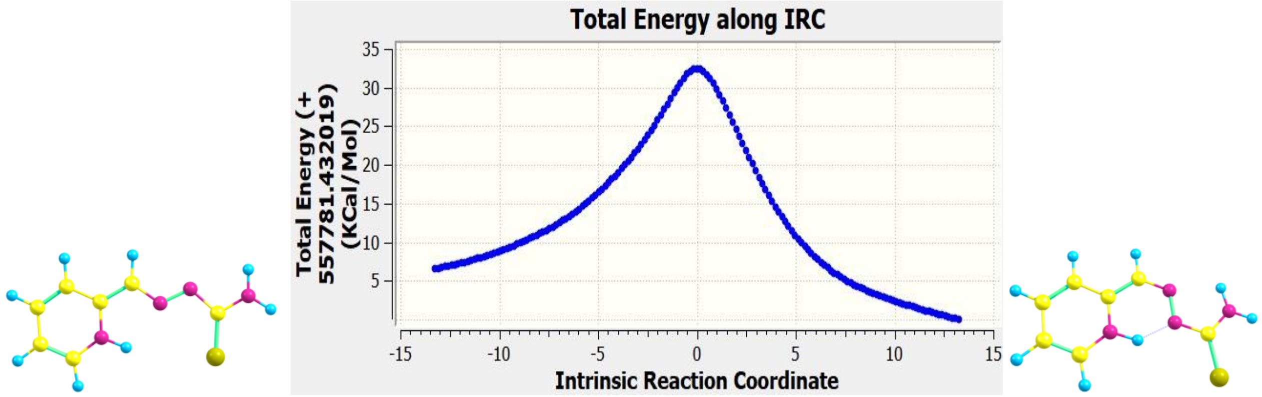

Tautomer **B** Tautomer **A**

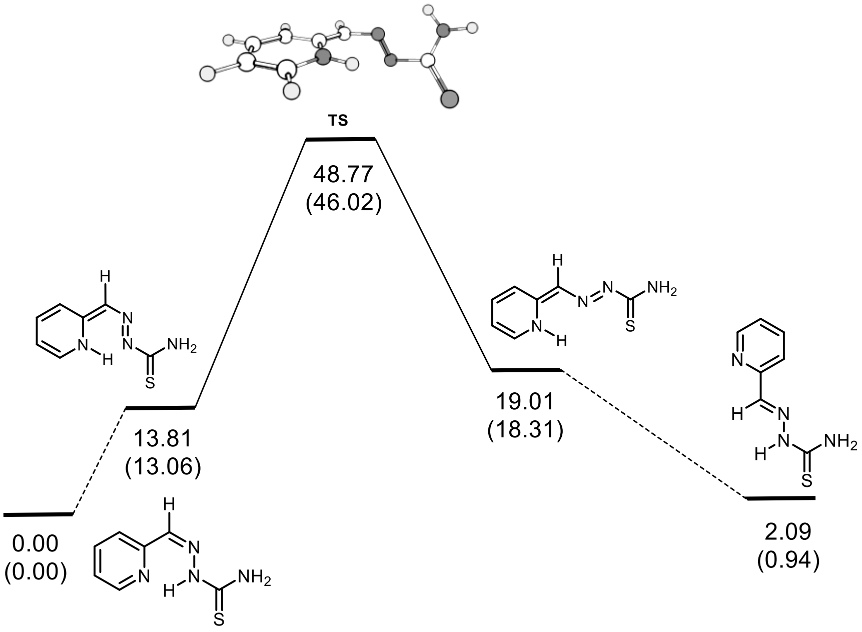

Figure S15. Electronic energy and Gibbs free energy profiles (in kcal/mol) for the transformation of the most stable conformer of (*Z*)-2-formylpyridine thiosemicarbazone into the most stable conformer of (*E*)-2-formylpyridine thiosemicarbazone in the gas phase. Free energies (in parentheses) at 298 K and 1 atm.

Geometry optimization of some stereoisomers and tautomers of H$L^{1c'}$ (DMSO solution)

Data 30: Cartesian coordinates and energies of the optimized geometry for the *s-cis*-conformer (around the exocyclic C-N bond) of (*E*)-**H*L***$^{1c'}$ (DMSO solution).

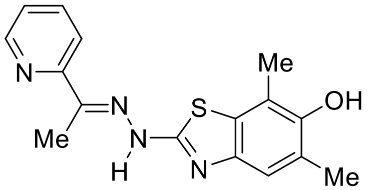

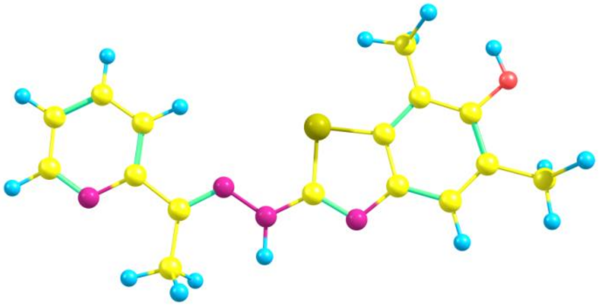

Electronic Energy =	-1312.03278232 a.u.
Zero-point correction =	0.294370 (Hartree/Particle)
Thermal correction to Energy =	0.315455
Thermal correction to Enthalpy =	0.316400
Thermal correction to Gibbs Free Energy =	0.242121
Sum of electronic and zero-point Energies =	-1311.738412
Sum of electronic and thermal Energies =	-1311.717327
Sum of electronic and thermal Enthalpies =	-1311.716383
Sum of electronic and thermal Free Energies =	-1311.790661

Standard orientation:

Center Number	Atomic Number	Atomic Type	Coordinates (Angstroms) X	Y	Z
1	6	0	-4.682881	0.712121	-0.000273
2	6	0	-4.914397	-0.678482	-0.001004
3	6	0	-3.821036	-1.540094	-0.000631
4	6	0	-2.519794	-1.030147	0.000239
5	6	0	-2.331651	0.366295	0.001174
6	6	0	-3.396275	1.270489	0.001767
7	6	0	-6.327816	-1.203236	-0.002423
8	6	0	-3.201235	2.766733	0.001988
9	7	0	-1.357156	-1.793646	0.000520
10	6	0	-0.313353	-1.028133	0.001255
11	16	0	-0.598220	0.724366	0.001568
12	8	0	-5.805079	1.508537	-0.002297
13	7	0	0.968856	-1.505303	0.001432
14	7	0	2.008329	-0.643601	0.000984
15	1	0	-5.557939	2.440092	-0.000578
16	1	0	1.092122	-2.512992	0.000244
17	6	0	3.211064	-1.115437	0.000254
18	6	0	3.526957	-2.585487	-0.000061
19	6	0	4.309882	-0.112675	-0.000336
20	6	0	4.043812	1.267834	-0.001395
21	6	0	5.106137	2.158655	-0.001904
22	6	0	6.410095	1.660986	-0.001397
23	6	0	6.581181	0.280949	-0.000408
24	7	0	5.565817	-0.590418	0.000106
25	1	0	-3.973911	-2.613502	-0.001117
26	1	0	-6.333046	-2.294252	-0.001819
27	1	0	-6.882488	-0.856543	0.874649
28	1	0	-6.880367	-0.857577	-0.881253
29	1	0	-2.144704	3.032473	0.038606

30	1	0	-3.678534	3.237182	0.868929
31	1	0	-3.617373	3.229446	-0.899930
32	1	0	4.601888	-2.740253	-0.000541
33	1	0	3.104839	-3.074987	-0.884981
34	1	0	3.105583	-3.075216	0.885080
35	1	0	3.020899	1.617682	-0.001824
36	1	0	4.922775	3.227012	-0.002729
37	1	0	7.268736	2.320859	-0.001793
38	1	0	7.578611	-0.149024	-0.000015

Data 31: Cartesian coordinates and energies of the optimized geometry for the *s-trans*-conformer (around the exocyclic C-N bond) of (*E*)-**H***L*$^{1c'}$ (DMSO solution).

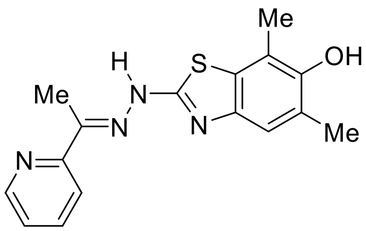

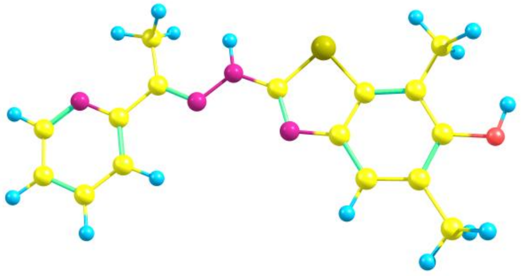

Electronic Energy =	-1312.02804160 a.u.
Zero-point correction =	0.294415 (Hartree/Particle)
Thermal correction to Energy =	0.315485
Thermal correction to Enthalpy =	0.316429
Thermal correction to Gibbs Free Energy =	0.242521
Sum of electronic and zero-point Energies =	-1311.733626
Sum of electronic and thermal Energies =	-1311.712557
Sum of electronic and thermal Enthalpies =	-1311.711612
Sum of electronic and thermal Free Energies =	-1311.785520

Standard orientation:

Center Number	Atomic Number	Atomic Type	Coordinates (Angstroms) X	Y	Z
1	6	0	-4.773395	0.534649	-0.000016
2	6	0	-4.093151	1.769325	0.000009
3	6	0	-2.701030	1.768454	0.000027
4	6	0	-1.993837	0.563171	0.000020
5	6	0	-2.713812	-0.647993	-0.000005
6	6	0	-4.109270	-0.700725	-0.000023
7	6	0	-4.877312	3.056859	0.000016
8	6	0	-4.880865	-1.997080	-0.000050
9	7	0	-0.607583	0.447187	0.000039
10	6	0	-0.252306	-0.794194	0.000027
11	16	0	-1.574662	-1.997280	-0.000010
12	8	0	-6.147255	0.605371	-0.000032
13	7	0	1.033971	-1.272778	0.000047
14	7	0	2.088894	-0.424904	0.000019
15	1	0	-6.532004	-0.278308	-0.000049
16	1	0	1.176014	-2.276127	0.000035
17	6	0	3.277495	-0.931572	0.000023
18	6	0	3.559744	-2.409354	0.000056
19	6	0	4.403121	0.042661	-0.000000
20	6	0	4.171891	1.429417	0.000025
21	6	0	5.256198	2.293342	0.000001
22	6	0	6.547263	1.763083	-0.000047
23	6	0	6.683685	0.379209	-0.000067

24	7	0	5.646660	-0.466380	-0.000044
25	1	0	-2.156448	2.705933	0.000046
26	1	0	-4.205476	3.916434	0.000040
27	1	0	-5.526345	3.127794	-0.877925
28	1	0	-5.526375	3.127767	0.877936
29	1	0	-4.213808	-2.859246	-0.000062
30	1	0	-5.520342	-2.087387	-0.885221
31	1	0	-5.520351	-2.087418	0.885112
32	1	0	4.630878	-2.588195	0.000031
33	1	0	3.128482	-2.890267	-0.885240
34	1	0	3.128529	-2.890215	0.885403
35	1	0	3.157764	1.803705	0.000063
36	1	0	5.099734	3.365980	0.000020
37	1	0	7.422201	2.401225	-0.000066
38	1	0	7.670066	-0.075562	-0.000104

Data 32: Cartesian coordinates and energies of the optimized geometry for (*E*,*Z*)-**H*L*$^{1c'}$** (DMSO solution).

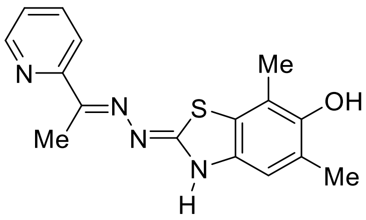

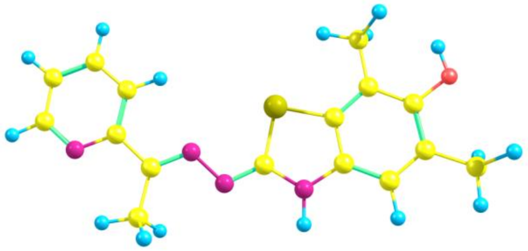

Electronic Energy = -1312.03026828 a.u.
Zero-point correction = 0.294120 (Hartree/Particle)
Thermal correction to Energy = 0.315330
Thermal correction to Enthalpy = 0.316275
Thermal correction to Gibbs Free Energy = 0.241220
Sum of electronic and zero-point Energies = -1311.736149
Sum of electronic and thermal Energies = -1311.714938
Sum of electronic and thermal Enthalpies = -1311.713994
Sum of electronic and thermal Free Energies = -1311.789049

Standard orientation:

Center Number	Atomic Number	Atomic Type	Coordinates (Angstroms) X	Y	Z
1	6	0	4.643360	0.745229	-0.001618
2	6	0	4.905944	-0.636294	0.000197
3	6	0	3.830575	-1.525793	0.000897
4	6	0	2.531772	-1.028976	0.000114
5	6	0	2.295064	0.348496	-0.002231
6	6	0	3.341301	1.271991	-0.004409
7	6	0	6.328818	-1.132585	0.001838
8	6	0	3.109218	2.762076	-0.005206
9	7	0	1.350249	-1.771151	0.000863
10	6	0	0.192676	-1.058499	0.000271
11	16	0	0.547148	0.693265	-0.001588
12	8	0	5.745256	1.568370	0.000729
13	7	0	-0.984243	-1.606206	0.001715
14	7	0	-1.988757	-0.668028	0.001023
15	1	0	5.476690	2.493937	-0.004168
16	6	0	-3.197608	-1.129597	0.002832
17	6	0	-3.525509	-2.597665	0.005425
18	6	0	-4.291036	-0.119574	0.001660
19	6	0	-4.014160	1.259916	0.014818
20	6	0	-5.066559	2.162082	0.012630

21	6	0	-6.375817	1.678203	-0.002625
22	6	0	-6.559843	0.299834	-0.014745
23	7	0	-5.554087	-0.582535	-0.012562
24	1	0	4.010449	-2.594727	0.002240
25	1	0	6.357656	-2.223084	0.000961
26	1	0	6.875319	-0.773202	-0.875074
27	1	0	6.872814	-0.774802	0.880987
28	1	0	2.047929	3.002315	-0.070389
29	1	0	3.597955	3.246520	-0.857684
30	1	0	3.489607	3.230291	0.909333
31	1	0	-4.600825	-2.751691	0.018812
32	1	0	-3.074481	-3.082211	0.875697
33	1	0	-3.098717	-3.080585	-0.878171
34	1	0	-2.987006	1.596352	0.026655
35	1	0	-4.872162	3.228589	0.022910
36	1	0	-7.227966	2.346588	-0.004746
37	1	0	-7.561975	-0.119503	-0.026697
38	1	0	1.326992	-2.781612	0.002548

Data 33: Cartesian coordinates and energies of the optimized geometry for (*E*,*E*)-**H*L*$^{1c'}$** (DMSO solution).

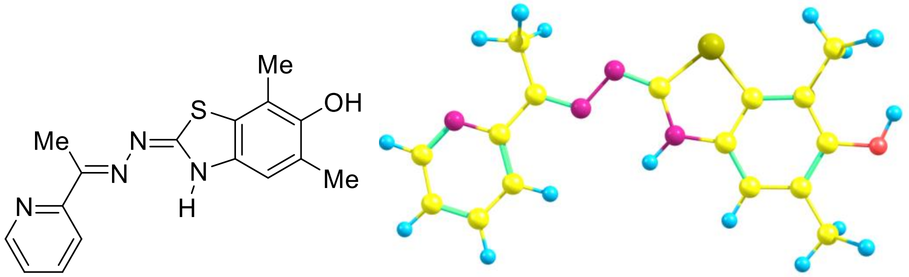

Electronic Energy = -1312.02751003 a.u.
Zero-point correction = 0.294159 (Hartree/Particle)
Thermal correction to Energy = 0.315299
Thermal correction to Enthalpy = 0.316243
Thermal correction to Gibbs Free Energy = 0.241616
Sum of electronic and zero-point Energies = -1311.733351
Sum of electronic and thermal Energies = -1311.712212
Sum of electronic and thermal Enthalpies = -1311.711267
Sum of electronic and thermal Free Energies = -1311.785894

Standard orientation:

Center Number	Atomic Number	Atomic Type	Coordinates (Angstroms) X	Y	Z
1	6	0	4.658551	0.611942	0.007697
2	6	0	3.920983	1.809087	0.018823
3	6	0	2.526579	1.742170	0.017983
4	6	0	1.899657	0.501136	0.006269
5	6	0	2.657144	-0.674749	-0.004332
6	6	0	4.051553	-0.654700	-0.003924
7	6	0	4.637478	3.134689	0.031350
8	6	0	4.882362	-1.912756	-0.015757
9	7	0	0.527604	0.249067	0.004128
10	6	0	0.157803	-1.059971	-0.008165
11	16	0	1.593569	-2.100899	-0.017085
12	8	0	6.027148	0.745900	0.008730
13	7	0	-1.039327	-1.573132	-0.013252
14	7	0	-2.009061	-0.588618	-0.012030
15	1	0	6.452937	-0.118787	0.000783

16	6	0	-3.232586	-1.010475	-0.001348
17	6	0	-3.609407	-2.466717	0.014265
18	6	0	-4.294394	0.033707	-0.003877
19	6	0	-3.978053	1.402496	-0.078658
20	6	0	-5.002389	2.336569	-0.075588
21	6	0	-6.323899	1.894446	0.001839
22	6	0	-6.548149	0.523641	0.071254
23	7	0	-5.569717	-0.388662	0.068252
24	1	0	1.940033	2.653638	0.026359
25	1	0	3.923723	3.959584	0.040392
26	1	0	5.282780	3.228428	0.909712
27	1	0	5.280936	3.246134	-0.846314
28	1	0	4.257833	-2.806298	-0.018968
29	1	0	5.528623	-1.977588	0.866388
30	1	0	5.521195	-1.965630	-0.904102
31	1	0	-4.689273	-2.585273	0.006881
32	1	0	-3.195250	-2.953949	0.901442
33	1	0	-3.176711	-2.975583	-0.851273
34	1	0	-2.943167	1.707932	-0.139239
35	1	0	-4.776732	3.395348	-0.133597
36	1	0	-7.155196	2.588576	0.006835
37	1	0	-7.561276	0.136152	0.131880
38	1	0	-0.190635	0.960826	0.009656

Data 34: Cartesian coordinates and energies of the optimized geometry for the *s-cis*-conformer (around the exocyclic C-N bond) of (*Z*)-**H*L*$^{1c'}$** (DMSO solution).

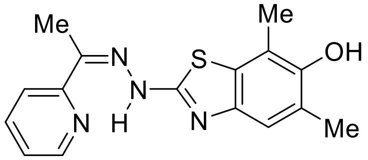

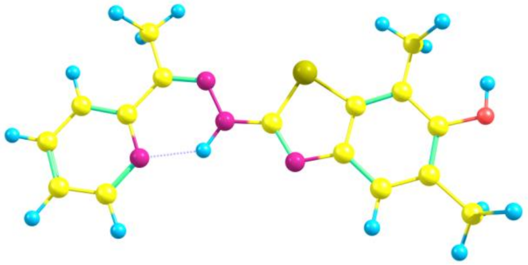

Electronic Energy = -1312.03479280 a.u.
Zero-point correction = 0.294827 (Hartree/Particle)
Thermal correction to Energy = 0.315436
Thermal correction to Enthalpy = 0.316380
Thermal correction to Gibbs Free Energy = 0.244300
Sum of electronic and zero-point Energies = -1311.739966
Sum of electronic and thermal Energies = -1311.719357
Sum of electronic and thermal Enthalpies = -1311.718413
Sum of electronic and thermal Free Energies = -1311.790493
Standard orientation:

Center Number	Atomic Number	Atomic Type	Coordinates (Angstroms) X	Y	Z
1	6	0	4.742819	-0.063880	0.000896
2	6	0	4.437850	-1.440095	-0.000881
3	6	0	3.101570	-1.830961	-0.002471
4	6	0	2.083984	-0.872524	-0.002245
5	6	0	2.431485	0.493239	-0.000755
6	6	0	3.757030	0.933927	-0.000034
7	6	0	5.552425	-2.455591	-0.000477
8	6	0	4.131464	2.395632	0.004540
9	7	0	0.721307	-1.147749	-0.003756
10	6	0	0.034099	-0.048407	-0.003020
11	16	0	0.959008	1.473975	-0.000138

12	8	0	6.082186	0.255059	0.004940
13	7	0	-1.328421	-0.010517	-0.003369
14	7	0	-1.953058	1.184649	-0.003110
15	1	0	6.202848	1.211218	0.001762
16	1	0	-1.899293	-0.866465	-0.002294
17	6	0	-3.248322	1.270639	-0.001886
18	6	0	-3.788691	2.680160	-0.002165
19	6	0	-4.208616	0.141369	-0.000128
20	6	0	-5.594577	0.376561	0.001432
21	6	0	-6.469702	-0.702254	0.003506
22	6	0	-5.954955	-1.996255	0.003981
23	6	0	-4.571891	-2.149994	0.002253
24	7	0	-3.721483	-1.120888	0.000241
25	1	0	2.843377	-2.884106	-0.003848
26	1	0	5.148713	-3.469223	-0.003521
27	1	0	6.197500	-2.340087	-0.876680
28	1	0	6.193467	-2.343880	0.879195
29	1	0	3.249084	3.033593	-0.047545
30	1	0	4.762983	2.655758	-0.852256
31	1	0	4.674307	2.671855	0.915382
32	1	0	-4.407104	2.873687	-0.883932
33	1	0	-2.956754	3.382647	-0.003413
34	1	0	-4.405382	2.874748	0.880570
35	1	0	-5.982968	1.384918	0.001076
36	1	0	-7.540107	-0.533509	0.004760
37	1	0	-6.601959	-2.864185	0.005623
38	1	0	-4.124572	-3.138910	0.002497

Data 35: Cartesian coordinates and energies of the optimized geometry for the *s-trans*-conformer (around the exocyclic C-N bond) of (*Z*)-**H***L*$^{1c'}$ (DMSO solution).

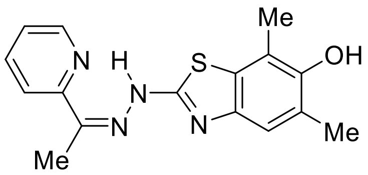

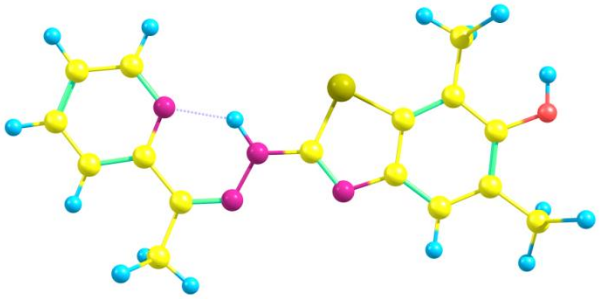

Electronic Energy =	-1312.03016376 a.u.
Zero-point correction =	0.294962 (Hartree/Particle)
Thermal correction to Energy =	0.315536
Thermal correction to Enthalpy =	0.316480
Thermal correction to Gibbs Free Energy =	0.244493
Sum of electronic and zero-point Energies =	-1311.735201
Sum of electronic and thermal Energies =	-1311.714628
Sum of electronic and thermal Enthalpies =	-1311.713683
Sum of electronic and thermal Free Energies =	-1311.785671

Standard orientation:

Center Number	Atomic Number	Atomic Type	Coordinates (Angstroms) X	Y	Z
1	6	0	-4.733494	-0.290988	-0.000004
2	6	0	-4.578378	1.109876	0.000015
3	6	0	-3.291785	1.642004	0.000025

4	6	0	-2.176045	0.800313	0.000015
5	6	0	-2.376888	-0.594843	-0.000005
6	6	0	-3.646287	-1.177359	-0.000012
7	6	0	-5.795151	2.000205	0.000025
8	6	0	-3.862057	-2.670814	-0.000023
9	7	0	-0.852632	1.226191	0.000026
10	6	0	-0.043895	0.216794	0.000011
11	16	0	-0.806999	-1.402796	-0.000016
12	8	0	-6.029973	-0.753093	-0.000005
13	7	0	1.322393	0.253152	0.000020
14	7	0	1.970252	1.440459	-0.000015
15	1	0	-6.045391	-1.716606	-0.000082
16	1	0	1.897585	-0.599748	0.000014
17	6	0	3.267976	1.486287	-0.000024
18	6	0	3.847897	2.880747	-0.000058
19	6	0	4.202768	0.334274	-0.000004
20	6	0	5.593900	0.536475	0.000014
21	6	0	6.443672	-0.562476	0.000030
22	6	0	5.899095	-1.844164	0.000025
23	6	0	4.512944	-1.965765	0.000007
24	7	0	3.687156	-0.916761	-0.000006
25	1	0	-3.147899	2.716633	0.000040
26	1	0	-5.502355	3.051215	0.000012
27	1	0	-6.422283	1.818604	-0.877974
28	1	0	-6.422258	1.818620	0.878045
29	1	0	-2.914844	-3.210280	-0.000022
30	1	0	-4.417733	-3.000366	-0.885119
31	1	0	-4.417743	-3.000374	0.885065
32	1	0	4.470599	3.057969	0.882189
33	1	0	3.035318	3.605398	-0.000097
34	1	0	4.470635	3.057911	-0.882291
35	1	0	6.006135	1.535246	0.000017
36	1	0	7.517711	-0.418691	0.000045
37	1	0	6.525857	-2.726814	0.000036
38	1	0	4.042624	-2.943913	0.000004

References

[1] Kabsch, W. *XDS*. *Acta Crystallogr D Biol Crystallogr* **2010**, *66* (2), 125–132. https://doi.org/10.1107/S0907444909047337.

[2] Sheldrick, G. M. A Short History of *SHELX*. *Acta Crystallogr A Found Crystallogr* **2008**, *64* (1), 112–122. https://doi.org/10.1107/S0108767307043930.

3. Zékány L.; Nagypál I. In *Computational Methods for the Determination of Stability Constants*; Plenum Press: New York, NY, 1985; pp 291–353.

4. Irving, H. M.; Miles, M. G.; Pettit, L. D. A Study of Some Problems in Determining the Stoicheiometric Proton Dissociation Constants of Complexes by Potentiometric Titrations Using a Glass Electrode. *Analyt. Chim. Acta* **1967**, *38*, 475–488.

5. Enyedy, É. A.; Zsigó, É.; Nagy, N. V.; Kowol, C. R.; Roller, A.; Keppler, B. K.; Kiss, T. Complex-Formation Ability of Salicylaldehyde Thiosemicarbazone towards Zn(II), Cu (II), Fe(II), Fe(III) and Ga(III) Ions. *Eur. J. Inorg. Chem.* **2012**, 4036–4047.

6. Hager, S.; Pape, V. F. S.; Pósa, V.; Montsch, B.; Uhlik, L.; Szakács, G.; Tóth, S.; Jabronka, N.; Keppler, B. K.; Kowol, C. R.; Enyedy, E. A.; Heffeter, P. High Copper Complex Stability and Slow Reduction Kinetics as Key Parameters for Improved Activity, Paraptosis Induction and Impact on Drug-Resistant Cells of Anticancer Thiosemicarbazones. *Antioxid.Redox Signal.* **2020**, 33, 395-414.

7. Petrasheuskaya, T. V.; Kiss, M. A.; Dömötör, O.; Holczbauer, T.; May, N. V.; Spengler, G.; Kincses, A.; Čipak Gašparović, A.; Frank, É.; Enyedy, É. A. Salicylaldehyde Thiosemicarbazone Copper Complexes: Impact of Hybridization with Estrone on Cytotoxicity, Solution Stability and Redox Activity. *New J. Chem.* **2020**, *44*, 12154–12168.

8. Kowol, C. R.; Nagy, N. V.; Jakusch, T.; Roller, A.; Heffeter, P.; Keppler, B. K.; Enyedy, É. A. Vanadium(IV/V) Complexes of Triapine and Related Thiosemicarbazones: Synthesis, Solution Equilibrium and Bioactivity. *J. Inorg. Biochem.* **2015**, *152*, 62–73.

9. Aranzaes, J. R.; Daniel, M. C.; Astruc, D. Metallocenes as References for the Determination of Redox Potentials by Cyclic Voltammetry. Permethylated Iron and Cobalt Sandwich Complexes, Inhibition by Polyamine Dendrimers, and the Role of Hydroxy-Containing Ferrocenes. *Can. J. Chem.* **2006**, *84*, 288–299.

10. *GraphPad Software I, GraphPad Prism*; GraphPad Software, Inc., 2007.

11. Mucsi, I.; Varga, A.; Kawase, M.; Motohashi, N.; Molnar, J. Interaction between various resistance modifiers and apoptosis inducer 12H-benzo[alpha]phenothiazine. *Anticancer Res*., 2002, 22, 2833–2836.

12. Ohui, K.; Afanasenko, E.; Bacher, F.; Ting, R. L. X.; Zafar, A.; Blanco-Cabra, N.; Torrents, E.; Dömötör, O.; May, N. V.; Darvasiova, D.; Enyedy, É. A.; Popović-Bijelić, A.; Reynisson, J.; Rapta, P.; Babak, M. V.; Pastorin, G.; Arion, V. B. New Water-Soluble Copper(II) Complexes with Morpholine–Thiosemicarbazone Hybrids: Insights into the Anticancer and Antibacterial Mode of Action. *J. Med. Chem.* **2019**, *62*, 512–530.

13. Popović-Bijelić, A.; Kowol, C. R.; Lind, M. E. S.; Luo, J.; Himo, F.; Enyedy, É. A.; Arion, V. B.; Gräslund, A. Ribonucleotide Reductase Inhibition by Metal Complexes of Triapine (3-Aminopyridine-2-Carboxaldehyde Thiosemicarbazone): A Combined Experimental and Theoretical Study. *J. Inorg. Biochem.* **2011**, *105*, 1422–1431.

14. Zaltariov, M. F.; Hammerstad, M.; Arabshahi, H. J.; Jovanović, K.; Richter, K. W.; Cazacu, M.; Shova, S.; Balan, M.; Andersen, N. H.; Radulović, S.; Reynisson, J.; Andersson, K. K.; Arion, V. B. New Iminodiacetate–Thiosemicarbazone Hybrids and Their Copper(II) Complexes Are Potential

Ribonucleotide Reductase R2 Inhibitors with High Antiproliferative Activity. *Inorg. Chem.* **2017**, *56*, 3532–3549.

15. Becke, A. D. Density-functional thermochemistry. III. The role of exact exchange. *J. Chem. Phys.* **1993**, *98*, 5648–5652.

16. *Gaussian 16, Revision A.03*, M. J. Frisch, G. W. Trucks, H. B. Schlegel, G. E. Scuseria, M. A. Robb, J. R. Cheeseman, G. Scalmani, V. Barone, G. A. Petersson, H. Nakatsuji, X. Li, M. Caricato, A. V. Marenich, J. Bloino, B. G. Janesko, R. Gomperts, B. Mennucci, H. P. Hratchian, J. V. Ortiz, A. F. Izmaylov, J. L. Sonnenberg, D. Williams-Young, F. Ding, F. Lipparini, F. Egidi, J. Goings, B. Peng, A. Petrone, T. Henderson, D. Ranasinghe, V. G. Zakrzewski, J. Gao, N. Rega, G. Zheng, W. Liang, M. Hada, M. Ehara, K. Toyota, R. Fukuda, J. Hasegawa, M. Ishida, T. Nakajima, Y. Honda, O. Kitao, H. Nakai, T. Vreven, K. Throssell, J. A. Montgomery, Jr., J. E. Peralta, F. Ogliaro, M. J. Bearpark, J. J. Heyd, E. N. Brothers, K. N. Kudin, V. N. Staroverov, T. A. Keith, R. Kobayashi, J. Normand, K. Raghavachari, A. P. Rendell, J. C. Burant, S. S. Iyengar, J. Tomasi, M. Cossi, J. M. Millam, M. Klene, C. Adamo, R. Cammi, J. W. Ochterski, R. L. Martin, K. Morokuma, O. Farkas, J. B. Foresman, and D. J. Fox, Gaussian, Inc., Wallingford CT, 2016.

17. Varetto, U., Molekel 5.4.0.8, Swiss National Supercomputing Centre, Lugano, Switzerland, 2009.

18. Hoggarth, E.; Young, E. H. P. Compounds related to thiosemicarbazide, Part VII. Benzylthiosemicarbazides *J. Chem. Soc.* **1950**, 1582–1584.

19. Schantl, J. G; Hebeisen, P.; Minach, L. α-Arylazoalkyl Isocyanates and Isothiocyanates by Potassium Permanganate Oxidation of 2,5,5-Trisubstituted 1,2,4-Triazolidin-3-ones and 1,2,4-Triazolidin-3-thiones *Synthesis* **1984**, 1984, 315–317.

20. Abdou, W. M.; Salem, M. A. I.; Sediek, A. A. Cyclic oxyphosphoranes in synthesis. A novel synthesis of oxathiaphospholenes, fused pyrimidines and aminooxyphosphoranes *Arkivoc* **2005**, xiv, 102–117.

21. Gribble, G.W. Indole Ring Synthesis: From Natural Products to Drug Discovery, *John Wiley & Sons, Ltd. Published* **2016**.

22. Tokuyama, H.; Yamashita, T.; Reding, M. T.; Kaburagi, Y.; Fukuyama, T. Radical Cyclization of 2-Alkenylthioanilides: A Novel Synthesis of 2,3-Disubstituted Indoles *J. Am. Chem. Soc.* **1999**, *121*, 3791–3792.

23. Kowol, C. R.; Nagy, N. V.; Jakusch, T.; Roller, A.; Heffeter, P.; Keppler, B. K.; Enyedy, É. A. Vanadium(IV/V) Complexes of Triapine and Related Thiosemicarbazones: Synthesis, Solution Equilibrium and Bioactivity. *J. Inorg. Biochem.* **2015**, *152*, 62–73.
